# Distribution of β-Lactamase Resistance Genes in the Environment: A Systematic Review

**DOI:** 10.3390/microorganisms14102204

**Published:** 2026-10-01

**Authors:** György Deák, Raluca Prangate, Monica Matei, Mădălina Boboc, Laura Lupu, Lavinia Ingrid Petrișor

**Affiliations:** 1 National Institute for Research and Development in Environmental Protection, Splaiul Independenţei 294, 060031 Bucharest, Romanialaura.lupu@incdpm.ro (L.L.); lavinia.petrisor@incdpm.ro (L.I.P.); 2 Doctoral School of Biotechnical Systems Engineering, National University of Science and Technology POLITEHNICA of Bucharest, Splaiul Independenței 313, 060042 Bucharest, Romania; 3 Academy of Romanian Scientists, Ilfov Street, No. 3, 050044 Bucharest, Romania

**Keywords:** antimicrobial resistance, β-lactamases, environmental health, global biosecurity, one health, wastewater-based epidemiology

## Abstract

The pervasive antimicrobial resistance (AMR) propagation in the environment constitutes a global concern, with wastewater being identified as a considerable contributor to the dissemination of resistance determinants. Introducing antibiotics and other pollutants into natural ecosystems via human activities has been considered a key factor in exacerbating this issue. This systematic review was conducted according to the Preferred Reporting Items for Systematic Reviews and Meta-Analyses guidelines and aims to assess the scientific literature published within the last ten years regarding the presence and diversity of antimicrobial resistance genes, especially β-lactamase-encoding genes, in environmental samples. A synthesis of data from peer-reviewed studies was conducted, investigating different environmental matrices—including water, soil, and sludge—using molecular methods for antibiotic resistance gene (ARG) detection and monitoring. This analysis underscores the commonly utilised molecular methods, such as polymerase chain reaction-based assays and next-generation sequencing. Thus, we show a high prevalence and diversity of β-lactamase genes in different environments, thereby underlining the crucial role of wastewater as an AMR reservoir and vector. Additionally, the analysis identifies crucial gaps in research, including the standardisation of sampling and analysis protocols, as well as limited surveillance. Research has indicated the requirement for the environment to be monitored and for the development of unambiguous techniques to track and prevent the propagation of antibiotic resistance from the environment into human-associated ecosystems. This information contributes to a more nuanced understanding of AMR’s dynamics in the environment and supports the development of improved monitoring and mitigation strategies.

## 1. Introduction

Antibiotics, one of the major discoveries of medicine in the twentieth century, are considered to be among the most crucial classes of drugs. Antibiotics were a big step forward in the fight against bacteria, saving millions of lives [1]. However, the number of infections caused by multidrug-resistant (MDR) bacteria has increased globally, and the threat of antibiotic-resistant infections is becoming a major health problem [2]. The wide use of antibiotics in human and veterinary medicine, along with improper administration, has led to this growing problem [3]. Antimicrobial resistance (AMR), also called the ‘silent pandemic’, is considered one of the major causes of global mortality, posing a significant threat to health systems [4,5]. Antibiotic resistance represents a real danger to life and the global economy, considering that it has become a burden greater than that of malaria or human immunodeficiency virus (HIV) [6].

The most crucial pathogens for public health, according to the World Health Organisation (WHO), are Gram-negative bacteria resistant to third-generation cephalosporins and carbapenems (broad-spectrum antibiotics), which represent β-lactams [3]. β-lactam antibiotics are among the most commonly used antibiotics in treating bacterial infections in humans and animals. Overuse of these antimicrobials has resulted in the development of bacterial resistance. Lactamases are one of the most studied enzyme families produced by bacteria that are resistant to β-lactam antibiotics such as penicillins, cephalosporins, cephamycins, and carbapenems [7,8]. β-lactam antibiotics contain a β-lactam ring that facilitates irreversible binding to penicillin-binding proteins (PBPs), inhibiting the cross-linking function of peptidoglycan during bacterial cell wall synthesis [9]. β-lactamases have been categorised according to their reported amino acid sequences into β-lactamases with active-site serine (SBLs) (classes A, C and D) and the zinc-dependent metallo-β-lactamases (MBLs) (class B). The major enzyme families include TEM, SHV, CTX-M, and KPC (class A); NDM and VIM (class B); and CMY and ADC (class C). Class D enzymes are called oxacillinases. Other enzymes that hydrolyse β-lactamases, such as KPC (class A), NDM (class B), and OXA-48 (class D), are proliferating globally [10].

Recently, AMR’s impact on the environment has gained recognition as a substantial global health concern [11]. The rise in antibiotic resistance acquisition by bacteria is the result of excessive antimicrobial consumption for medical and agricultural reasons. The primary practices that result in the current ‘antibiotic pollution’ are the overuse of antibiotics for treating pathogenic microbial diseases and the inadequate treatment of waste containing non-metabolised drugs and their residues [12]. Wastewater treatment plants (WWTPs) act as key reservoirs and amplification points for antibiotic-resistant bacteria (ARB), driven by three interconnected factors. Firstly, WWTPs receive concentrated and highly diverse bacterial inputs from clinical, agricultural and domestic sources. This creates a mixed microbial community in which resistant and susceptible strains coexist in proximity. Secondly, residual antibiotics, biocides, and heavy metals entering WWTPs are often present at sub-inhibitory concentrations. These concentrations are insufficient to eliminate bacteria, but are high enough to give resistant strains a selective advantage over susceptible ones. A third factor is that the combination of high bacterial density and continuous antimicrobial exposure creates favourable conditions for horizontal gene transfer, particularly through plasmid conjugation. This allows resistance genes to spread not only within bacterial species, but also across them. Together, these factors establish WWTPs as active environments in which resistance is generated, amplified and released into receiving waters and downstream ecosystems [13,14,15]. These plants, which are considered to be a reservoir of antibiotic-resistance genes (ARGs), do not yet have the technology to eliminate ARB, ARGs, and antibiotic residues.

Researchers have begun using improved methods to test environmental samples for AMR; however, some gaps in the research still exist. This makes it harder to understand and answer questions such as how AMR influences the environment, how to prevent it, and how it is transmitted. Several key factors have been identified as potential solutions, which include enhancing monitoring strategies, developing technologies to treat and halt AMR spreading in wastewater, and using scientific evidence for a global response to AMR. Implementing such strategies will contribute to cultivating international collaboration and improving the existing scientific knowledge concerning AMR’s policy implications. Additionally, scientific studies are poorly documented owing to the lack of systematic investigations of bacterial resistance in downstream surface waters contaminated with wastewater enriched with ARB [16]. Additionally, a requirement exists to evaluate the pollution potential of wastewater and sewage sludge reuse in agriculture or other environments, considering the high ARB levels detected in wastewater [17].

While solutions are yet to be discovered, some technologies have been explored to counteract the effects of antibiotic resistance. Strategies to combat antibiotic resistance could include: anticolonisation/decolonisation, reduction in antibiotic use, re-sensitisation of bacteria, and interest in nutritional dietary interventions that may decrease ARG and ARB colonisation and spread in the human gut [18,19,20]. Detailed knowledge of the molecular mechanisms of bacterial resistance to antibiotics facilitates new molecule development to eradicate pathogens that rapidly adapt to the present treatments [21]. Furthermore, new scientific advances allow the development of new antibiotic classes that decrease antibiotic resistance [1]. A global action plan based on the One Health approach to combat AMR needs to be implemented in line with the Food and Agriculture Organisation (FAO)-World Organisation for Animal Health (OIE)-WHO guidelines [22]. According to the ‘One Health’ approach, decreasing AMR threats worldwide requires an expensive and long-term effort. Although AMR is impossible to avoid, its decrease can be achieved by implementing strategies aimed at supporting awareness programs for the responsible and prudent use of antimicrobials by human, animal, and plant health professionals due to the natural evolutionary process. Additionally, Sustainable Development Goal 6 (SDG6) supports the requirement for investment in ambitious research and development of new technologies to combat AMR, advocating for research and development of new antibiotics, as well as for the collaboration of all sectors involved [23].

This review aims to systematically analyse the scientific literature published primarily in the last ten years to examine the ARG presence and diversity, in particular those encoding β-lactamase, in environmental samples. We will review the molecular methods utilised to detect and monitor resistance genes. We will discuss the current trends and research gaps in this area. This will help in understanding wastewater’s role in the spread of AMR in the environment. This study makes a novel contribution to the literature by providing a comprehensive and up-to-date synthesis of β-lactamase-encoding ARGs in environmental matrices, with a specific focus on methodological approaches and data gaps relevant for One Health monitoring strategies.

## 2. Materials and Methods

A detailed review of the existing research was conducted using three electronic databases, PubMed, Scopus, and Web of Science (WoS), on 26 June 2026, to find the relevant articles for this review. The Preferred Reporting Items for Systematic Reviews and Meta-Analyses were used for this purpose. The search strategy included articles that were written in English within the last ten years and excluded preprints and reviews. The keywords associated with ‘wastewater’, ‘environmental monitoring’, and ‘AMR’ identified several article records. Table 1 reveals the three criteria as the search keys and the number of articles found in the multidisciplinary literature. The decision to use this approach was informed by the extensive research findings on this subject that were identified during the literature review. Table 1 outlines the application of the method, which facilitated the decrease in the number of records identified in the three databases. This allowed the identification of a suitable set of articles for use. This systematic review was not registered, and a protocol was not prepared. The review was divided into three stages, with each focusing on a core concept. The first stage returned a large number of results: 417,916 articles in PubMed, 1,213,916 in WoS, and 1,227,693 articles in Scopus. The second stage focused on β-lactam antibiotic resistance and returned 16,554 articles in PubMed, 19,337 in WoS, and 29,384 in Scopus. The third stage involved searching for terms related to the detection and methodology of β-lactam antibiotic resistance. This retrieved 465,328 articles from PubMed, 558,625 articles from WoS, and 611,675 articles from Scopus. In the end, the three stages were combined to retrieve articles addressing all three topics. Consequently, fewer articles were obtained: 498 in PubMed, 668 in WoS, and 1081 in Scopus. These represent the final pool of studies relevant to the review’s specific research question. A total of 2247 articles were returned after the established criteria in Table 1 were applied. After removing the duplicates, 1273 articles remained to be analysed. Three reviewers conducted the manual selection independently, which involved downloading the following information from the database: title, abstract, keywords, authors, and references. This information was then centralised in a Microsoft Excel spreadsheet. Following a review of the titles and abstracts of the returned articles, 856 were excluded. Furthermore, 82 articles were eliminated because they did not align with the objectives of this review. The reasons for elimination included the following: lack of environmental samples; absence of β-lactamase genes; use of different methods; unclear results; ineligible publication type; and being written in a language other than English. 13 articles were not included due to limited access to the full text. Therefore, 322 studies were identified as relevant to the aims of this review. The PRISMA diagram (Figure 1) shows the selection process for the scientific papers used in the present study. Results were presented in tabular form and organized according to surveillance period, sample type, country, target ARGs, methods used and obtained results. The results were then synthesized narratively. All the data were compiled and analysed using Microsoft Excel (Microsoft Office suite). Geographic mapping was performed in QGIS (V3. 40. 11) using cartographic design tools, based on the raw data processed in Microsoft Excel [24]. The heatmap depicting gene distribution across sampling sources was generated in RStudio (V. 2026.01.2+418) using R packages “readxlV1.5.0.1”, “dplyrV1.2.1”, “tidyrV1.3.2”, “stringrV1.6.0”, and “ggplot2V4.0.3” [25].

## 3. Results and Discussion

### 3.1. Overview of the General Characteristics and Detection Methods of AMR in the Included Studies

ARGs have been studied in different environments, including soil, wastewater, surface water, and drinking water [26]. Thus, antibiotic resistance surveillance has become a useful tool in discovering new emerging AMR strains, observing trends and seasonal patterns, and looking into the distribution of antibiotic-resistant microorganisms and ARGs [27]. Surveillance methods for detecting ARGs in aquatic and terrestrial environments have also improved, considering the recent advancements in molecular biology, bioinformatics, and environmental science [28]. The primary methods that are used in ARG monitoring and characterisation include culture-based methods, molecular approaches such as polymerase chain reaction (PCR) (e.g., standard PCR, quantitative PCR (qPCR), multiplex PCR), DNA microarrays, and sequencing [29]. Furthermore, bioinformatics tools were developed to help with high-throughput data processing, as well as the extraction, annotation, and interpretation of ARG-associated information [28].

Culture-based methods are presently considered the gold standard for AMR identification in clinical settings, and they can determine the minimum inhibitory concentration (MIC) or minimum bactericidal concentration (MBC). Besides serial dilution and broth microdilution, other less labor-intensive methods, such as disk diffusion and strip diffusion, exist [30]. However, PCR-based techniques are more precise in determining ARGs. Although standard PCR was the primary method for identifying ARGs, real-time quantitative PCR (RT-qPCR) has since taken its place owing to its efficiency [29]. The difference between conventional PCR and RT-qPCR is that the DNA sequence can be monitored during the reaction with the help of fluorescent dyes in RT-qPCR, eliminating the requirement for electrophoresis. Multiplex PCR is another useful method for simultaneously detecting multiple resistance genes in one reaction [31]. Multiplex assays often focus on the genes with similar phenotypes, such as those that confer resistance to β-lactamases [32]. High-throughput qPCR (HT-qPCR) has been recommended for handling a high number of samples and multiple gene quantification. Another newly developed technology is digital PCR (dPCR), known for its sensitivity and ability to perform absolute quantification [26]. Although this method is often more expensive, it has been used to detect ARGs in different environments and study ARG mobility [33].

Additionally, sequencing has been a valuable tool in AMR surveillance. Recent improvements in next-generation sequencing (NGS) technology have decreased the costs and enriched the bacterial sequence-related data [34]. Several platforms have been utilised for analysing resistance genes, including NGS platforms such as HiSeq, Sequencing by Oligonucleotide Ligation and Detection, PacBio, and Oxford Nanopore [29]. Assembly algorithms can be used for aligning the reads obtained through whole-metagenome sequencing to detect ARGs. Tools and resources such as the Comprehensive Antibiotic Resistance Database (CARD), ResFinder, and Mustard are useful for curating sequencing data and identifying mutations. Additionally, metagenomic and sequencing studies can be utilised to determine phylogenetic diversity and evaluate the potential loss or gain of function of ARGs [34]. The obtained information can vary depending on the metagenomic approach (function- or sequence-based). Complete genome sequencing of the environmental samples provides a comprehensive view into the genetic diversity of a microbial community. Contrastingly, functional metagenomics often uses cloning environmental DNA into expression vectors to identify novel resistance genes by phenotypic analysis [35].

Supplementary Table S1 provides an overview of the most commonly used techniques for studying ARGs and the most frequently targeted β-lactam resistance genes. Generally, studies have favoured a combination of PCR and culture-based methods due to their accessibility. However, some studies used metagenomic analysis to gain a more comprehensive understanding of the environments under study. The majority of the identified resistance genes were found in bacterial isolates cultured from environmental samples, such as lakes, rivers, sediments, and WWTPs. The ARGs most frequently detected in the analysed studies, with a prevalence of over 10% in the analysed studies were: *blaCTX-M*, *blaTEM*, *blaOXA*, *blaSHV*, *blaKPC*, *blaNDM*, *blaVIM*, *blaGES*, *blaIMP*, *blaAmpC*, and *blaCMY*. Additional β-lactamase gene variants, including *blaCIT*, *blaPSE*, *blaSIM*, *blaDHA*, *blaFOX*, *blaACC*, *blaMOX*, *blaVEB*, *blaPER*, *blaACT*, *blaL*, *blaCfxA*, *blaORN*, *blaIMI*, *blaSFO*, *blaSPM*, *blaEBC*, *blaOKP*, *blaNPS*, *blaTLA*, *blaSME*, *blaOCH*, *blaGIM*, *blaADC*, *blaZ*, *blaPOM*, *blaEC*, and *blaSRT*, were also screened across the surveyed studies, but with a lower frequency.

According to Supplementary Table S1, 34% of the analysed research papers were conducted in Asia, followed by Europe with 29%. The remaining studies were conducted in Africa (57 out of 322), North and South America (35 and 26 out of 322, respectively), Australia (3 out of 322), and Antarctica (1 out of 322), with four of the total studies being conducted internationally (Figure 2a). Also, Figure 2b summarises the distribution by year of the studies published and reviewed in the present research. The volume of published research on environmental β-lactamase genes increased between 2016 and 2026, rising from 16 studies in 2016 to a peak of 43 studies in 2023. Although publication output declined to 12 studies in 2018, it subsequently resumed an upward trend. The number of studies remained consistently high in 2024 and 2025 (41 annually). The lower number of studies in 2026 is due to the incomplete dataset for that year at the time of analysis, rather than a decline in research activity. The distribution of samples across the reviewed studies was biased towards wastewater-related matrices. Wastewater, river water and WWTP samples together accounted for around 71% of all samples analysed. Surface waters, lake waters and sewage constituted a secondary category, representing around a fifth of the dataset. Sediment, soil and biofilm samples were also underrepresented. This distribution suggests that environmental surveillance efforts continue to prioritise accessible point sources, such as wastewater treatment infrastructure, over terrestrial compartments. It also indicates that soil, biofilm, and other underexplored environments represent critical gaps in current monitoring strategies for the dissemination of antimicrobial resistance (Figure 3). The distribution of genes across sample matrices varied across the included studies, suggesting differences in ARG profiles (Figure 4). All 11 genes except *blaAmpC* exhibited the highest frequency in samples from wastewater, rivers and WWTPs, with *blaOXA* being the most frequently reported in aquatic and wastewater-associated matrices. *blaCTX-M*, *blaTEM*, *blaOXA* and *blaSHV* were also commonly reported in lakes, sewage, soil, and surface water. Biofilm and canal water showed lower detection frequency across studies. Nevertheless, it is worth observing that the findings reflect study-level reporting frequency and may be influenced by differences in the representation of sample types among studies.

A selection of the most crucial β-lactamase classes was conducted to analyse ARG prevalence utilised and detected in the studies analysed for this current review. Furthermore, β-lactams are one of the most frequently used classes of therapeutic agents against infections. Several bacterial strains are now resistant to this antibiotic class. β-lactamases, the enzymes that hydrolyse β-lactams, have undergone constant genetic modifications associated with regulatory regions, mutations in promoter sequences, and integrated promoter sequences linked to high gene expression [36]. The *blaTEM* gene is part of the extended-spectrum beta-lactamase (ESBL) family. TEM β-lactamase can inactivate narrow-spectrum penicillins and cephalosporins [37]. The enzyme, along with *blaSHV*, has diminished in prevalence over the years, being replaced by the ESBL gene *blaCTX-M* [38]. This gene confers resistance to the antibiotic cefotaxime, and the prevalence of the *blaCTX-M-15* group has increased worldwide despite it being primarily reported in Europe. *blaCTX-M* genotypes show considerable diversity and have been identified in animals and humans [39]. SHV-lactamases are commonly found in Gram-negative bacteria and can hydrolyse cephalosporins and other antibiotics owing to the evolution of SHV enzymes from narrow to extended spectrum [40]. Among these three enzymes, *blaTEM* has fewer newly reported variants than *blaSHV* and *blaCTX-M* [38]. KPC (class A), NDM (class B) and OXA-48 (class D), are another group of carbapenem-hydrolysing enzymes. The *blaKPC* gene is responsible for *Klebsiella pneumoniae*’s resistance to carbapenems [10]. *blaNDM* gene is frequently found in *K. pneumoniae* and *E. coli* and codes for the New Delhi metallo-β-lactamase, which can hydrolyse almost all β-lactams. Given that no treatment exists for pathogens that have the gene or other NDM inhibitors, their existence is considered a public health threat [41]. The *blaOXA* is among the first identified resistance genes, initially thought to be found only on plasmids (*blaOXA*-23, *blaOXA*-40 and *blaOXA*-58). However, recent findings have identified variants on the *A. baumannii* (OXA-51-like) or *Acinetobacter* chromosome. While they demonstrate resistance to cephalosporins and penicillins, they have also shown resistance to carbapenems [42]. Other enzymes that hydrolyse carbapenems are the GES, IMI, VIM, and IMP families. The *blaGES* genes are not as common in clinical isolates but have been observed in aquatic environments [43]. The *blaIMI* gene, although detected globally, is often linked to the *Enterobacter* genus [44]. IMP and VIM enzyme families are part of the MBL class and have most of the time been detected in *Pseudomonas aeruginosa*. However, they can be isolated from *Acinetobacter* spp. and Enterobacterales [41]. Beyond the discussed enzymes, another crucial resistance gene is *blaAmpC*, which is most often encoded on chromosomes but can also be plasmid-mediated. *blaAmpC* gives resistance to different antibiotics such as cephalothin, cefazolin, cefoxitin, most penicillins, and β-lactamase inhibitor-β-lactam combinations [45]. The *blaCMY* gene is one of the most globally prevalent plasmid-mediated AmpC genes, derived from the chromosomal AmpC of *Citrobacter freundii*. The *blaCMY-2* variant is widespread among *E. coli*, *Salmonella enterica*, and *K. pneumoniae* [46]. The *blaDHA* gene has been reported in *K. Pneumoniae* and *E. coli* and is known for retaining inducible expression via an adjacent ampR regulatory gene [47]. *blaFOX* and *blaMOX* have both been observed in *Aeromonas* spp. chromosomal ancestors and confer characteristic resistance to cefoxitin and moxalactam, respectively, distinguishing their phenotype from that of many ESBLs that spare cephamycins [48,49]. *blaACT* and *blaEC* are *blaAmpC* genes that are found naturally in *Enterobacter cloacae* complex organisms. These genes are usually under inducible regulatory control, but mutations can arise in response to selective antibiotic pressure, which can lead to treatment failure [50]. *blaACC* was analysed in several species such as *K. pneumoniae*, *Proteus mirabilis*, and *E. coli*, among others. This gene exhibits weak cefoxitin hydrolysing activity, which makes phenotypic detection difficult [51]. *blaCIT* was frequently observed in *Citrobacter freundii* isolates, as well as in *E. coli* and *K. pneumoniae*. It is one of the most frequently detected genes in the *blaAmpC* family. *blaEBC* likewise derives from a chromosomal ancestor, in this case the intrinsic cephalosporinase of *E. coli* [49,52,53]. The *blaADC* gene is part of the class C beta-lactamase gene family and is found primarily in non-fermenting Gram-negative pathogens. It is the intrinsic chromosomal cephalosporinase of *A. baumannii*, and its expression can be significantly increased by upstream insertion sequences, such as ISAba1 [54]. *blaOHC* genes, also part of class C, are chromosomally encoded in *Ochrobactrum anthropi* and have been described in *Brucella anthropi*, with extended-spectrum activity [55]. Among the ESBLs, *blaPER* and *blaVEB* both confer strong resistance to ceftazidime and aztreonam and are frequently disseminated via mobile genetic elements. PER-type enzymes are concentrated in *P. aeruginosa*, *A. baumannii*, and *Salmonella* spp. in the Middle East and parts of Asia, while *blaVEB* genes are commonly integron-associated and prevalent in isolates from Southeast Asia [56,57,58]. The *blaTLA-1* gene was first reported in a clinical *E. coli* isolate from Mexico, while *blaIBC-1* was first identified in *E. cloacae* from Greece and is closely related to the GES family. *blaSFO-1*, first identified in *E. cloacae* from Japan, is closely related to the chromosomal AmpA β-lactamase of *Serratia fonticola* and encodes an Ambler class A ESBL capable of hydrolysing oxyimino-cephalosporins [59,60,61]. Of the narrow-spectrum penicillinases, *blaPSE* and *blaCARB* are closely related. They were often harboured by *Pseudomonas aeruginosa* and *Salmonella* species before more recent resistance mechanisms emerged [62,63]. *blaROB*, first characterised in *Haemophilus influenzae*, hydrolyses aminopenicillins and remains a secondary, less prevalent alternative to *blaTEM*-type resistance in this species [64]. The classical staphylococcal penicillinase gene, *blaZ*, remains one of the most epidemiologically significant class A beta-lactamase determinants. It is present in the majority of contemporary clinical *Staphylococcus aureus* isolates and is tightly regulated by the adjacent signal transduction system, *blaR1/blaI* [65]. *blaOKP* represents chromosomally encoded class A enzymes characteristic of *K. pneumoniae* and *Ochrobactrum anthropi*, respectively, contributing to baseline aminopenicillin resistance in their native hosts [66]. *blaCfxA*, which was first identified in *Bacteroides vulgatus*, is notable for its ability to hydrolyse cefoxitin despite being assigned to class A. This atypical property can complicate the classification of anaerobic isolates based on phenotype [67]. Another family that represents clavulanate-susceptible serine carbapenemases and was first identified in *Serratia marcescens* is *blaSME*. This family hydrolyses carbapenems, penicillins, and cephalosporins while remaining inhibited by clavulanic acid, a phenotypic feature that distinguishes them from the metallo-carbapenemases despite the shared carbapenem-hydrolysing activity [68,69]. The *blaNPS* gene was first identified in a *Pseudomonas aeruginosa* clinical isolate. While the NPS enzyme cannot hydrolyse stable β-lactams, it can break down carbenicillin, azlocillin, cefoperazone and cefsulodin [70].

The studies analysed in this review reported various types of β-lactamase genes. Among these, *blaCTX-M* was the most frequently detected (65.28% of country-study records), which highlights its significant relevance in the current context of AMR. Additionally, the *blaTEM* and *blaOXA* genes were identified with prevalence rates of 60.24% and 50.74%, respectively. This confirms the persistence and widespread occurrence of these genetic determinants in different environments. The *blaSHV*, *blaKPC*, and *blaNDM* genes were reported with 40.36%, 36.50%, and 33.83% frequencies, respectively, with *blaKPC* and *blaNDM* representing carbapenemase-encoding determinants, while *blaSHV* is more broadly recognised as an ESBL/broad-spectrum β-lactamase family. Other clinically significant genes associated with reduced carbapenem susceptibility were also widely distributed, including *blaVIM* (21.66%), *blaGES* (18.69%), and *blaIMP* (16.02%) (Figure 5). AmpC β-lactamase genes were represented by *blaAmpC* (12.17%), *blaCMY* (10.68%), *blaCIT* (8.31%), *blaPSE* (7.42%), *blaSIM* (6.23%), *blaDHA* (5.93%), *blaFOX* and *blaACC* (5.34% each), *blaMOX* (4.45%), and *blaVEB* (4.15%). Additional β-lactamase gene families were detected at lower frequencies, including *blaPER* (3.86%), *blaACT* (3.56%), *blaCfxA* (2.08%), *blaORN*, *blaIMI*, *blaSFO*, *blaSPM*, *blaEBC*, *blaOKP*, *blaNPS*, *blaTLA*, *blaSME*, *blaOCH*, and *blaGIM*. The lowest prevalence (≤0.59%) was observed for *blaGIM*, *blaADC*, *blaZ*, *blaCARB*, *blaPOM*, *blaEC*, and *blaSRT*. The wide range of β-lactamase determinants identified highlights the complexity of the environmental resistome and shows that multiple resistance mechanisms are being transmitted within the analysed environmental samples. The simultaneous presence of clinically relevant genes, such as *blaCTX-M*, *blaKPC*, *blaNDM* and *blaVIM*, in environmental matrices is a cause for concern in terms of public health. This indicates the ongoing dissemination of resistance determinants between clinical and environmental reservoirs, highlighting the urgent need for comprehensive, continuous surveillance strategies.

Figure 6 shows the geographical distribution of the included studies and the β-lactamase genes investigated. Pie charts show the β-lactamase genes investigated in each country, with the size of the pie corresponding to the number of studies included. The most frequently reported ARGs were identified by ranking genes according to the number of studies reporting their occurrence. Only ARGs reported in more than 10% of the included studies were retained, with a 10% prevalence threshold applied. ARGs apart from the top 11 shown were grouped as “others”. The distribution of the investigated gene groups showed geographical diversity, with the most frequently detected genes being *blaCTX-M*, *blaTEM*, *blaSHV* and *blaOXA*. The number of studies varied considerably across countries, with most being concentrated in China (26), India (24), the U.S.A. (18), Brazil (16) and South Africa (14). The geographical distribution of reported ARGs revealed differences in the range of ARGs identified in different countries. In general, countries that had conducted a greater number of studies, such as the U.S.A., Brazil, China, India and South Africa, exhibited a broader profile. In contrast, countries that had conducted fewer studies tended to show a more restricted profile comprising widely reported genes, such as *blaTEM*, *blaCTX-M* and *blaSHV*. Several countries exhibited all 11 genes, suggesting a wide range of reported ARGs in these locations. This pattern was observed in seven countries where a greater number of studies were conducted: Brazil (16), China (26), Croatia (8), Germany (11), India (24), South Africa (14), and the USA (18). Studies in India and China have most often reported the detection of *blaNDM*, appearing in 15 and 11 studies, respectively, followed by *blaCTX-M*, *blaTEM*, *blaOXA* and *blaSHV*. *blaCTX-M* was the most commonly detected gene in the USA, Nigeria, Spain and Japan, whereas *blaTEM* and *blaCTX-M* were jointly the most present genes in Bangladesh, Germany, Iran, Portugal and Tunisia. These findings highlight their wide distribution across the countries included in this review, as well as their frequent inclusion in ARG surveillance studies. An exception to the overall geographical pattern was observed in Brazil, where *blaKPC* was the most frequently reported ARG. Brazil also showed the highest number of studies reporting less frequently reported genes that were classified as “others”, such as *blaVEB*, *blaACC* and *blaSPM*.

These geographical patterns align with the previously reported distributions of clinically important ARGs. Similar to the findings of the present review, the widespread presence of *blaNDM* has been associated with South Asian countries such as Bangladesh, India, Pakistan and China [71,72]. The predominance of *blaKPC* in Brazil has also been observed in previous research, being considered endemic in the region [73]. The frequent reporting of *blaCTX-M*, *blaTEM*, *blaSHV*, and *blaOXA* in the present review is consistent with previous environmental research that has identified these genes as among the most commonly detected β-lactamase determinants [74]. Numerous studies have identified the prevalence of genes such as *blaCTX-M*, *blaOXA*, *blaKPC* and *blaTEM*, mainly due to human antibiotic practices such as misuse, overuse and unregulated over-the-counter access in different regions [75,76]. Inadequate antibiotic stewardship allows resistant strains to persist and accumulate. Hospitals act as breeding grounds for their further spread through patient transfers and sewage systems [77]. Therefore, resistance genes may become prevalent in regions with high antibiotic use [78].

### 3.2. Wastewater Contamination Sources and Technologies for Treating Varying Water Types

#### 3.2.1. Clinical Environments

Antibiotic resistance, formerly considered a clinical problem, has become a complex public health concern with different links between human, animal, and environmental health. The mechanisms that contribute to ARG spreading are: direct or extracellular dissemination via horizontal gene transfer (HGT), organic matter enrichment of ARGs, and the dissemination of selective factors (e.g., antibiotics) [79]. According to the WHO, the presence of carbapenem-resistant bacteria has been reported in waters worldwide [80]. In terms of emerging AMR threats, genes that confer resistance to cephalosporins, especially gene variants that code for ESBLs, such as *blaCTX-M*, are of primary concern [81]. These antibacterial-resistance determinants are increasingly identified in sites that extend beyond hospitals, such as wastewater, pets, wildlife, and even food, which confirms the urgent requirement for a comprehensive “One Health” approach [82]. *blaCTX-M* genes have been mobilised into plasmids of environmental bacteria, rendering them omnipresent. Frequent insertion sequences and transposons such as ISEcp1, IS26 and Tn2 also contribute to the dissemination of the *blaCTX-M-1* and *blaTEM* gene regions among different human and animal reservoirs [83]. These systems are efficient and are extremely mobile, offering resistance as a means of surviving and thriving against selective pressures.

Recent scientific research has shown several sources of environmental pollution with AMRs, raising major public health concerns. MDR and ESBL genes are present in up to 60% of bacterial isolates harvested from WWTPs [84]. Additionally, Gelalcha et al. researched farm environmental isolates, of which 99.4% were MDR and ESBL-resistant [85]. Moreover, feed and water are crucial reservoirs of MDR organisms for livestock. Antibiotic-resistant *E. coli* strains can also be conveyed onward in the food chain via farming [86,87]. Pharmaceutical and agricultural waste participate in the process and serve as an antibiotic resistance reservoir, which is released into aquatic and terrestrial systems [88].

#### 3.2.2. Domestic Wastewater

Besides medical-related environments, antibiotic resistance has been detected in sources associated with domestic activities and agriculture. For instance, according to Li’s study, these genes have been found in honey [89]. Sun et al. suggest that the interactions between humans, animals, and the environment, concerning pollination and the use of agricultural chemicals, may influence ARG presence in honeybees [90]. Recent domestic and environmental studies have shown that cats and dogs, in particular, are MDR bacterial reservoirs and may serve as carriers. According to Yuan et al., shared habitats may play a role in the bidirectional *E. coli* strain exposure that harbors resistance genes, e.g., ESBLs, in humans and animals. Furthermore, 54.81% (74/135) of isolates were MDR *E. coli*, underscoring the risk of spreading AMRs to other species [83]. In the United Kingdom (UK) and Portugal, Menezes et al. detected common ESBL- and AmpC-producing *Enterobacteriaceae* in companion animals and their owners, informing potential routes of antibiotic resistance transmission via routine contact [91]. Studies in Germany and Korea have identified third-generation cephalosporin-resistant *E. coli* in domestic animals, with the most common gene group being *blaCTX-M* [92,93]. Other studies reported that wild aquatic birds, such as gulls, and wild ungulates can act as ARB hosts [94,95]. Once exposed to human-impacted environments, they acquire resistance and further transmit it to domestic animals and potentially humans. Among wild boars, 75% of the *Salmonella* isolates were resistant to at least one antimicrobial agent. Of these, 15.7% were MDR, and streptomycin had the highest resistance level among the aminoglycosides [96]. The high *blaCTX-M-1* gene prevalence in bird samples may suggest transmission from chickens since *blaCTX-M-1* has long dominated poultry production in Europe [97].

#### 3.2.3. Agricultural and Industrial Effluents

The several wastewater pollution sources necessitate ongoing measures to stop AMR spread. Anthropogenic activities, agricultural practices, industrial development, and high antibiotic consumption, along with the diversity of pharmaceutical products, have caused a worrying increase in antibiotic-resistant microbial strains. Antibiotics are considered emerging pollutants due to their harmful environmental effects and persistence in ecosystems [98]. Therefore, collective, science-based action is necessary if the tide of increasing AMR is to be stopped for future generations. Most of the pollutant load is decreased during the treatment stages used by wastewater treatment plants. However, antibiotics remain in aquatic ecosystems because conventional techniques of wastewater treatment at municipal plants cannot eliminate resistance genes and bacteria. These antibiotics are subsequently released into the environment via effluents [99]. Thus, the objective of wastewater treatment plants is to reduce pollution by treating influents and releasing effluents into the environment. Several physical, chemical and biological methods, as well as technologies involving advanced oxidation processes, adsorption, membrane filtration and coagulation have been tried for the remediation of microbial activity in different water matrices [100]. The various technologies that have been implemented are stabilisation ponds, lime or water treatment, reflux filters, the construction of wet areas, the development of decentralised wastewater treatment systems, the use of deflected anaerobic reactors, chlorination methods and ultraviolet-based (UV) technologies [101,102,103].

#### 3.2.4. Technologies for the Removal of ARB and ARGs from Wastewater

Several remediation technologies were utilised (Table 2) to eliminate ARGs and ARBs such as *E. coli* from water samples: adsorption through magnetic graphene oxide (MGO), natural coagulation with *S. potatorum*, precipitation of iron oxide on biochar, ozone method, chlorination, and ultraviolet light irradiation [104,105,106,107]. Rommozzi et al. compared the effectiveness of Photo-Fenton treatment and the Solar water disinfection (SODIS) method in decreasing *E. coli* bacteria in water samples. They concluded that Photo-Fenton treatment is a robust process that can inactivate 99.99% of *E. coli* in surface waters within an optimised time frame compared to the SODIS method [108]. Similarly, Rubio et al. stated that the Photo-Fenton technique combined with H_2_O_2_/UV is more effective at inactivating *E. coli* in seawater than UV treatment alone [109]. Simultaneously, Manasa et al. obtained a 1.302 log (95.01%) and 6.426 log (99.00%) decrease in *E. coli* after exposure to sunlight for 180 min using photocatalysis technology with Ce-TiO_2_ and B-TiO_2_ [110]. Using the same method, Li et al. obtained complete inactivation of *E. coli* (10^7^ CFU/mL) with Bi_2_MoO_6_/Ag-AgCl after exposure for 30 min [111]. The electrochemical oxidation technique was also used for wastewater treatment and achieved log reductions of 3.8 and 2.9, respectively, in heterotrophic bacteria by increasing the reaction time (T) to T20 and the current density (D) to D80. However, the study also emphasised that T treatment was less effective at eliminating ARGs, despite being more efficient at inactivating bacteria [112]. Furthermore, bacterial inactivation occurred after 30 and 6 min, respectively, when the two WWTP samples were subjected to electrochemical disinfection [113]. Ivankovic et al. used metal-exchanged zeolites to decrease the number of Gram-negative bacteria, such as *Acinetobacter baumannii*, in wastewater. The log reduction was 0 for natural zeolite (NZ), 0.38 ± 0.09 for copper (CuNZ), and 100% for silver (AgNZ). Therefore, NZ had no obvious effect on bacteria, CuNZ showed slight antibacterial properties, and AgNZ was the most effective treatment [114]. González et al. evaluated the effect of ultraviolet lamps on bacteria from constructed wetlands. The effectiveness of the treatment depended on the influent’s level of total suspended solids (TSS). The log reduction in faecal coliforms was 2.03 log when the TSS level was 26.7 mg/L, and it decreased to 0.33 log when the TSS level increased to 34.7 mg/L [115]. One study focusing on decreasing ARGs revealed that ultraviolet light-emitting diode (UV-LED) and UV light are efficient against ARB in wastewater, with over 99.9% being eliminated after UV treatment for 1–3 min and UV-LED treatment for 3 min. For ARG removal, using UV light with a 254 nm wavelength, values of 0.2–1.4 log10 were obtained, whereas with a 280 nm wavelength, values of 0.1–1.3 log_10_ were obtained [116]. De Sanctis et al. used a granular sequential biofilter reactor (SBBGR) technology to decrease *Clostridium perfringens* and *E. coli*. The reactor operated in several 6 h cycles, which led to a decrease of 1.4 log_10_ units for *C. perfringens* and 3 log_10_ units for *E. coli* [117].

The success rate of each method in decreasing ARB differs [100]. Until now, WWTPs have been designed to treat organic compounds, nitrates, and phosphates, but not ARGs [118]. Therefore, the low efficiency of water treatment techniques has been previously reported. For instance, the study by Amos et al. demonstrated the inefficiency of a wastewater treatment plant with three treatment sections: decantation, sludge tartrate and filtration. ARBs were identified in samples taken upstream and downstream of the plant, despite it being considered one of the most rigorous treatment methods in the UK [119]. Zanotto et al. showed the presence of *E. coli* before and after biological treatment followed by peracetic acid disinfection at a treatment plant in Milan. The sample tested positive for *E. coli* that was resistant to ampicillin and chloramphenicol [120]. These observations reinforce the notion that removing antibiotics from water necessitates more effective treatment methods. Some instances of such methods include advanced oxidation processes, constructed wetlands, and UV radiation, among others [121,122].

However, complete ARG removal remains challenging for industrial WWTPs. It is concerning that these emerging substances remain in the effluent following treatment, thus being released into the environment and potentially causing infections that are challenging or even impossible to treat in humans and animals [121,123].

### 3.3. Assessment Tools for the Impact of Discharging Treated Wastewater and Sludge into the Environment

In the context of remediating microbial activity from different water sources, several methods and technologies have been used that have contributed to the spread of ARB and ARGs. Wastewater treatment removes pollutants and contaminants from water before it is released back into the environment. However, this process can have negative environmental consequences that are often overlooked. Since resistance genes can be transferred to other bacteria via HGT in downstream environments, the presence of these genes in effluents and wastewater sludge poses a substantial concern [124,125]. Understanding these effects allows for the development of more sustainable and environmentally friendly wastewater treatment practices. Wastewater treatment is an important process that protects the environment and promotes public health by purifying contaminated water before releasing it into nature. Additionally, the sludge disposal system is an integral part of the wastewater treatment process, playing a main role in its effectiveness [126]. Several studies have evaluated the environmental impact of WWTPs using different impact evaluation tools, such as life cycle assessment (LCA), environmental impact quotient (EIQ) and grey water footprint (GWF) [127,128].

#### 3.3.1. Life Cycle Assessment

LCA is utilised to measure a WWTP’s possible lifetime environmental impacts. This method involves identifying and analysing different environmental impacts, such as primary resource use, greenhouse gas emissions, waste production, resource depletion, and air, water, and soil pollution [127,129]. Applications such as Umberto, Gabi, OpenLCA, and Semipro have been developed to assess these factors and provide comprehensive analyses of the environmental effects [130]. With the help of programs such as OpenLCA, LCA provides a thorough assessment of the environmental performance of complex systems, such as WWTPs. This information can be utilised to promote continuous improvement and more sustainable results, resulting in long-term success. A study by Ata et al. at a WWTP in Jordan emphasised LCA’s importance in assessing the ecological impact of these facilities [127]. Alibekov et al. used LCA to quantify the environmental impact of microplastic emissions from WWTPs. Microplastics accounted for 94% of the total impact of the plant, indicating a harmful effect on aquatic species per cubic meter of discharged wastewater. This exceeded the impact of other contaminants, such as heavy metals and nutrients, by at least two orders of magnitude [131]. Additionally, this evaluation method has been utilised in evaluating antibiotic mycelial residues, revealing how the management of these pollutants can have a different environmental impact. The study reported that gasification was the best alternative to dispose of AMRs, whereas incineration was the least favorable for the environment [132].

Sewage sludge is mainly utilised in arable land and sanitary or secondary warehouses for building materials [133]. However, sewage sludge contains high concentrations of nitrogen, phosphorus, heavy metals, microplastics and polycyclic aromatic hydrocarbons. This can result in the accumulation of contaminated matter in the soil over time, which adversely affects the ecosystem. Various sludge removal techniques lead to substantial differences in environmental impact. Liu et al. conducted an LCA study on the environmental impacts of two WWTPs and one sludge incineration plant. Although sludge treatment can be improved, it can have adverse environmental impacts, such as increasing human and freshwater ecological toxicity and acidification potential [126]. Chen et al. concluded that combined municipal solid waste and sludge combustion was the best option [134]. Nevertheless, identifying solutions that effectively mitigate the risk of further environmental contamination via sludge is essential. Sludge disposal in landfills has been linked to ARG enrichment in soils, highlighting the requirement for environmentally sustainable disposal techniques [135].

#### 3.3.2. Environmental Impact Quotient

EIQ is a tool used to assess the environmental effect of pesticides (fungicides, herbicides and insecticides), considering factors such as toxicity and persistence [136]. Muhammetoglu et al. used this model to measure the environmental impact of the active ingredients in pesticides used to produce vegetables and fruit in Turkey’s Kumluca region. EIQ was found to be effective in establishing various strategies or programmes to combat agricultural pests [137]. Contrastingly, Kniss et al. have found that this program is not as effective as previously thought in evaluating the impact of herbicides on the environment [138]. Fartassi et al. used this technique in a study conducted in a semi-arid region of Morocco to evaluate the trade-offs of ecosystem services in irrigated farmland [139].

#### 3.3.3. Grey Water Footprint

Water footprint helps in analysing pollutants generated by human activities, according to their impact on freshwater resources. It quantifies the volume of freshwater necessary to assimilate and dilute a given quantity of pollutants to standard limits, considering specific background concentrations and environmental standards [140,141]. In a study conducted in China on the effectiveness of this technique, Feng et al. showed that it can provide valuable insights to help in selecting measures for environmental rehabilitation [140]. According to Li’s study, conducting a GWF evaluation improves water quality and contributes to sustainable development. Additionally, the study found that the absolute temporal variation in GWF efficiency indicates an upward trend [142]. Similar studies have been conducted on veterinary antibiotics, showing that <1% of the administered antibiotics reach surface waters, though some do accumulate in soil. However, the impact of human pharmaceuticals, which include antibiotics, on surface waters is more substantial than that of veterinary antibiotics [143,144].

Technological advances, innovation and a commitment to sustainable practices can ensure that wastewater treatment becomes a cornerstone of environmental protection, contributing to a healthier, more sustainable future.

#### 3.3.4. One Health Perspective on Environmental β-Lactamase Genes Dissemination

The high prevalence and diversity of β-lactamase-encoding genes observed in water, soil and sludge can be best explored through the One Health framework. This framework treats human, animal and environmental health as interconnected rather than as sectors that can be managed independently [145]. The framework acknowledges that resistant bacteria and their associated mobile genetic elements such as plasmids, transposons, and integrons spread across clinical, veterinary, and environmental compartments, forming an interconnected epidemiological system [146]. The transmission chain underlying this system shows how the β-lactamase genes examined in this review likely spread and emerge from human and animal populations into the environment. Agricultural runoff and manure application introduce resistant bacteria and antibiotic residues directly into soil and waterways. Meanwhile, inadequately treated municipal sewage, hospital effluents, and pharmaceutical discharge act as concentrated sources [147]. These reservoirs contaminate irrigation and drinking water, carrying resistant genes back towards humans and animals [148]. Furthermore, migratory wildlife extends the chain across geographic and political boundaries [149].

This transmission chain is precisely why fragmented, sector-specific interventions are considered insufficient under One Health. A coordinated response requires integrated surveillance across human, veterinary, and environmental matrices, ideally supported by molecular methods that track specific genes along this chain [150]. Within this framework, environmental management could efficiently cover areas such as agriculture, wastewater treatment, and pharmaceutical discharges [151]. As environmental reservoirs, particularly wastewater, function as an active link in the broader environmental-animal–human transmission chain, there is a need to extend the surveillance, stewardship and governance structures already applied in clinical and veterinary medicine to the environmental sector [152].

## 4. Limitations

This systematic review has several limitations that should be considered when interpreting the results. The literature search was restricted to articles published in English. While this approach ensured a focused and up-to-date dataset, it may have excluded relevant studies published in other languages. Considerable heterogeneity was observed across the included studies in terms of sampling strategies, environmental matrices, molecular detection approaches, and the spectrum of targeted β-lactamase genes. This variability limits the direct comparability of studies and prevents the quantitative synthesis of resistance gene prevalence. An additional limitation is the potential for publication bias, which may affect the interpretation of the results.

## 5. Conclusions

This systematic review emphasises that WWTPs are crucial hotspots for ARG accumulation, selection, and dissemination, particularly those encoding β-lactamases. Despite their role in decreasing microbial loads, conventional treatment processes are often insufficient in removing ARGs and resistant bacteria from effluents, leading to their release into receiving water bodies and broader environmental compartments. Among the β-lactamases analysed according to the selection criteria, *blaCTX-M*, *blaTEM*, and *blaOXA* gene families were reported with the highest prevalence, indicating their pervasive presence in the environment. Among the carbapenemase-encoding genes, *blaKPC* and *blaNDM* were the most frequently reported. *blaSHV* was also detected at a comparably high frequency even though it primarily functions as a broad-spectrum ESBL rather than a true carbapenemase. Additional genes associated with reduced carbapenem susceptibility, including *blaVIM*, *blaGES*, and *blaIMP*, were also widespread, further indicating the environmental dissemination of resistance determinants. AmpC-type β-lactamases, including *blaAmpC*, *blaCMY*, *blaCIT*, *blaDHA*, *blaFOX*, *blaACC*, *blaMOX*, and *blaACT*, were consistently identified, although at lower frequencies. A broader spectrum of additional gene families, including *blaPSE*, *blaSIM*, *blaVEB*, *blaPER*, and *blaCfxA*, had a low to moderate occurrence. *blaADC*, *blaZ*, *blaPOM*, *blaEC*, and *blaSRT* were detected only sporadically. The consistent detection of β-lactamase genes across diverse environmental matrices underscores the urgent need for integrated surveillance strategies and advanced molecular monitoring tools. Tracking specific β-lactamase variants can help identify emerging threats and clarify the environmental pathways that facilitate transmission to human and animal populations. Therefore, revising and upgrading wastewater treatment technologies, incorporating routine screening for priority resistance genes, and enforcing stricter discharge regulations are imperative. Collaboration between policymakers, environmental agencies, and researchers is necessary to address these challenges. Such collaboration must encompass the development of standardised methodologies, funding long-term monitoring programmes, and supporting international data-sharing platforms.

Future research should focus on expanding geographical representation, particularly in understudied regions, and combining molecular detection with phenotypic assessments to provide a more comprehensive understanding of resistance dynamics and implications for public health. The requirement to address AMR at the environmental level is becoming increasingly apparent. This is essential to safeguard public health and preserve the effectiveness of existing antimicrobial treatments. Studies targeting the persistence, expression, and horizontal transfer of β-lactamase genes will be particularly valuable for designing targeted mitigation strategies.

## Figures and Tables

**Figure 1:**
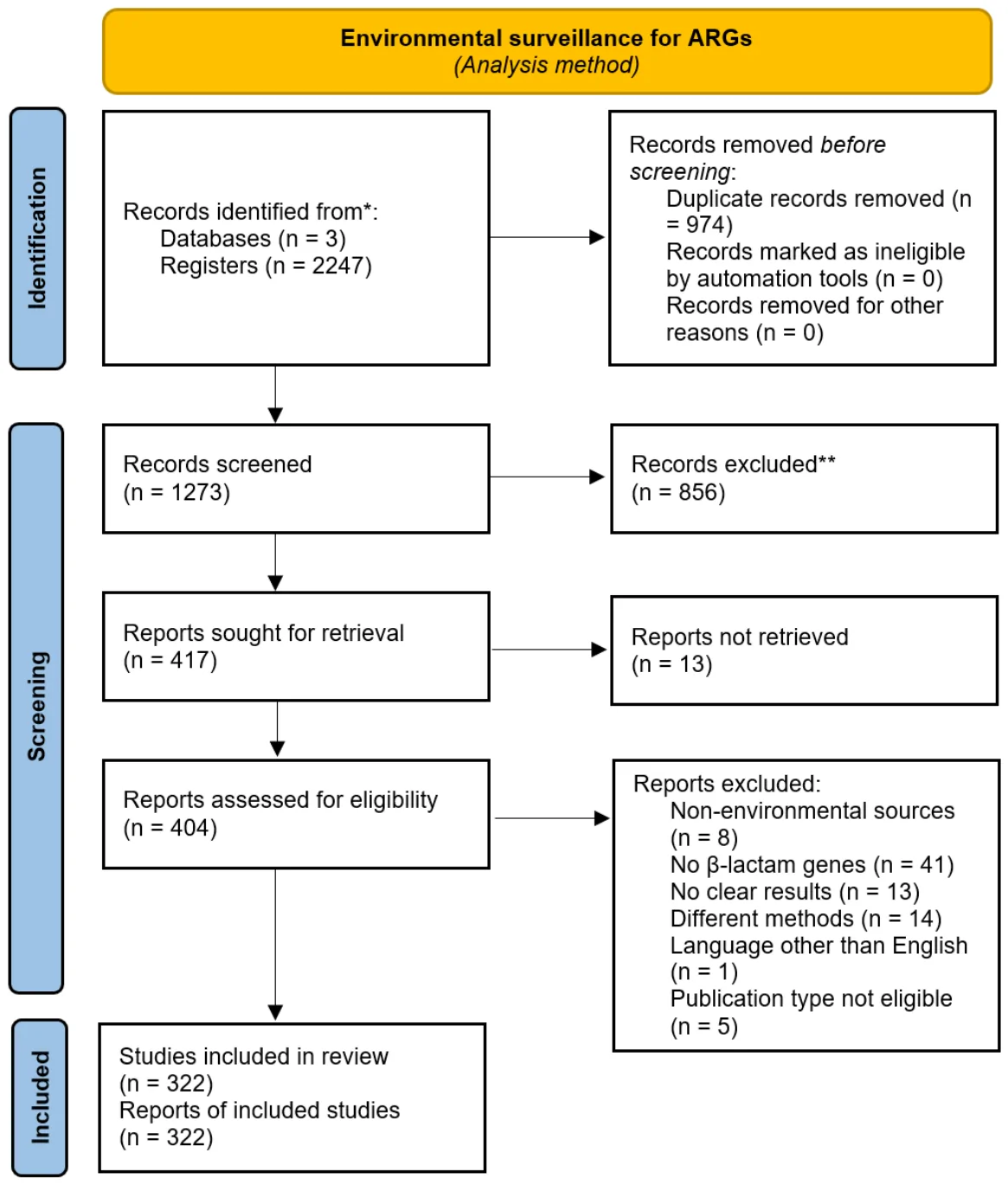
PRISMA flow diagram illustrating the process of selecting publications. * the number of records identified from all databases or register searched, ** no automation tools were used.

**Figure 2:**
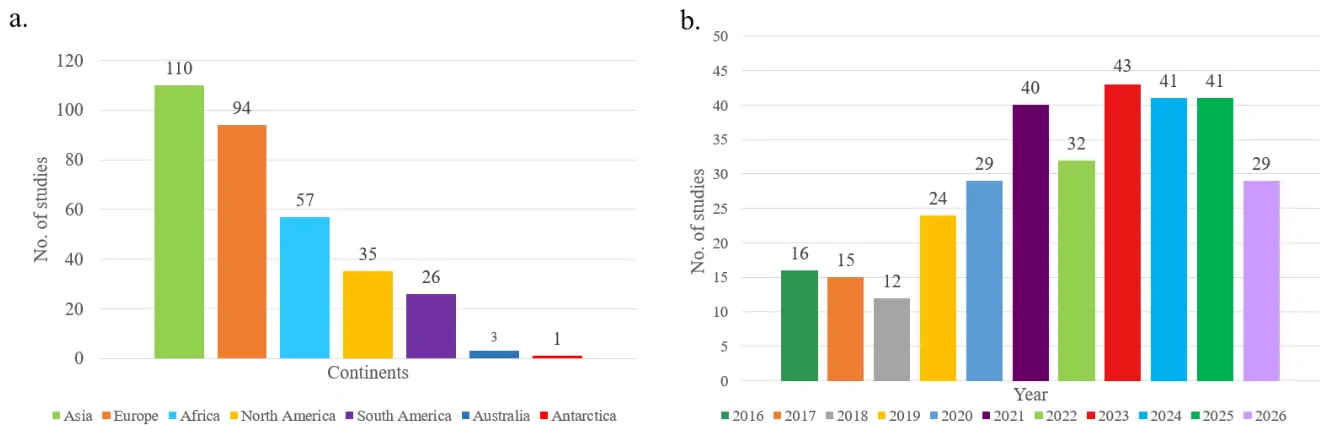
Distribution of the studies published and reviewed in the present study: (**a**) by continent; (**b**) by year.

**Figure 3:**
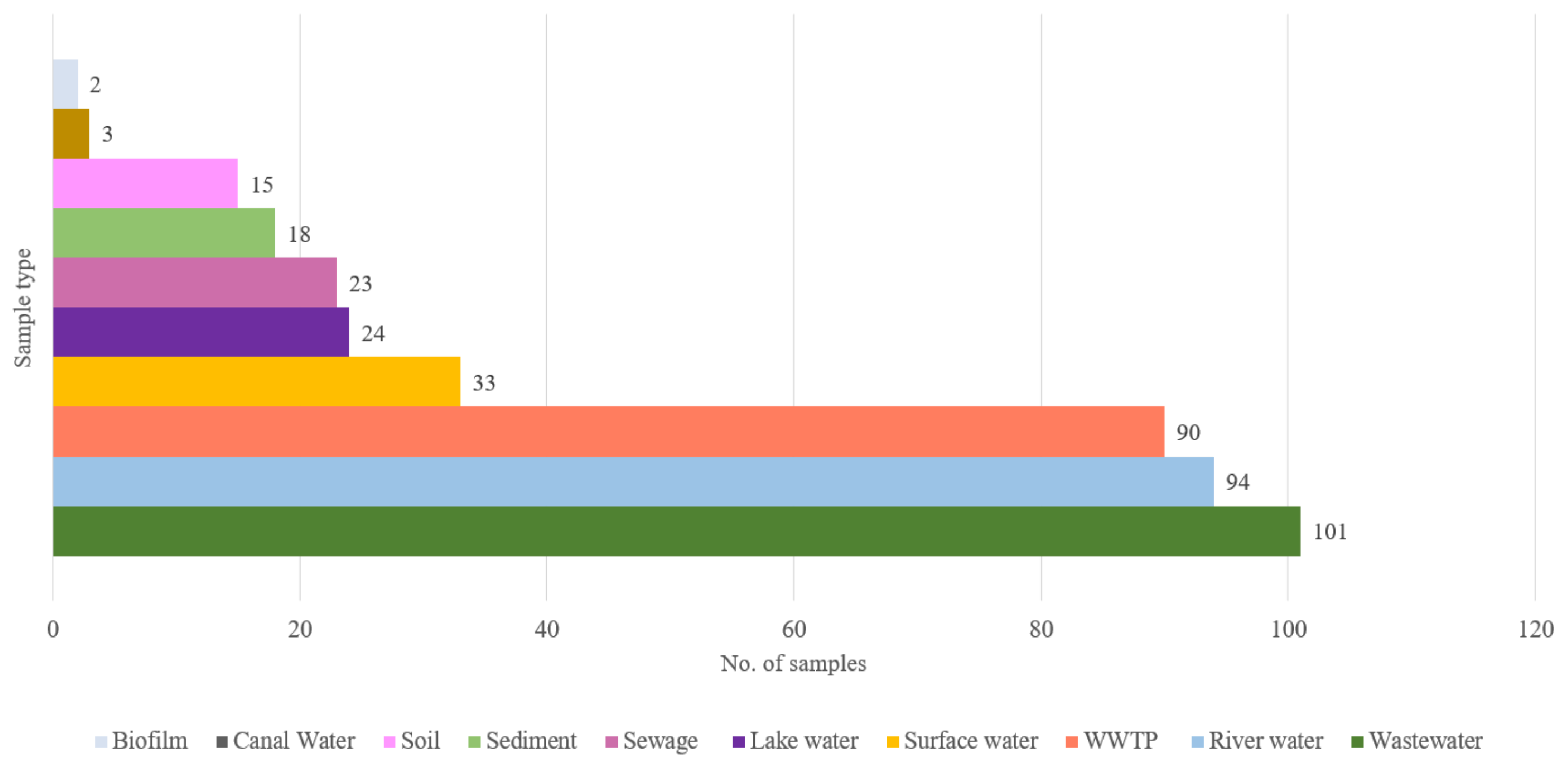
Sample types represented across the included studies.

**Figure 4:**
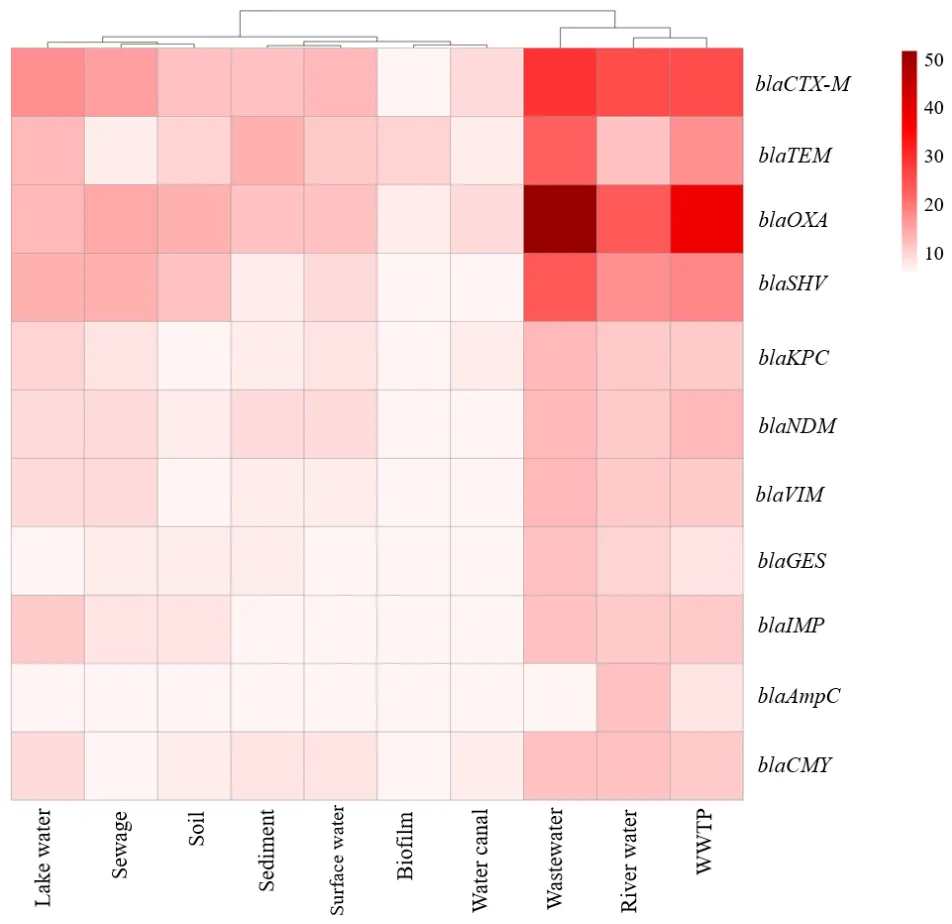
Frequency of ARG detection across environmental matrices.

**Figure 5:**
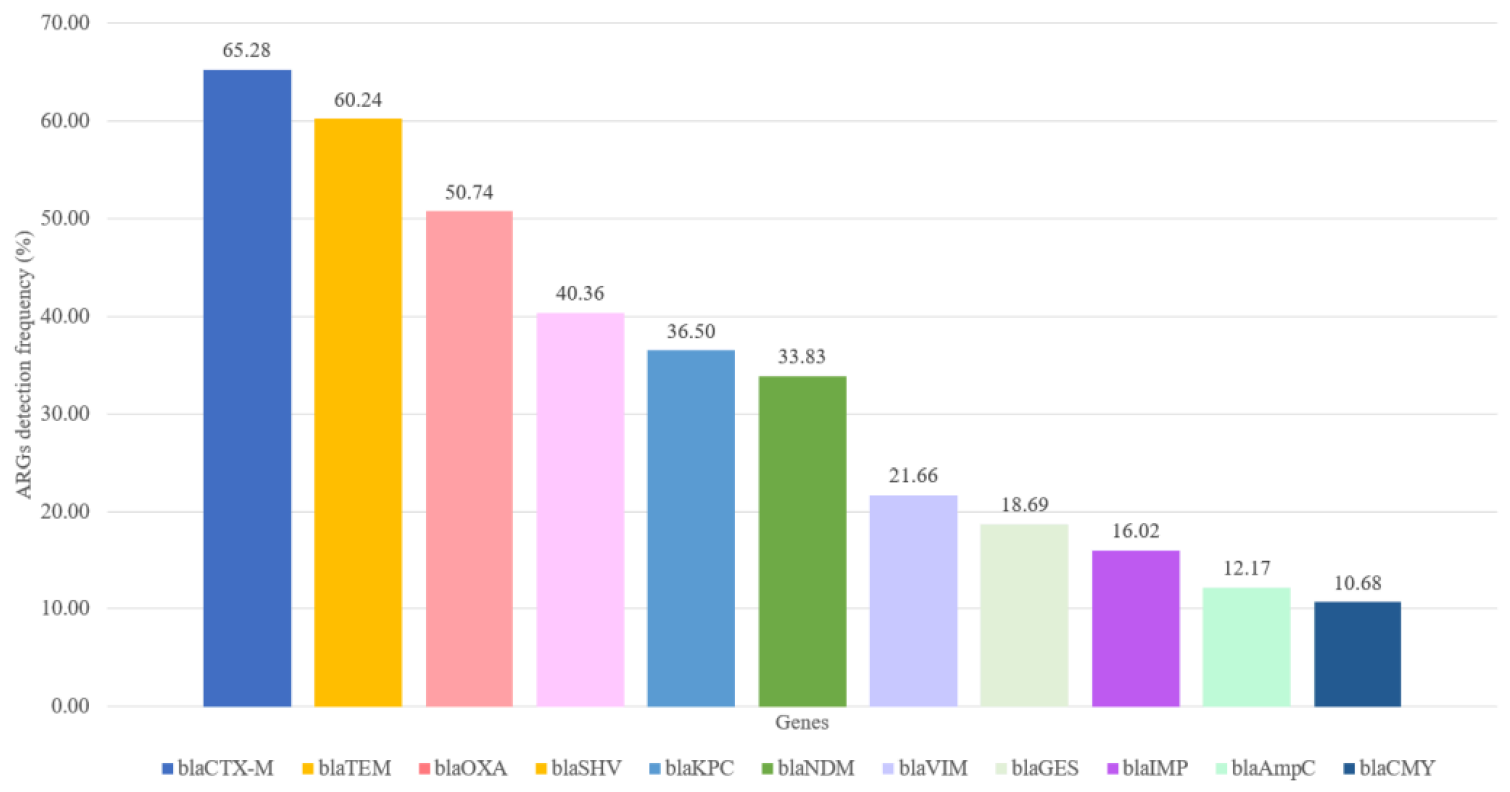
Frequency of detected antimicrobial-resistance genes in the analysed studies.

**Figure 6:**
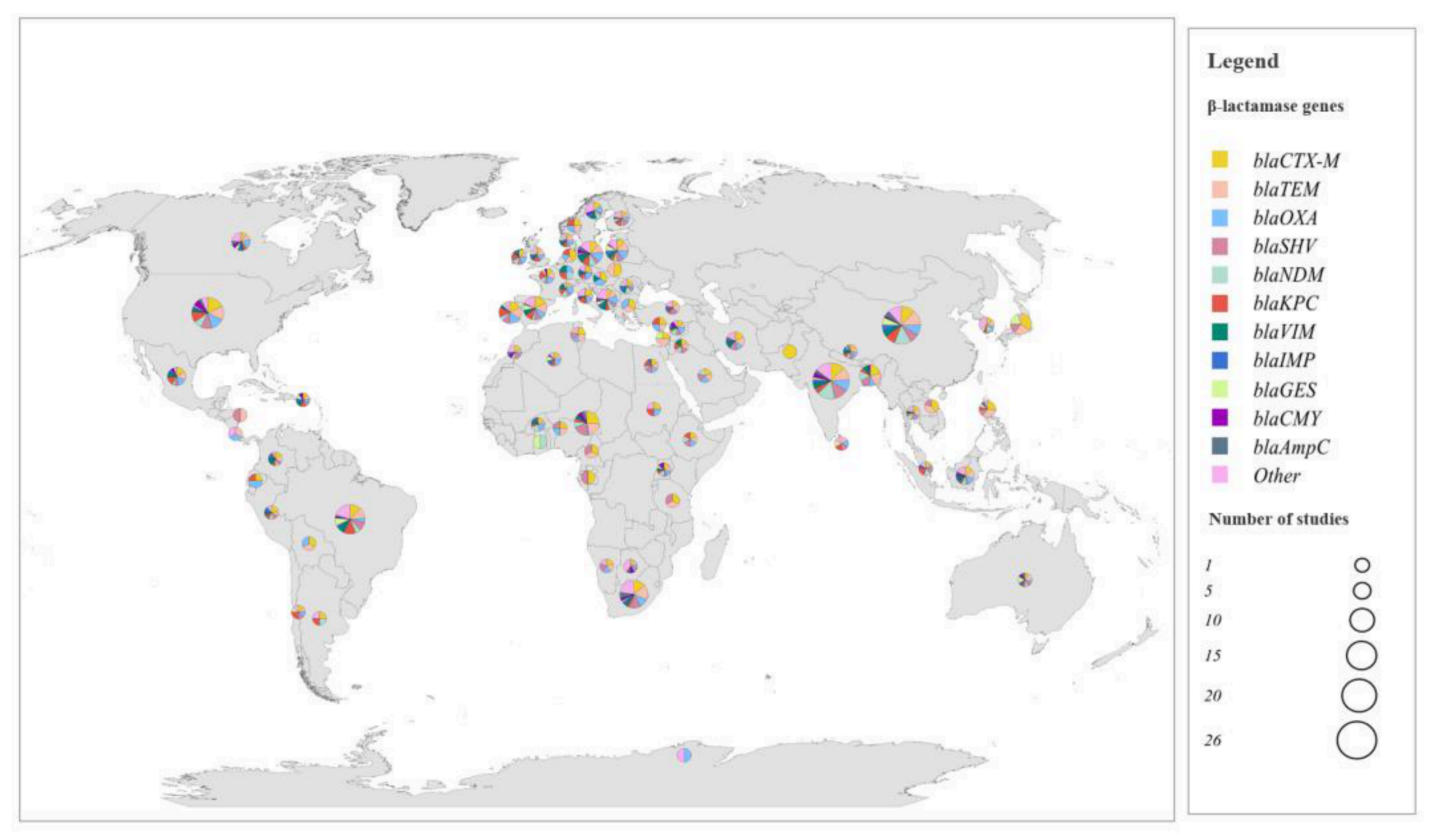
Geographical distribution of the reported ARGs and study coverage across countries.

**Table 1:** Search method.

Criteria	Query	No. of Results in PubMed	No. of Results in WOS	No. of Results in Scopus
#1	(“wastewater” OR “waste water” OR “sewage” OR “sewerage system” OR “wastewater treatment plant” OR “WWTP” OR “surface water” OR “groundwater” OR “drinking water” OR “river” OR “lake” OR “soil” OR “manure” OR “sludge” OR “biosolids”) NOT (review)	417,916	1,213,916	1,227,693
#2	(“beta-lactamase” OR “β-lactamase” OR “*bla* gene” OR “beta-lactam resistance” OR “β-lactam resistance” OR “beta-lactam resistant” OR “β-lactam resistant”) NOT (review)	16,554	19,337	29,384
#3	(“PCR” OR “RT-PCR” OR “qPCR” OR “dPCR” OR “whole genome sequencing” OR “ WGS” OR “next-generation sequencing” OR “NGS” OR “metagenomic” OR “culture-independent” OR “bacterial isolate” OR “culture-dependent” OR “environmental DNA” OR “eDNA”) NOT (review)	465,328	558,625	611,675
	#1 AND #2 AND #3 (“wastewater” OR “waste water” OR “sewage” OR “sewerage system” OR “wastewater treatment plant” OR “WWTP” OR “surface water” OR “groundwater” OR “drinking water” OR “river” OR “lake” OR “soil” OR “manure” OR “sludge” OR “biosolids”) AND (“beta-lactamase” OR “β-lactamase” OR “*bla* gene” OR “beta-lactam resistance” OR “β-lactam resistance” OR “beta-lactam resistant” OR “β-lactam resistant”) AND (“PCR” OR “RT-PCR” OR “qPCR” OR “dPCR” OR “whole genome sequencing” OR “ WGS” OR “next-generation sequencing” OR “NGS” OR “metagenomic” OR “culture-independent” OR “bacterial isolate” OR “culture-dependent” OR “environmental DNA” OR “eDNA”) NOT (review)	498	668	1081

**Table 2:** Wastewater removal technologies for ARBs and ARGs.

Bacterial Strains and ARGs	Implemented Methodology	Removal Efficiency	References
*Escherichia coli*, *Yersinia ruckeri*, *Enterobacter agglomerans*	Adsorption through magnetic graphene oxide (MGO)	high antibacterial efficiency	[104]
*Escherichia coli* (strain ATCC-19853) and *MS2* bacteriophage (ATCC-15767)	Natural coagulant (from seed extract of *Strychnos potatorum*)	reduction of 3.3–4.3 log for ^E. coli^ and 1.3 log for MS-2 bacteriophage	[105]
*Escherichia coli* and *Enterococcus faecium* carrying ARGs.	Ozone method	5.0 log reduction in bacterial cells and a 4.3–4.6 log reduction in ARGs.	[106]
	Free chlorine	3.8–5.6 log reduction in the bacterial numbers and a 0.8–2.8 log reduction in ARGs	
	Ultraviolet light irradiation	4.8–5.5 log reduction in bacterial cells, negligible reduction (0–1.0 log) of ARGs	
*Escherichia coli* K12	Solar water disinfection (SODIS)	the time needed to inactivate 99.99% of *E. coli* was reduced.	[108]
*Escherichia coli*	Photo-Fenton technique combined with H_2_O_2_/UV	the combined method was more effective at inactivating *E. coli* in seawater compared to UV treatment alone	[109]
*Escherichia coli*	Photocatalysis technology with Ce-TiO_2_ and B-TiO_2_	Ce-TiO_2_ had a 1.302 log (95.01%) reduction and B-TiO_2_ 6.426 log (99.00%) reduction in *E. coli*	[110]
*Escherichia coli*	Visible-light-driven plasmon-enhanced photocatalysis using Bi_2_MoO_6_/Ag–AgCl with Ag acting as an electron-transfer mediator and AgCl as catalytic active sites.	complete inactivation of ^E. coli^ (10^7^ CFU/mL) was achieved within 30 min under visible light irradiation.	[111]
23 ARGs; Wastewater bacterial community	Electrochemical disinfection under different current densities and reaction times	T treatment was less effective at eliminating ARGs, despite being more efficient at inactivating bacteria	[112]
Wastewater samples containing coliform bacteria	Electrochemical anodic oxidation using Ti/Sb–SnO_2_/PbO_2_ anode and stainless-steel cathode	Electrochemical disinfection studies showed that complete disinfection of one from the influent of the sequence batch reactor (WW1) and the other from the effluent of constructed wetland (WW2) was achieved within 30 and 6 min, respectively.	[113]
*Acinetobacter baumannii*	Metal-exchanged zeolites (natural zeolite; natural zeolite clinoptilolite enriched with silver (AgNZ); copper-exchange zeolite (CuNZ))	CuNZ showed slight antibacterial activity. AgNZ removed 90–100% of carbapenem-resistant bacteria during the 4 days of the experiment	[114]
Faecal coliforms (FC), total coliforms (TC) and somatic coliphages	Ultraviolet system	TC average reductions were 2.53, 0.87 and 0.44 uLog in winter, spring and summer. For FC, reductions were 2.03, 1.27 and 0.33 uLog in winter, spring and summer. For somatic coliphages, reductions were 1.22, 1.18 and 0.58 uLog in winter, spring and summer.	[115]
*Acinetobacter*, *Enterococcus*, *Escherichia coli*, *Pseudomonas aeruginosa*, *Staphylococcus aureus*	UV- and UV-LED-mediated disinfection	>99.9% of AMRs from wastewater were inactivated after 1–3 min of UV and 3 min of UV-LED treatment; ARGs were removed by 0.2–1.4 log10 for UV 254 nm and 0.1–1.3 log10 for UV 280 nm	[116]
*Clostridium perfringens*, *Escherichia coli*	Sequencing batch biofilter granular reactor (SBBGR) technology combined with sand filtration and solar wastewater source heat pump	SBBGR: 1.4 log_10_ reduction units for *C. perfringens*; sand filtration was 1.5 log10 units; 3 log_10_ units for *E. coli*	[117]

## Data Availability

Data is contained within the article or Supplementary Material.

## Supplementary Materials

The following supporting information can be downloaded at https://www.mdpi.com/article/10.3390/microorganisms14102204/s1. Table S1: Summary of the studies on ARG surveillance in various environmental samples. References [17,102,103,153,154,155,156,157,158,159,160,161,162,163,164,165,166,167,168,169,170,171,172,173,174,175,176,177,178,179,180,181,182,183,184,185,186,187,188,189,190,191,192,193,194,195,196,197,198,199,200,201,202,203,204,205,206,207,208,209,210,211,212,213,214,215,216,217,218,219,220,221,222,223,224,225,226,227,228,229,230,231,232,233,234,235,236,237,238,239,240,241,242,243,244,245,246,247,248,249,250,251,252,253,254,255,256,257,258,259,260,261,262,263,264,265,266,267,268,269,270,271,272,273,274,275,276,277,278,279,280,281,282,283,284,285,286,287,288,289,290,291,292,293,294,295,296,297,298,299,300,301,302,303,304,305,306,307,308,309,310,311,312,313,314,315,316,317,318,319,320,321,322,323,324,325,326,327,328,329,330,331,332,333,334,335,336,337,338,339,340,341,342,343,344,345,346,347,348,349,350,351,352,353,354,355,356,357,358,359,360,361,362,363,364,365,366,367,368,369,370,371,372,373,374,375,376,377,378,379,380,381,382,383,384,385,386,387,388,389,390,391,392,393,394,395,396,397,398,399,400,401,402,403,404,405,406,407,408,409,410,411,412,413,414,415,416,417,418,419,420,421,422,423,424,425,426,427,428,429,430,431,432,433,434,435,436,437,438,439,440,441,442,443,444,445,446,447,448,449,450,451,452,453,454,455,456,457,458,459,460,461,462,463,464,465,466,467,468,469,470,471] are cited in Table S1.
