# Supplementary material for: Distribution of β-Lactamase Resistance Genes in the Environment: A Systematic Review
Source: Microorganisms. 2026 Oct 1;14(10):2204. doi: 10.3390/microorganisms14102204 (PMC13708008; doi:10.3390/microorganisms14102204)
Supplement: Supplementary file 1 [file microorganisms-14-02204-s001.zip › microorganisms-4561010-supplementary.pdf]

Supplementary Table S1. Summary of the studies on ARG surveillance in various environmental samples

	Surveillance Period	Sample type	Country	Target ARGs	Methods	Results	References
1.	October 2023 – December 2023	Wastewater hospitals, tertiary care hospitals 36 samples	Germany	*blaNDM*, *blaKPC*, *blaIMP*, *blaVIM*, *blaOXA*-23-like, *blaOXA-48*-like, and *blaOXA*-58-like	RT-qPCR, dPCR	The following values were obtained through qPCR: 10.57 ± 0.20 log GC/L for *blaVIM* at the TCH4 site; 0.22 ± 1.27 log GC/L for *blaOXA-48*-like at the TCH6 site and up to 6.96 ± 0.72 log GC/L for *blaIMP* at the TCH2 site. ExDNA associated with *blaKPC* was not present in samples from sites TCH1 and TCH2. *blaNDM* was not present in location TCH4. The dPCR analysis revealed that all target genes were present in all samples. The variation in detection ranged from 1.80 log units for *blaOXA-48*-like to 4.92 log units for *blaKPC*. qPCR often reported lower values.	[153]
2.	*N.R.*	Wastewater samples	Croatia: Zagreb	*blaKPC*, *blaOXA-48*, *blaNDM*, *blaVIM* and *blaIMP*, *mrc*-1*,* *mrc*-2*,* *mrc*-3, *mrc*-4*, mcr*-5	Culture-based method/ Multiplex PCR/ Ion Torrent PGM sequencing	In *K. pneumoniae,* the most prevalent were *blaOXA-48* and *blaNDM*-1, whereas *blaKPC*-2 and *blaNDM*-1 were mainly detected in *K. oxytoca, E. cloacae cplx.* and *Citrobacter spp*. *blaVIM*-1 and was mostly found in *K. oxytoca*, *Citrobacter*, *Kluyvera* and *Roultella*. *blaIMP*-13 was detected in *K. michiganensis*. Six *Klebsiella* isolates had ARGs such as: *blaSHV*-1, *blaOKP*-B, *blaOXY*-1-7, *fos*A and *oqx*A/B. Several acquired carbapenemase genes (*blaNDM*-1, *blaKPC*-2, and *blaOXA-48*) and ESBL genes *blaSHV*-12, *blaSHV*-98, *blaOXA*-1, *blaOXA*-9, *blaC*-15 and *blaC*-3 were also present in sequenced *Klebsiella spp.* The genes found in 3 E. cloacae cplx. The strains were: *blaKPC*-2, *blaOXA*-1, *blaOXA*-256, and *blaOXA*-10, *aac*(6′)-lb-cr, *aac*(3)-I, *aph*(6)-Id, and *aph*(3′')-lb, *aar*-3, *cat*B3.	[154]

						The following genes were identified in *E. coli* and other Enterobacterales strains: *blaNDM*-1, *blaKPC*-2, *blaVIM*-1, *blaOXA-48*, *blaTEM*-1, *blaOXA*-type (OXA-1 or OXA-10), *blaSHV*-12, *aac*(6′)-lb-cr, *aph*(3)-l and *aph*(6)-ld, *qnr*B9 or *aac*(6′)-lb-cr, *sul1*, *cat*A1, *dfr*A1, *blaOXA-48*. In Klebsiella, the following ARGs were present: *blaKPC*-2, *blaNDM*-1, *blaOXA-48*, *blaSHV*-1, *blaOKP*-B, *blaOXY*-1-7, *fos*A, *oqx*A/B, *blaOKP*-B, *fos*A, *blaSHV*-12, *blaSHV*-98, *blaOXA*-1, *blaOXA*-9, *blaC*-15, *blaC*-3, *qnr*B, *qnr*S and *aac*(6′)-lb-cr), *aac*(6′)-lb-cr, *aac*(3)-I, *aph*(3)-I and *aad*A1, *sul1* and *sul2*, *dfra*A14), *mph*A, *mph*E and *msr*E, *tet*A, *catB3* and *mcr*-4.3	
3.	January – February 2023	Drinking water, hospital effluents<br><br>2 drinking water, 12 hospital effluents samples	Benin	*blaGES*<br><br>*blaTEM, blaSHV, blaOXA*-1, *blaCTXM*-1, *blaCTXM-9, blaCTXM*-15*, blaKPC, blaNDM, blaIMP, blaVIM,* and *blaOXA-48*	Culture-based method/ Standard PCR	The most commonly identified ARG's were: *blaTEM* (37.50%), *blaOXA*-1 (19.44%), and *blaSHV* (11.11%). A smaller number of isolates carried *blaCTXM*-1/*blaCTXM*-15 (5.55%) and *blaCTXM*-9.	[155]
4.	October – December 2021	Hospital and municipal wastewater<br><br>3 wastewater samples, 3 hospital samples	Saudi Arabia	*blaCTX-M*, *blaSHV*, *blaTEM*, *blaKPC*, *blaIMP*, *blaVIM*, *blaOXA-48*, *blaNDM*-1	Culture-based method/ Multiplex PCR	Eight out of 11 isolates carried the following ESBL genes: *blaTEM* gene was found in 2 strains (*Pseudomonas* and *Enterobacter* species); the *blaSHV* gene was identified in 2 strains of *Citrobacter* and *Proteus* sp. *blaCTX-M* gene was present in 3 strains (*Acinetobacter* sp.). One *E. coli* isolate harboured *blaTEM* and *blaSHV*. *blaOXA-48* and *blaNDM*-1 were the most frequently found carbapenemase encoding genes (3/9 carbapenemase-producing isolates). Two of the *Klebsiella* species had the *blaOXA-48* gene. Additionally, *blaNDM*-1 was found in one isolate of *Acinetobacter* spp).	[17]

5.	20 April 2021, 24 August 2021, 16 November 2021, 3 February 2022	Hospital wastewater	Poland	*blaSHV*, *blaTEM*, *blaOXA*, *blaCTX-M*-1-group, *blaCTX-M*-2-group, *blaCTX-M*-9-group and *blaCTX-M*-8/25-group	Culture-based method/ qPCR	The following genes were detected: *blaSHV*, *blaTEM*, *blaOXA*, *blaCTX-M*-1-group, *blaCTX-M*-2-group, *blaCTX-M*-9-group and *blaCTX-M*-8/25-group.	[156]
6.	August 2019 – January 2020	Seawater, sewage, river, estuary, estuarine lagoon, lake, drinking water treatment plant influent, hospital sewage, wastewater treatment plant influent, nursing home, airport<br>118 water samples and 36 sewage samples	Ireland: Galway City, Galway County, Fingal and Cork County	*blaCTX-M*-group-1, *blaCTX-M*-group-2, *blaCTX-M*-group-9, *blaOXA*, *blaKPC*, *blaNDM*, *blaVIM*, *blaIMP*	Culture-based method/ RT-PCR/ Illumina NovaSeq 6000, Illumina MiSeq sequencing	The following ARGs were detected in sewage and water samples: *blaOXA-48* (n = 18), *blaNDM* (n = 14), *blaKPC* (n = 4) and *blaOXA-48*4 (n = 1).<br>The most prevalent ESBL gene identified was *blaCTX-M-15* (n = 63), followed by 17 isolates that harbored *blaCTX-M*-27.	[157]
7.	February, June, and September 2019	Untreated and treated wastewater, river water	Poland: Warmia and Mazury regions	*blaIMP*, *blaIMP*-1, *blaVIM*, *blaVIM*-2, *blaNDM*, *blaOXA*-23-like, *blaOXA*-24-like,	Culture-based method/ PCR, RT-qPCR/ Illumina	Standard PCR showed that *Acinetobacter* spp. isolated from wastewater samples harboured 9 CRGs (*blaNDM*, *blaIMP*, *blaVIM*, *blaOXA*-23, *blaOXA*-24, *blaOXA*-51, *blaOXA*-58, *blaIMP*-1 and *blaVIM*-2) as well as *int*I1, *int*I2 and *int*I3.	[158]

				blaOXA-51-like, *blaOXA*-58-like	NovaSeq 6000 sequencing	The *blaOXA*-51 gene was identified as the most prevalent resistance gene, whereas *blaOXA*-23 was detected less frequently.	
8.	November 2019 – January 2020	Faecal sludge treatment plants (fstps)<br>108 samples	Bangladesh: Rohingya camps, Cox's Bazar	*blaCTX-M*-1, *blaCTX-M*-2, *blaCTX-M*-8, *blaCTX-M*-9, *blaCTX-M*-15, *blaCTX-M*-25, *blaSHV*, *blaTEM*	Culture-based method/ Standard PCR	Among the 296 isolates examined, 180 were phenotypically positive for ESBL.<br>A total of 93% (168/180) of the isolates were positive for the *blaCTX−M−1* gene followed by 58% (105/180) for the *blaCTX*−M−15 gene, 42% (75/180) for the *blaTEM* gene, 2% (3/180) for the *blaSHV* gene and 0.6% (1/180) for the *blaCTX*−M−9 gene. Contrastingly, the *blaCTX*−M−2, *blaCTX*−M−8 and *blaCTX*−M−25 genes were not detected in any of the isolates. Among the *blaCTX*−M−1, *blaCTX*−M−15, *blaTEM* and *blaSHV* genes, a maximum of three genes (*blaCTX*−M−1, *blaCTX*−M−15, and *blaTEM*) were identified in 19% (34/180) of the isolates. Furthermore, the presence of *blaCTX*−M−1 and *blaCTX*−M−15 were found to co-exist in 38% (69/180) and *blaCTX*−M−1 and *blaTEM* in 17% (31/180) of the isolates. However, no isolate was identified to carry these four genes together. The gene percentage of the samples from the inlet was as follows: 93% *blaCTX*−M−1 (70/75), 57% *blaCTX*−M−15 (43/75), 49% *blaTEM* (37/75), and 1% *blaSHV* (1/75) of the isolates. However, in the outlet, it was 93% (98/105), 59% (62/105), 36% (38/105), and 2% (2/105), respectively for the same genes.	[102]
9.	February, June, September 2019	Wastewater (WWTP), river<br>15 samples	Poland: Olsztyn	*blaCTX*, *blaSHV*, *blaTEM*, *blaOXA*, *blaKPC*, *blaGES*, *blaNDM*, *blaIMP*, *blaOXA-48*, *mdt*K	Culture-based method/ Standard PCR	90.7% (245 out of 270) of the environmental isolates had beta-lactam resistance genes.<br>The *blaGES* gene was identified in 8.2% of all isolates, making it the most prevalent carbapenemase gene.<br>The *blaVIM* gene was identified in 6.0% of all isolates. The prevalence of *blaKPC*, *blaIMP*, and *blaOXA-48* genes was similar (2.2%, 1.9% and 2.2% of all isolates, respectively). However,	[159]

						blaKPC and *blaIMP* were not detected in any of the isolates from river water. The *blaOXA-48* gene was exclusively detected in *K. pneumoniae* isolates. The isolates did not contain the *blaNDM* gene, regardless of the season and sampling site.<br><br>The prevalent genes identified in river water samples were *int*I1 and *blaTEM*. *blaSHV* (25.4%) and *blaOXA* (9.9%) showed higher prevalence in *Klebsiella pneumoniae* (53.8% and 17.3%, respectively) than in *K. non-pneumoniae* (4.5% and 3.7%) strains. The *blaCTX* gene was identified as the least abundant β-lactamase gene (1.3%).<br><br>The *acr*AB gene was identified in 92.2% of all isolates. The *tol*C gene was detected in 74.6% of isolates from wastewater but less frequently detected in river water (53.1%) and in *K. non-pneumoniae* isolates (58.4%). The *mdt*K efflux pump gene was present in only 8/270 of the tested strains (2.9%).	
10.	July 2018	Municipal effluent discharges (WWTP), river water<br><br>24 water samples	Sweden: Uppsala, Stockholm, Eskilstuna and Västerås	*aad*A-1, *aad*A5–1, *aph*(2')-Id, *aad*D, *aph*, *aad*A9-1, *aad*A-2, *aph*A1/7, *aac*(6')I1, *aac*3-VI, *aac*C2, *aad*A1, *aad*E, *str*, *str*A, *str*B, *spe*A, *spc*N-1, *acc*, *aac*C4, *aac*A/*aph*D, *aac(*6')-Iy, *aac*(6')-II, *aph*A3-1, *aac*(6')-Ib-1, *aad*A2-1<br><br>*cml*A-1, *cat*B3, *cat*B8, *yid*Y/*mdt*L, *mdt*L, *cml*A-2, *cml*A-3, *cmx*A, *cat*A1, *rar*D-1, *rar*D-2, *cml*A-4<br><br>*amp*C-2, *blaMOX*/*bla-CMY*, *blaOCH*, *blaPAO*, *blaSHV*-1, *blaVEB*,	RT-qPCR	A total of 167 out of 296 genes were identified in the examined samples.<br><br>On average, the total abundance of resistance genes was the highest in Västerås (1.1<br><br>10−1), followed by Uppsala (1.7<br><br>10−2), Stockholm (5.5<br><br>10−3) and Eskilstuna (5.0<br><br>10−3).<br><br>Among the examined samples, those showing the highest average relative abundance of ARGs were those against β-lactams and sulphonamides. Contrastingly, vancomycin resistance genes were exclusively detected in samples from Uppsala. The study identified the presence of resistance genes linked to aminoglycosides, tetracyclines, amphenicol, trimethoprim, florfenicol,	[160]

				blaOKP, *blaROB*, *blaOXY*, *blaPSE*, *blaOXA10*, *cph*A-1, *cph*A-2, *blaL1*, *cfx*A, *cep*A, *bla-CMY*2,blaAmpC/*blaDHA*, *fox*5,blaAmpC-3, *blaOXA1*/*blaOXA30*, *blaCTX-M*-1, *blaCTX-M*-2, *blaCTX-M*-3, *blaCTX-M*-4, *blaGES*, *blaSFO*, *blaTLA*, *blaZ*, *blaVIM*, *blaIMP*-1, *pbp*5, *pbp*, *mec*A, *blaCTX-M*-5, *blaSHV*-2, *blaTEM*, *blaCTX-M*-6, *pen*A, *pbp*2x, *blaPER*, *blaIMP*-2, *blaKPC*-1,blaAmpC-4,blaAmpC-5, *blaOXA58*, *blaKP*-3, *blaCTX-M*-8, *blaSHV*-3, *blaCTX-M*-7, *blaIMP*-3, *pbp*2b, *blaNDM*, *blaKP*-2, *bla*-SME, *cfi*A, *amp*-1, *bla-CMY*-1<br><br>*sul*4, *sul1*-1, *sul2*-1, *sul3*, *sul1*-2, *fol*P-1, *fol*A-1<br><br>*dfr*A1-1, *dfr*A1-2<br><br>*pmr*A, *ceo*A, *acr*B-1, *acr*B-2, *acr*F, adeA, bexA, sat4, cmeA, cmr, *acr*A-1, *emr*D-1, *mdt*A, *mdet*l1, *mdt*E, *mdt*G-1, *yce*L/*mdt*H, *mdt*H-1, *mex*A, *mex*D, *qac*H, *emr*B/*qac*A-1, *sde*B-1, *mtr*E, *mtr*D-1, *opr*D, *opr*J, *ttg*A, *mep*A, *mex*E, *mex*F, *mtr*C, *acr*A-2, *emr*D-2, *qac*A/*qac*B,		vancomycin, quinolone and β-lactams, MLSB, and MDR, in the studied waters.	

				cfr, *tol*C-1, *emr*B/*qac*A-2, *qac*, *mdt*F, *mex*B<br><br>*flo*R-1, *flo*R-2<br><br>*erm*D, *erm*K, *erm*35, *erm*F, *ere*A, *ere*B, *lnu*B-1, *lnu*B-2, *vat*D, *vat*E-1, *lmr*A-1, *erm*36, *erm*34, *erm*B, *erm*T-1, *msr*C-1, *mat*A/*mel*, *mph*A-1, *mph*B, *mph*A-2, *vgb*-1, *vgb*-2, *mef*A, *msr*A-1, *msr*C-2, *erm*X, *vat*B-1, *vat*C-1, *vga*A-1, *vga*B-1, *pnc*A, *erm*T-2, *lnu*A-1, *lmr*A-2, *vat*E-2, *vga*B-2, *vga*A-2, *vat*B-2, *vat*C-2, *vgb*B, *erm*Y, *lnu*A-2, *mph*, *erm*A, *erm*C, *msr*A-2, *lnu*C, *erm*A/*erm*TR, *ole*C, *car*B, *pik*R1, *pik*R2<br><br>*tet*A-1, *tet*36-1, *tet*32, *tet*U-1, *tet*D-1, *tet*O_1, *tet*A/B-1, *tet*C-1, *tet*A-2, *tet*G-1, *tet*K, *tet*Q, *tet*H, *tet*G-2, *tet*A/B-2, *tet*W, *tet*O-2, *tet*M-1, *tet*L-1, *tet*X, *tet*C-2, *tet*S, *tet*V, *tet*J, *tet*38, *tet*E, *tet*PB-1, *tet*PB-2, *tet*T, *tet*36-2, *tet*37, *tet*35, *tet*34, *tet*M-2, *tet*C-3, *tet*D-2,<br><br>*qnr*A, *qnr*B<br><br>*van*C-1, *van*C-2, *van*TC-1, *van*A, *van*B-1, *van*TE, *van*D, *van*HD, *van*HB, *van*RA-1, *van*SA, *van*WG, *van*WB, *van*XA, *van*XD, *van*XB, *van*C1,			

				*van*C2/*van*C3, vanG, *van*RB, *van*RC, *van*RC4, *van*RD, *van*SB, *van*SC-1, *van*SE, *van*TG, *van*YB, *van*YD-1			
11.	October – November 2017	Hospital wastewater	South Africa: Eastern Cape Province	*blaCTX-M* (inclusive of groups 1, 2, and 9), *blaTEM*, *blaSHV*, *blaGES*, *blaIMP*, *blaKPC*, *blaVIM*, *blaOXA*-1-like, *blaPER*, *blaOXA-48*-like, *blaVEB*, *blaACC*, *blaEBC*, *blaFOX*, *blaCIT*, *blaDHA*, *blaMOX*, *aad*A, *cat*I, *cat*II, *str*A, *sul*I, *sul*II, *tet*A, *tet*B, *tet*C, *tet*D, *tet*K, *tet*M.	Culture-based method/ Standard PCR	64.5% (20/31) of the isolates carried the β-lactamase genes.<br><br>Non-β-lactam genes were identified in 87.1% (27/31) of the isolates.<br><br>ESBLs were present in 58.1% (18/31) of the isolates.<br><br>The *blaCTX-M* group (including groups 1, 2, and 3) was the predominant gene being present in 51.6% (16/31) of the isolates. *blaTEM* gene was identified in 32.3% (10/31) of the isolates. Other ESBL genes such as *blaSHV* (12.9% (4/31)), *blaPER* (6.5% (2/31)) and *blaVEB* (3.2% (1/31)) were identified. Carbapenemase genes such as *blaOXA*-1-like (19.4% (6/31)), *blaOXA-48*-like, *blaVIM* (15.4% (2/13)) and *blaIMP* (7.7% (1/13)) were also present. *blaCIT* gene was the most prevalent among pAmpCs, with 12.9% (4/31) of the isolates carrying it, followed by *blaFOX* (7% (3/31)). Others were *blaEBC* (6.5% (2/31)) and *blaDHA* (3.2% (1/31)). The presence of the *Cat*II (79.2% (19/24)), a non-β-lactam resistance gene, was also detected, followed by the *tet*A gene (46.7% (14/30)). The non-β-lactam encoding genes *sul*I and *sul*II (35.5% (11/31)), *tet*B (23.3% (7/30)), *aad*A (12.9% (4/31)), *tet*C (10% (3/30)), and *tet*D (3.3% (1/30)) were reported as well.	[161]
12.	April 2015 – August 2017	Hospital sewage water<br><br>20 samples	Sweden: Göteborg	*blaNDM*, *blaOXA-48*-like (including e.g. *blaOXA-48*, *blaOXA*-181, *blaOXA*-244), *blaKPC*, *blaIMP*, *blaVIM*, *blaCT*	qPCR/<br><br>Illumina MiSeq platform, PacBio	*blaOXA-48*-like gene was found in 19 out of 20 analysed sewage samples, *blaCTX-M* was present in all samples, *blaNDM* was detected in only one sample, and *blaVIM* and *blaIMP* were found in two samples. *blaKPC* was not detected.	[162]

				X−M (group 1 including e.g. *blaCTX*−M-15)	platform, MinION sequencing		
13.	November 2018 – July 2019	Hospital, nursing homes, airports, drinking water treatment plant (DWTP), storm water overflows, raw sewage discharges, primary and/or secondary wastewater treatment discharges, seawaters, rivers, lakes, estuaries, and untreated water<br><br>72 Seawater samples<br><br>24 Rivers samples<br><br>9 Lake samples	Ireland: Galway city, Galway County, Fingal, Cork County	*blaCTX-M*−group 1, *blaCTX-M*−group 2, *blaCTX-M*−group 9<br><br>*blaVIM*, *blaIMP*, *blaOXA-48*, *blaKPC*, *blaNDM*	Culture-based method/<br><br>RT-PCR/ Illumina NovaSeq 6000 sequencing	A total of 105 isolates were selected for real-time PCR, of which 77 were obtained from water samples and 22 from sewage water. Of all the ESBL variants detected, the most common was *blaCTX-M*−group 1 (n = 83) followed by *blaCTX-M*−group 9 (n = 16). Six isolates, confirmed phenotypically as ESBL producers, tested negative for the *blaCTX-M* genes. 2 out of 5 of considered 'cold spot' locations tested positive for the detection of one or more isolates carrying *blaCTX-M* genes. *blaOXA-48*, *blaNDM*, *blaKPC* and *blaIMP* were identified in 3 seawater isolates, 4 hospital sewage isolates, 2 WWTP influent isolates, 1 estuary and 1 lake water isolate. Of the 66 isolates that were sequenced, the most frequently detected beta-lactamase gene was *blaCTX-M* (n = 48) which could be further divided into the following subtypes *blaCTX-M*−15 (n = 34), *blaCTX-M*−14 (n = 7), *blaCTX-M*−27 (n = 4), *blaCTX-M*−1 (n = 1), *blaCTX-M*−24 (n = 1) and *blaCTX-M*−55 (n = 1). The other identified genes were: *blaTEM*-1 (n = 31), *blaOXA*-1 (n = 19) and *blaSHV* variants (n = 16). The *blaSHV* genes included *blaSHV*-11 (n = 5), *blaSHV*-1 (n = 5), *blaSHV*-76 (n = 3), *blaSHV*-60 (n = 1), *blaSHV*-27 (n = 1) and *blaSHV*-62-like (n = 1).	[163]

		27 Estuaries samples<br>37 Hospitals samples<br>20 Nursing homes samples<br>3 Airports samples<br>14 WWTP samples<br>9 DWTP samples					
14.	October 2011 – December 2012	River water<br>51 samples	Germany: Lahn River Nievern, Obernhof	*blaCTX-M*	RT-qPCR	*blaCTX-M* genes were present in the surface water samples taken from the Lahn River. Temporal variations and hydro-meteorological factors caused alterations in their abundances. *blaCTX-M* levels have been observed to rise following precipitation events.	[164]
15.	Spring and late Autumn in 2015 and 2016.	Wastewater (WTTP)	Ireland	*blaGES*, *blaGIM*, *blaIMI*, *blaIMP*, *blaKPC*, *blaNDM*, *blaOXA*-23, *blaOXA*-40, *blaOXA-48*, *blaOXA*-51, *blaOXA*-58, *blaVIM*<br>*blaTEM*, *blaSHV*, *blaCTX-M* - group genes 1, 2, 8, 9, and 25<br>*mcr*-1, *mcr*-2, *mcr*-3, *mcr*-4, *mcr*-5	Culture-based method/ Multiplex PCR	The *blaTEM*, *blaSHV*-12 and *blaCTX-M*-1 genes were identified in 62 isolates using multiplex PCR. Most of the ESBL producers were *E. coli*: 46 carried *blaTEM*, 2 carried *blaSHV*-12, one carried *blaCTX-M*-1, and 2 carried *blaCTX-M*-15. Five of the seven *Klebsiella spp*. isolates carried the *blaSHV* gene, two had the *blaCTX-M-15* gene, and one isolate had the *blaCTX-M-15* gene. The gene *blaCTX-M-15* was found in one *Enterobacter* spp. The *mcr genes* have not been identified in any isolates.	[165]

16.	*N.R.*	Surface water, drinking water, river, ponds	Turkey: Kayseri	*bla*$_{SHV}$, *blaTEM*, *blaCTX-M*, *blaNDM*, *blaOXA-48*, *blaKPC*, *mcr*-1, *mcr*-2, *mcr*-3, *mcr*-4, *mcr*-5, *mcr*-6, *mcr*-7, *mcr*-8.	Standard PCR/RT-qPCR	Colistin resistance genes were identified in 80% (12/15) of the isolates, with the following distribution: *mcr-1*/2/6 (20%, n=4), *mcr-3/7* (13%, n=3), and *mcr-5* (40%, n=2). Additionally, ESBL-encoding genes *blaSHV* and *blaTEM* were each detected in 6.6% of the samples. Conversely, *blaNDM*, *blaOXA-48*, *blaKPC*, and *blaCTX-M* were not identified in any of the isolates.	[166]
17.	September – October 2022	2 river water samples<br>5 water canal samples 18 swimming lake samples	Germany: Lower Saxony	*blaVIM, blaKPC, blaNDM, blaOXA-48, blaOXA-23, blaIMP, blaGIM, blaIMI, blaOXA-58*	Multiplex PCR	*blaCTX-M-15* was the most frequent ESBL gene, followed by various *blaCTX-M* variants. Isolates often co-harbored narrow-spectrum β-lactamases like *blaTEM-1B* and *blaOXA-1*, while *blaAmpC* producers carried genes such as *bla-CMY-51* and *blaACT*-9. All ESBL and *blaAmpC*-producing isolates remained susceptible to carbapenems. Carbapenemase-producing isolates (CPEs) primarily harbored *blaKPC-2*, sometimes alongside *blaVIM-1* or *blaOXA-181*. Most CPEs also carried additional ESBL or *blaAmpC* resistance genes. Notably, one *C. freundii* isolate possessing both *blaKPC-2* and *blaVIM-1* was also found to carry the colistin resistance gene *mcr-9*.	[167]
18.	January 2021 – November 2023	49 hospital wastewater samples<br>28 community wastewater samples<br>29 wwtp influent samples<br>14 wwtp effluent samples.	Egypt: Sohag City	*blaTEM, blaSHV, blaCTX-M*<br>*blaIMP, blaVIM, blaKPC, blaNDM, blaOXA-48*	Standard PCR	Among 40 *K. pneumoniae* isolates, 67.5% carried only ESBL genes, while 32.5% were co-producers of ESBL and carbapenemase genes. The most prevalent ESBL genes were *blaSHV* (100%), *blaCTX-M* (60%), and *blaTEM* (60%), whereas carbapenemase profiles included *blaOXA-48* (22.5%), *blaNDM* (20%), and *blaKPC* (2.5%). *blaVIM* and *blaIMP* were not detected. Resistance prevalence was higher in hospital wastewater, specifically regarding *blaNDM*, which was absent in community wastewater. While five resistance genes were identified in influent samples, effluent isolates carried only *blaSHV*, *blaCTX-M*, and *blaOXA-48*.	[168]

19.	August 2020 and April 2021	81 hospitalized patient samples<br>73 hospital wastewater samples<br>21 river upstream samples<br>96 wwtp inflow samples<br>106 wwtp outflow samples.	Czech Republic	*blaCTX-M-15, blaCTX-M-14, blaCTX-M-27, blaCTX-M-1, bla-CMY-2, blaDHA-1, blaGES-5, blaOXA-244*	Culture-based method/ Standard PCR/ Illumina HiSeq4000	Of the 408 strains analyzed, 95% harbored ESBL genes, 3% carried *blaAmpC*, and 2% possessed carbapenemase genes, with *blaCTX-M-15* (49%) serving as the dominant ESBL marker. While most resistance genes were widely distributed, *blaCTX-M*-14 was primarily linked to wastewater.*blaAmpC* production was consistently represented by *blaCMY-2* and *blaDHA-1*. Carbapenemase-producing strains included those harboring *blaGES-5,* mostly from hospital/municipal wastewater, and *blaOXA*-244, mostly from municipal wastewater and river water. Carbapenem resistance, primarily to ertapenem, was confirmed in all *blaGES-5* isolates, one *blaOXA-244* strain, and 11 isolates where no specific carbapenemase gene was detected.	[169]
20.	June – July 2019	Hospital effluent samples	Peru: Lima	*blaCTX-M, blaSHV, blaTEM, blaPER, blaKPC, blaNDM, blaVIM, blaIMP*	Standard PCR	In the level II hospital, detected genes included *blaTEM* (21.4% in *E. coli* and *K. oxytoca*), *blaKPC* (28.6% in C. freundii, *P. aeruginosa*, and *V. fluvialis*), and *blaIMP* (7.1% in *Pseudomonas spp.*). In the level III hospital, resistance profiles showed higher prevalence: *blaTEM* (38.9%) was found across various species, including E. cloacae and *P. aeruginosa*, while *blaKPC* (44.4%) was primarily identified in *E. cloacae*, *V. fluvialis*, and *C. freundii*. Notably, *blaCTX-M*, *blaSHV, blaPER*, *blaNDM*, and *blaVIM* were absent in all tested isolates.	[170]
21.	*N.R.*	2 Burns Ditch samples<br>3 Coffee Creek samples	U.S.A.: Indiana	*blaCTX-M-1–15*, *blaSHV-2*, *blaCTXM-9–14*, *blaCMY-2*, *blaKPC*, *blaNDM*, *qnrS, mcr-1*,	RT-qPCR	In *E. coli* isolates, *bla-CMY-2* (100%) and *blaCTX-M-1–15* (96.8%) were the most prevalent, alongside frequent detections of *blaCTX-M-9–14* (77.4%), *qnrS* (70.9%), *blaSHV-2* (61.3%), and *blaKPC* (58.1%). Neither *mcr-1* nor *blaNDM* were present. In environmental water DNA, these genes appeared less frequently, likely due to lower *E. coli* abundance. *bla-CMY-2* was notably absent from environmental samples, and detections were	[171]

		1 Damon Run sample<br>4 Deer Creek samples<br>2 Dunes Creek samples<br>1 Lee Creek sample<br>1 Little Calumet River sample<br>6 Long Beach samples<br>2 Salt Creek samples<br>2 Sand Creek samples<br>1 Smith Ditch sample<br>4 Trail Creek samples<br>1 septic system pump-out sample				intermittent across time and sites. Among environmental samples*, blaSHV-2* was the most common (55.3%), followed by *blaCTX-9–14*, *blaKPC* (31%), and *qnrS* (27.6%).	

22.	December 2019	River water samples	Indonesia: Surabaya	*sul1, sul2, sul3, qacEΔ1, kdpE, acr, blaOXA, blaTEM, blaCTX-M, blaNDM, blaPOM, blaADC*	Culture-based method/Illumina MySeq	*sul1* and *qacEΔ1* were the most abundant genes. Aminoglycoside resistance was primarily driven by *aac* and *aph* gene families, indicating streptomycin as a significant selective agent, alongside high expression of *E. coli* efflux pumps *kdpE* and *acr*. Regarding β-lactamases, *blaOXA* and *blaTEM* were the most frequently detected, followed by *blaPOM-1* and *blaADC* in *Pseudomonas* and *Acinetobacter*, respectively. While *blaCTX-M* clusters were most prominent in river water near hospitals, the *blaNDM* gene cluster was specifically identified. *mcr* genes were also detected, but in low concentrations.	[172]
23.	January – December 2024	Hospital wastewater samples	Mexico: Mexico City	*blaKPC, blaOXA-48, blaIMP, blaVIM, blaOXA-40, blaOXA-23, blaNDM*	Standard PCR	The serine β-lactamase genes *blaKPC* and *blaOXA-48* were most prevalent in the emerging pathogen *A. caviae* and ESKAPE organisms like C. freundii and *E. coli*. Conversely, *blaIMP*, *blaVIM*, and *blaOXA*-40 were less frequently observed in Pseudomonas non-aeruginosa and A. baumannii. Notably, despite *blaOXA-40* and *blaOXA-23* typically being specific to A. baumannii, they were absent in all resistant isolates tested. Regarding co-resistance, ESKAPE bacteria (*E. coli*, *Enterobacter*, *K. pneumoniae*, and *P. non-aeruginosa*) exhibited combinations of serine and metallo-β-lactamases, specifically *blaKPC* + *blaNDM* and *blaVIM* + *blaOXA-48*. This *blaKPC* + *blaNDM* coexistence was also identified in *A. caviae*.	[173]
24.	2017 – 2018	Orontes River samples	Lebanon	*aac(3)-iIa, aac(3)-iId, aac(6')Ib-cr, aac(6')-Ic, aadA1, aadA2, aadA5, aadA16, aph(3')-Ia, aph(3'')-Ib, aph(6)-Id, , catA1, catA2, catB3, catB4, cmlA1, dfrA1, dfrA7, dfrA12, dfrA14, dfrA17, dfrA27, dfrA30, floR, FosA, mdf(A), mph(A), oqxA, oqxB,*	Standard PCR	Results showed 12 distinct β-lactam resistance variants, with *blaTEM-1B* being the most prevalent, appearing in 14 MDR isolates. *blaCTX-M-15* was detected in 13 isolates, with five of these also harboring *blaOXA-1*. *blaNDM-5* was the only carbapenemase gene identified, found in a single *E. coli* isolate. Additionally, 14 aminoglycoside resistance genes were detected, most notably *aph(3'')-Ib* (32%) and *aph(6)-Id*. The isolates also carried various determinants conferring resistance to phenicols, macrolides,	[174]

				qnrB1, qnrB4, qnrB6, qnrB66, qnrD, qnrS1, qnrS2, strA, strB, sul1, sul2, tet(A), tet(B),tet41, blaTEM-1B, blaCTX-M-15, blaOXA-1, blaOXA-132, blaNDM-5, blaCTX-M-15, bla-CMY-42, blaADC-25, blaDHA-1		tetracyclines, sulfonamides, fosfomycin, and quinolones.	
25.	October – November 2021	12 influent WWTP samples	Indonesia	*blaCARB, bla-CMY_2, bla-CMY2, blaCTX-M_5, blaCTX-M_8, blaGES, blaIMI, blaKPC, blaKPC_2, blaMOX/bla-CMY, blaNDM, blaOXA48, blaOXA51, blaSHV11, blaSME, blaTEM, blaVEB, blaVIM, and cfxA*	RT-qPCR	Monitoring of hospital wastewater over six weeks identified 17 distinct β-lactam resistance genes, 12 of which were consistently present despite fluctuations in relative abundance. While most targeted genes were detected in the wastewater, *blaIMI* and *blaSME* were absent, and five carbapenemase genes, *blaKPC*, *blaKPC2, blaOXA48, blaOXA51*, and *blaVIM,* were missing in several samples. In contrast, *E. coli* and *K. pneumoniae* isolates showed inconsistent resistance profiles, harboring only eight and seven of the detected β-lactam resistance genes, respectively, with none present across all sampling time points. There was no significant temporal variation in the relative abundance of either ESBL or carbapenem resistance genes.	[175]
26.	summer and winter 2021–2022	14 raw greywater samples<br>15 treated greywater samples	Israel: Midreshet Ben Gurion	*blaTEM, sul1, tetG, tetW, FabVas, FabVas2*	RT-qPCR	While seasonal differences occurred—such as the summer decrease of *fabVAS2* and the winter reduction of *tetG*, *sul1*, and *blaTEM*—the overall efficiency of the water treatment system was notably higher during winter. When comparing raw and treated samples, certain genes such as *sul1*, *blaTEM*, and *fabVAS2* showed higher relative abundance in treated samples during summer, whereas *tetG* and *intI1* levels fluctuated by season in raw influent. Despite these specific seasonal shifts, there was no statistically significant difference in the overall relative gene	[176]

						copy abundance between raw and treated greywater when aggregated across both seasons.	
27.	20 July – 27 September 2017	WWTP influent and effluent samples	Germany:Schleswig-Holstein	*blaCTX-M-15, blaCTX-M-1, blaCTX-M-14, blaCTX-M-27, blaCTX-M-9, blaCTX-M-14b, blaCTX-M-55 n = 1, blaCM*	Illumina NextSeq500	Among the 172 sequenced isolates from four wastewater treatment plants, 158 (91.9%) harbored known β-lactamase genes, with *blaCTX-M* the most prevalent (n=146), specifically *blaCTX-M-15* and *blaCTX-M-1*. While two isolates contained *bla-CMY, blaAmpC* genes, and none carried carbapenemases, seven isolates lacked detectable resistance genes entirely, despite confirmed phenotypic resistance to third-generation cephalosporins.	[177]
28.	2013 – 2014	15 hospital WWTP influent<br>15 hospital WWTP effluent samples	India: Mangaluru	*blaNDM-1*	Standard PCR	Among 15 phenotypically positive MBL-producing isolates, 9 were confirmed by PCR (*E. coli*: 4, *Klebsiella spp.*: 4, *Enterobacter spp.*: 1), though none from treated hospital effluent carried *blaNDM-1*. All 9 genotypically confirmed *blaNDM-1* positive isolates were multidrug-resistant, with two exhibiting resistance to 12 different antibiotics.	[178]
29.	March 2016 – April 2017	Samples from 3 different WWTP influents and effluents<br>160 fecal bird samples	Chile: Región de Los Ríos	*blaVIM, mecA, mecC, mcr-1, qnrA, vanA, vatC, vatE, vgbBhad,blaAmpC, bla-CMY, blaCTX, blaIMP, blaNDM-1, blaPER-2, qnrB, sul3, tet(L), tet(M), vatB, aacA, aadA5, blaKPC, blaNPS, blaOXA, blaSHV, blaTEM, dfr13, ermB, ermF, floR, qacG, qnrS, sul2, tet(A), tet (B), tet(Q), tet(S), tet(W), tet(X), vanB, strB*	Microfluidic quantitative PCR (MF-qPCR)	After excluding low-abundance or rarely detected ARGs from water and bird samples, 24 ARGs were retained for sediment analysis and 7 for bird analysis. In water samples, *sul1, qnrS, sul2*, and *strB* were most abundant, with concentrations peaking at 2.33 x 10^7, 1.07 x 10^8, 1.93 x 10^7, and 7.80 x 10^6 copies/g, respectively; site-specific dominance varied between *blaSHV, tet(C), sul1, qnrS, sul2,* and *strB*. Across all bird species and months, *blaKPC, blaSHV, blaTEM, sul1*, and *sul2* were identified as the most abundant resistance genes.	[179]

				sul1, tet(C) sul1, qnrS, sul2, and strB.			
30.	*N.R.*	Surface water samples	South Africa: Mooi River	*bla-CMY, acrAB-TolC, mdtG, acrD, acrF, acrB, baeR, mdtABCGHK, mdtF, emrAB-TolC, patA, mdtHK, qnrB, vexABCDE, tviBCDE, iroBCDEN, chu, ent, chuY*	Illumia MiSeq 2000	The study identified four ESBL-producing *Citrobacter* isolates from Mooi River surface water as *C. braakii*, *C. murliniae*, and *C. portucalensis*. All isolates showed phenotypic multidrug resistance, consistent with genomic detection of *bla-CMY* β-lactamase genes and multiple efflux pump systems. Pangenome analysis of 109 global *Citrobacter* genomes found that 83% carried one of 18 *bla-CMY* variants, with distinct variants dominant in *C. braakii* versus *C. portucalensis*; a quinolone-resistance gene, *qnrB13*, was unique to the isolate.	[180]
31.	March – August 2019	30 inlet, aeration lagoon, and effluent WWTP samples	Iran: Ardabil	*blaCTX-M-15, blaTEM-20, blaSHV-12,*<br><br>*blaDHA, blaCIT, bla FOX, blaMOX, bla CIT, blaDHA, bla ACC, bla CMY, and bla EBC*	Multiplex PCR	Among 146 Enterobacterales isolates recovered from the Ardabil urban WWTP, *blaCTX-M*, specifically *blaCTX-M-15,* was the dominant ESBL gene, with *blaTEM-20* and *blaSHV-12* also present, while *blaDHA* was the most common *blaAmpC* gene, followed equally by *blaCIT* and *blaFOX*. The majority of ESBL producers (75%) and over half of *blaAmpC* producers (55.5%) displayed multidrug-resistant phenotypes, with the highest resistance rates against ampicillin, nalidixic acid, and cephalexin.	[181]
32.	*N.R.*	Samaples from 2 wwtps	Czec Republic: Moravian: Silesian region	*blaTEM, blaKPC, blaNDM-1, blaOXA-48*	qPCR	qPCR screening confirmed the presence of *blaTEM* and *blaNDM-1* in WWTP bacterial populations, demonstrating high sensitivity and linearity. While *blaTEM* consistently showed significantly higher relative abundance in the sedimentation tank than in the nitrification tank of WWTP1, *blaNDM-1* exhibited the inverse trend, with higher abundance in the nitrification tanks of both plants at nearly all sampling times, suggesting that sedimentation processes specifically drive *blaTEM* accumulation.	[182]

33.	July – October 2017	WWTP effluents<br>River water<br>Sediment<br>Epilithic biofilm<br>Amphipod gut mictobiome	Switzerland	*blaOXA2, blaOXA9, blaOXA10, blaOXA12, blaOXA20, blaOXA60, blaOXA119, blaOXA129, blaOXA147, blaTEM-1, blaTEM-205, blaTEM-117, blaTEM-118, sul1, aadA, CfxA2, aph(3')-IIb, floR, vanG, arr, and vanR,tet39*	Illumina HiSeq 4000	A total of 677 ARG subtypes were detected, with 165 subtypes across 15 resistance classes. While clear habitat differences emerged, some high/low-abundance ARG patterns were shared across most habitats. Notably, several *blaTEM* genes (including *blaTEM-1, blaTEM-205, blaTEM-117*, and the ESBL gene *blaTEM-118*), along with *blaOXA-60, aph(3')-IIb, floR, vanG, arr,* and *vanR*, were found almost exclusively at high abundance in amphipod gut samples. In contrast, genes like *aadA, blaOXA-9/10/147, CfxA2, sul1*, and several tetracycline genes, *tet39,* were most abundant in the other habitats, especially in effluent-derived bacteria.	[183]
34.	May – April 2017	6 sewer water samples<br>3 wwtp influent samples<br>2 wwtp effluent samples<br>6 surface water	U.S.A.: Northern Colorado, Larimer County	*blaOXA, blaCTX-M, blaTEM*	Standard PCR	*blaCTX-M* and *blaTEM* were detected across all sample types and dates, consistent with reports of high *blaOXA, blaCTX-M*, and *blaTEM* prevalence in clinically resistant Colorado *E. coli*. *blaCTX-M's* presence was independent of water source and unrelated to clonal relatedness, likely reflecting multiple undistinguished gene variants common across environmental, human, and animal sources. In contrast, *blaOXA* appeared only in wastewater isolates from April 2017, suggesting it may help distinguish wastewater from ambient-water *E. coli*, consistent with prior studies showing *blaOXA* differentiates beta-lactam-resistant *E. coli* by water source.	[184]
35.	July – August 2022	2 river water samples<br>55 drinking water samples<br>53 environmental samples	Lebanon	*blaNDM-5, blaNDM-19, blaOXA-48, blaOXA-244, blaOXA-181, blaOXA-505, blaCTX-M-15, blaCTX-M-65, blaCTX-M-3, blaCMY-2, blaCMY-42, blaCMY-61, blaCMY-145, blaDHA-1, blaTEM-1, blaOXA-1, qnrB, qnrS1, gyrA, parC*	Illumina NextSeq 500	Among all the samples collected, carbapenemase-producing *E. coli* was detected in 5.3% of the water samples and 7.5% of the environmental ones. *blaNDM* and *blaOXA-48-like* were the only carbapenemases found, with *blaNDM-5* as the dominant gene, 82% overall.	[185]

36.	*N.R.*	Wastewater samples	Australia: Sydney	*blaNDM-5, blaIMP-4, blaOXA-1, blaCMY-2, blaCMY-14, blaCMY-42, blaCMY-58, blaTEM-1B, blaTEM-1C, blaGES-5, blaCTX-M-15, blaCTX-M-27, blaCTX-M-55, rmtB, aac(6')-Ib3, aac(3)-IId, aac(6')-Ib-cr, qnrS1, qnrS2, qnrA1,sul1, sul2, dfrA12, dfrA17, nfsA, gyrA, parCE, nfsB.*	Culture-based method/Illumina MiSeq	In the characterized *E. coli* isolates, multidrug resistance was driven by a complex interplay of acquired genes and chromosomal mutations. β-lactam resistance was primarily mediated by the co-carriage of carbapenemase genes *blaNDM-5, blaGES-5, blaIMP-4*, or ESBL *blaAmpC* genes *blaCTX-M, blaCMY-2*, occasionally supplemented by *ompC* mutations. Aminoglycoside resistance was attributed to modifying enzymes and methyltransferases, while fluoroquinolone resistance correlated strongly with mutations in *gyrA* and *parCE,* with some low-level resistance conferred by *qnrS1*. Finally, resistance to trimethoprim-sulfamethoxazole was linked to the prevalence of *dfr* and *sul* resistance genes, illustrating a multifaceted genomic basis for the observed multidrug-resistant phenotypes.	[186]
37.	May 2022 – August 2023	WWTP influent and effluent samples	South Korea: Suwon	*blaCTX, blaTEM, blaSHV, blaKPC, blaDHA, blaACC, blaFOX, blaNDM*	RT-dPCR	*blaDHA, blaACC, blaFOX, blaTEM, blaCTX*, and *blaKPC* — were present at high concentrations ranging roughly from 5.6 to 8.4 log10 gene copies/L and showed strong pairwise correlations with one another, notably *blaKPC–blaFOX* at r = 0.86 and *blaTEM–blaCTX* at r = 0.82, suggesting shared genetic or environmental drivers. At the same time, the high abundance of beta-lactam resistance genes highlights antibiotic resistance as a major public health concern in South Korea, and their persistence even in treated wastewater raises worries about the continued spread of resistance traits into surrounding water bodies and agricultural systems.	[187]
38.	*N.R.*	River water	France: Savoy, Rhône River, Germany: Karlsruhe, Alb River	*blaTEM, blaCMY-2, sul1*	Culture-based method, RT-qPCR	Over 10 weeks, two patterns emerged in both rivers regardless of temperature or wastewater addition. Wastewater-linked markers, total and ESBL-producing *E. coli*, *blaTEM*, and *blaCMY-2* decayed rapidly, mostly falling below detection within days to a few weeks. Environmental markers, R2A-culturable and *sul1,* declined more slowly and stayed detectable at high levels (10^4–10^7 copies/L) after 10 weeks, with *sul1* persisting	[188]

						longest and rising ~10-fold with wastewater addition before converging back to baseline by day 70. Cold incubation slowed early bacterial decline but not ARG decay.	
39.	*N.R.*	Hospital wastewater	China: Suzhou, Jiangsu Province	*blaNMD, blaOXA-48*	standard PCR/Illumina NovaSeq 6000	This study reported the first identification, in a hospital in China, of seven *Shewanella xiamenensis* strains isolated from hospital wastewater that co-produced the carbapenemase genes, *blaNDM-1* and *blaOXA-48-like*, with three distinct *blaOXA-48-like* variants detected — the first documented occurrence of all three variants within a single hospital setting in China. All seven strains tested positive for both *blaNDM* and *blaOXA-48* by PCR, and antimicrobial susceptibility testing confirmed that all isolates were resistant to a broad range of clinically important antibiotics.	[189]
40.	September 2016 – July 2017	Urban and rural wastewater<br>WWTP influents and effluents samples and the receiving rivers	Germany	*blaVIM, blaNMD, blaGIM, blaKPC, blaOXA-48, blaOXA-51, blaIMI*	Culture-based method, qPCR	Carbapenemase genes were confirmed in 134/171 (28.21%) phenotypically carbapenem-resistant strains in the clinical/urban system, including 23 with two different genes, versus only 8/18 (1.41%) in the rural system, where no strain carried more than one gene. The clinical/urban system harbored *blaGIM, blaKPC, blaNDM, blaOXA-48, blaOXA-51*, and *blaVIM*, with *blaVIM* and *blaNDM* especially common. The rural system had only *blaIMI* and low levels of *blaVIM*, with no *blaNDM* detected. Five carbapenemase-carrying strains (*blaIMI or blaVIM*) were found in rural surface water.	[190]
41.	*N.R.*	16 wastewater grab samples<br>18 floor drainage swabs	South Africa: North West Province	*laCTX-M-55, blaCTX-M-27, blaCTX-M-15, blaCTX-M-14, blaSHV-27, blaOXA-1, blaEC*	Culture-based method/Illumina NovaSeq 6000	The most common ESBL genes were *blaCTX-M-55* (60.0%) and *blaCTX-M-27* (20.0%) in *E. coli*, and *blaCTX-M-15* (50.0%) in *K. pneumoniae*, with two *K. pneumoniae* isolates (33.3%) carrying both *blaCTX-M-14* and *blaSHV-27*; one *K. pneumoniae* isolate had no ESBL gene. All isolates carried additional beta-lactamase genes (e.g., *blaOXA-1, blaEC*) plus up to 11 other AMR	[191]

						genes, with tetracycline resistance genes present in all *K. pneumoniae* and 60% of *E. coli* isolates. Most sequenced isolates were multidrug-resistant (80% of *E. coli*, all *K. pneumoniae*), though no colistin or carbapenem resistance genes were found.	
42.	Autumn 2016	Raw hospital sewage, inflow and outflow of WWTP samples	Czech Republic: Brno	*blaIMP, blaKPC, blaNDM, blaOXA-48, blaVIM, mcr*	Multiplex PCR/ Illumina MiSeq	Three of 180 screened coliform isolates (1.7%) were *Enterobacter asburiae* carrying *blaVIM*-1: one from the hospital WWTP inflow and two from the municipal WWTP, all multidrug-resistant. A broader phylogenomic analysis of 688 public *E. asburiae* genomes revealed carbapenemase genes in 39.5% overall (*blaVIM* in 3.6%, *blaKPC-2* in 8%, *blaNDM-1* in 5.8%, *blaKPC*-3 in 4.1%, *blaIMP*-1 in 4.5%, *blaOXA-48* in 2.8%) and *mcr* genes in 31.5%, often co-occurring with carbapenemases, suggesting *E. asburiae* carries these critical resistance genes far more often than previously recognized, with wastewater acting as a key reservoir and transmission route.	[192]
43.	March and September 2018	Hospital wastewater and Urban wastewater treatment plant (UWWTP) samples	Brazil: Ribeirão Preto, São Paulo	*blaTEM, blaSHV, blaCTX-M, blaKPC, blaKPC-2*	Culture-based method/Standard PCR	β-lactamase-encoding genes were detected in 35.6% of the Gram-negative bacilli isolates recovered from hospital sewage and urban wastewater in Ribeirão Preto, Brazil, with *blaKPC* and *blaTEM* being the most frequently detected genes (each in 17.8% of isolates), followed by *blaSHV* (13.3%) and *blaCTX-M* (8.9%). Isolates from hospital sewage showed significantly different (generally higher) resistance to amoxicillin-clavulanate, aztreonam, cefepime, and cefotaxime compared with urban sewage isolates, and a multidrug-resistant (MDR) phenotype was observed in 33.3% of hospital sewage isolates. Notably, this study represented the first report of the carbapenemase gene *blaKPC-2*, associated with carbapenem-resistant Enterobacteriaceae, being detected in hospital sewage in this region of Brazil.	[193]

44.	June 2019	Surface water samples	Dominican Republic: Isabel River, Santo Domingo	*blaTEM, blaSHV, blaVIM, blaOXA, blaIMP, blaKPC, blaKPC-3, blaOXA-1, blaOXA-72, blaOXA-132, blaCTX-M-55, blaCTX-M-15, blaTEM-1, blaACT-9*	Multiplex PCR/Illumina NovaSeq 600	Of 20 clinically important isolates *blaTEM* was detected in in 25% (mostly Enterobacteriaceae), *blaOXA* in 10% (only *E. coli*), *blaSHV* in 5%, and *blaKPC* in one *Enterobacter kobei* isolate. Whole-genome sequencing confirmed *blaKPC-3* in that *E. kobei* strain (plasmid-borne, alongside qnrS), *blaOXA-72* and *blaOXA-132* in all seven sequenced *A. baumannii* isolates, *blaCTX-M-15* and *blaCTX-M-55* (often with *blaTEM-1*) across multiple *E. coli*. *Enterobacter* and *Klebsiella* carried the broadest resistance gene repertoires, including carbapenem resistance, while *Acinetobacter* relied more on porin loss/efflux pumps than acquired β-lactamases.	[194]
45.	July 2015 – November 2016	99 drinking water samples from Wisconsin<br>22 drinking water samples from New York<br>69 drinking water samples from Pennsylvania<br>125 drinking water samples from Illinois<br>64 drinking water samples	U.S.A.	*blaSHV, blaTEM, blaCTX-M, blaOXA-48-type, blaNDM, blaKPC*	Culture-based method/Multiplex PCR	Of 483 coliform-positive samples, 31 (6.4%) from 26 water systems tested positive for target genes, with at least one positive sample in every state. *blaCTX-M* and *blaSHV* were each found in 13 samples (2.7%), *blaTEM* in 10 (2.1%); no *blaNDM* or *blaKPC* was detected, but *blaOXA-48-type* genes appeared in 19 samples (3.9%), three of which co-occurred with *blaCTX-M*. Bacteria were isolated from 21 of the 31 positive samples*: blaCTX-M* was confined to Enterobacteriaceae (*Klebsiella oxytoca*, *Citrobacter freundii* complex, *Kluyvera*), *blaSHV-38* was found inand the ESBL-phenotype *blaTEM* variant in *E. coli*, while *blaOXA-48-type* genes appeared in Enterobacteriaceae and broadly in non-fermenters. ESBL genes remained stable across subcultures, while *blaOXA-48-type* genes were often lost after one subculture in non-*Shewanella* non-fermenters.	[195]

		from Arkansas<br>104 drinking water samples from Utah					
46.	2009 – 2013	Hospital untreated and ground wastewater<br>Agriculture effluent samples<br>Partially treated wastewater from the wastewater oxidation pond<br>River water samples	Nigeria	*blaSHV, blaCTX-M-15, blaTEM, blaFOX*	Standard PCR	The study confirmed ESBL production in 114 (79.7%) of the 143 isolates, with ESBL genes, *blaSHV, blaCTX-M-15*, and *blaTEM* successfully detected in 85 (74.6%) of these ESBL-producing isolates.	[196]
47.	October – November 2013	90 water samples<br>95 soil samples	Algeria: Béjaïa	*blaAmpC, blaIMP, blaNDM, blaKPC, blaOXA-48, blaGES*	Culture-based method/Standard PCR	While 62 antibiotic-resistant strains were isolated and categorized into several families. The molecular screening for known carbapenemase genes in transconjugants and transformants, *blaAmpC, blaIMP, blaNDM, blaKPC, blaOXA-48*, and *blaGES*, yielded negative results.	[197]
48.	September 2014	Hospital untreated wastewater	Singapore	*blaSHV, blaNDM-1, blaCTX-M, blaKPC*	Culture-based method/Standard PCR	High prevalence of multidrug-resistant bacteria was observed in hospital effluents, with 66 isolates recovered from selective media showing significant resistance to third-generation	[198]

						cephalosporins and carbapenems. Molecular analysis confirmed the presence of clinically significant resistance genes, with *blaSHV* being the most predominant (41.1%), followed by *blaNDM-1* (35.6%), *blaCTX-M* (35.6%), and *blaKPC* (28.8%).	
49.	November 2020 – January 2021	Sewage samples from WWTP and homless shelter	U.S.A.: Nevada, Las Vegas	*tetQ, ermF, tetX, msrE, tetW, mphD, ermG, tetP, mefA, ermB*	Illumina NextSeq 500	In wastewater treatment plant samples, tetracycline resistance was the dominant gene class (63%), while shelter samples showed a more balanced distribution of tetracyclines (35%), beta-lactams (28%), and MLS (27%). Across both locations, a total of 238 AMR genes were detected, with *tetQ, tetW, msrE, ermF*, and *tetX* featuring prominently among the most abundant genes.	[199]
50.	June and October 2013	WWTP influent and effluent samples<br>Svartån River water samples<br>Hjälmaren Lake water samples<br>Oset pond water samples	Sweden: Örebro	*blaCTX-M, blaCTX-M-15, blaCTX-M-14, blaCTX-M-27*	Illumina MiSeq	The distribution of ESBL genes was similar between sources, with *blaCTX-M-15* dominating in both clinical (60.0%) and environmental isolates (62.2%), followed by *blaCTX*-M-14 (8.9% vs. 12.2%) and *blaCTX*-M-27 (6.7% vs. 8.5%), while the remaining ESBL genotypes occurred only sporadically.	[200]
51.	December 2018 – November 2019	Surface water samples	Nigeria: Edo State	*blaCTX-M-1, blaCTX-M-2, blaCTX-M-8, blaCTX-M-9, blaCTX-M-14, blaCTX-M-15, blaOXA-1, blaOXA-47, blaCMY-2, blaNDM-1,*	Standard PCR	Among the 49 ESBL-producing *E. coli* isolates, *blaCTX-M-1* was the most common ESBL gene (89.8%), followed by *blaCTX-M-15* (77.6%) and *blaTEM* (59.2%), while *blaOXA-1* was rare (4.1%); *blaSHV, blaCMY-2, blaOXA-47, blaNDM-1*, and other *blaCTX-M* variants (groups 2, 8, 9) were absent, showing that *blaCTX-M-1* and *blaCTX-M-15* dominated. Other resistance	[201]

				tetA, tetB, tetC, tetM, sul1, sul2, sul3, qnrA, qnrB, qnrC, qnrS, aacC(3)-1, ant(4')-Ia, cat1, cat2, cat3		genes were also widespread, *tetM* led among tetracycline genes (38.6%), *sul1* among sulfonamide genes (36.8%), and *qnrS* among quinolone genes (9.6%).	
52.	October 2020 – February 2021	Untreated wastewater from 2 hospitals	Brazil	*blaKPC1-5, blaIMP, blaVIM, blaSHV, blaTEM, blaOXA-1-like, blaOXA-48-like, blaCTX-Mgp1, blaCTX-Mgp2, blaCTX-Mgp9, blaOXA-23, blaOXA-24-like, blaOXA-51, blaOXA-58, blaNDM, aacA, aacA1, aac(6')-Im, aacC1, aadA4, aph, aphA, aph2, strA, strB, mph(A), mph(B), ermA, ermB, mefA, mel, qnrA3, qnrB1, qnr, tetA, tetD, tetM,mcr1, mcr2, mcr3, mcr4, mcr5, mecA, vanA, vanB*	Multiplex PCR	Of 36 cephalexin-selected strains, 86.1% carried a β-lactamase gene, mainly *blaTEM* (n=22, chiefly *K. pneumoniae*/*Enterobacter spp.*) and *blaKPC* (n=17, mainly Enterobacter spp.; significantly higher in H1), followed by *blaOXA-1-like* (n=9); *blaCTX-M* variants were rare, and *blaIMP, blaVIM, blaNDM*, and *blaOXA-23/24/48/51/58-like* genes were absent. Among 28 erythromycin-selected strains, macrolide genes were common — *mph(A)* (n=23), *mel* (n=18), *ermB* (n=16) — *while mph(B), ermA, and mefA* were absent. Nearly all 31 tetracycline-selected strains carried resistance genes, led by *tetD* (87.1%), *tetM* (83.9%), and *tetA* (74.2%), with *tetA* and *tetM* differing significantly between hospitals. Among 39 amikacin-selected strains, *strB* (n=18) and *strA* (n=11) dominated, with *strB* significantly higher in H1, while *aacA*, *aadA4*, and *aph* were less common and *aphA*, *aph2*, *aacC1*, *aac(6')-Im*, and *aacA1* were absent. No quinolone (*qnr, qnrA3, qnrB1*) or colistin (*mcr1–5*) genes were found; only two *S. haemolyticus* isolates carried *mecA*; no *Enterococcus* grew on vancomycin medium, so *vanA/vanB* were not assessed.	[202]
53.	October 2018	22 hospital wastewater grab samples<br>19 urban wastewater influent samples<br>17 urban wastewater	U.S.A.: Wisconsin	*blaCTX-M, blaCTX-M-1, blaCTX-M-2, blaCTX-M-9,blaTEM; blaSHV; blaOXA; blaKPC, blaKPC-2, blaTEM-1, blaCTX-M-15, blaOXA-1, tetB/tetA, mdf(A),blaAmpC, kdpE, emrAB, emrKY, acrAB-tolC, acrAD-tolC, mdtEF, mph(A), sul1,*	Culture-based method/Quadruplex PCR/Illumina NextSeq2000	Of seven β-lactamase genes screened, *blaCTX-M-1* was the most common overall (81%), highest in hospital WW (95%) and urban WW (73–81%), lower in clinical isolates (64%), and significantly linked to ceftriaxone/cefotaxime resistance. *blaCTX-M-9* was significantly more common in urban WW, and *blaCTX-M-2* was absent. *blaTEM* and *blaOXA* were each present in 73% of isolates but showed opposite patterns: *blaTEM* was highest in clinical isolates (100%) and lowest in hospital WW (59%), while *blaOXA* was highest in	[203]

		effluent samples		*dfr17, aadA5, catB3, aac(6')-Ib-cr, qacE, sitABCD*		hospital WW (86%) and lowest in clinical isolates (45%); *blaSHV* was never detected. *blaKPC* occurred only in hospital WW isolates (41%), linked to carbapenem/ceftazidime resistance and *blaTEM* absence. The class 1 integron gene, *int1*, was widespread and similarly common (~65%) across clinical and WW isolates, reflecting anthropogenic pollution and horizontal gene transfer.	
54.	December 2020 – September 2021	Water samples from 2 rivers	Thailand: Chiang Rai	*blaCTX-M, blaCTX-M-15, blaCTX-M-55, blaCTX-M-14, blaCTX-M-27, blaTEM-1, blaTEM-116, blaSHV*	Culture-based method/Multiplex PCR/Illumina HiSeq 2500	All 38 ESBL isolates carried a *blaCTX-M* gene, most commonly *blaCTX-M-15* (44.7%, 17/38), followed by *blaCTX-M-55* (26.3%, 10/38), *blaCTX-M-14* (18.4%, 7/38), and *blaCTX-M-27* (10.5%, 4/38). *blaTEM-1* and *blaTEM-116* co-occurred with *blaCTX-M* in 23.7% (9/38) and 2.6% (1/38) of isolates, respectively, while *blaSHV* was not found in any isolate. Integrase genes were also common: *int1* was present in 55.3% (21/38) and *int2* in 5.3% (2/38), with one isolate carrying both.	[204]
55.	April 2016	River waster samples	India: Pune City, Maharashtra	*blaKPC, blaCTX-M-9, blaSHV(156G), blaSHV, blaIMP-1, blaVIM-13, blaVIM-1, blaNDM, blaACT-5/7, blaACT-1, blaCMY-10, blaDHA, blaFOX, blaLAT, blaMIR, blaMOX, blaCFE-1, blaOXA-48, blaOXA-23, qnrB-31, qnrC, qepA, vanB, ermA, msrA.*	qPCR/Illumina HiSeq	Most of the 16 Class A β-lactamase genes were found at all locations, with the notable exception of *blaKPC*, which was absent in the reservoir. Out of seven tested *blaSHV* variants, only *blaSHV(156G*) and *blaSHV(238G240E)* were present in the water samples. Among Class B genes, *blaNDM* and *blaVIM-1group* were detected everywhere, whereas *blaIMP-1group* and *blaVIM-13* were absent from the upstream reservoir. Most Class C genes (*blaACT-5/7, blaACT-1, blaCMY-10, blaDHA, blaFOX, blaLAT, blaMIR, and blaMOX*) were ubiquitous, while *blaCFE-1* was only found once. For Class D, eight of the 13 genes were found in all locations, with *blaOXA-23* being the only one missing from the upstream reservoir. Other resistance mechanisms for fluoroquinolones, aminoglycosides, tetracyclines, and macrolides were widespread across the study area. Specifically, *qnrB-31, qnrC,* and *vanB* were not detected at any of the sites. Other genes,	[205]

						including *qepA, ermA*, and *msrA*, were only identified in isolated replicates or limited locations, suggesting their presence in the water may be transient.	
56.	20 – 28 February 2020	Untreated urban wastewater samples<br>Environmental water samples	Republic of Djibouti	*blaTEM, blaSHV, blaCTX-A, blaCTX-B, blaOXA-23, blaOXA-58, blaOXA-24, blaOXA-48, blaNDM, blaKPC, blaVIM, mcr-1, mcr-2, mcr-3, mcr-4, mcr-5, mcr-8*	Culture-based method/ Standard PCR and RT-qPCR	Class A β-lactamase genes were universal across all 11 isolates, with *blaCTX-M-15* and *blaTEM* variants identified in 81.8% of samples, indicating widespread dissemination between urban and environmental zones. On the other hand, *blaSHV* variants were limited to *K. pneumoniae* in urban wastewater, and carbapenemase genes, specifically *blaOXA-48* or *blaOXA-181*, were restricted to isolates from hospital wastewater. No *mcr* genes were detected, aligning with the observed universal susceptibility to colistin.	[206]
57.	2019	Untreated hospital wastewater samples	Iran: Tabriz	*blaSHV, blaTEM, blaCTX, blaVIM, su1l, qnrS*	Culture-based method/Standard PCR	Among the five bacterial isolates analysed for resistance genes, Escherichia coli strains showed consistent presence of *sul1, blaSHV*, and *blaTEM*, while select strains also harboured *blaCTX* and *qnrS*. The *sul1* gene was universal across all isolates. Among the identified *Pseudomonas aeruginosa*, the sulfamethoxazole resistance gene was the most abundant, followed by *blaVIM* (60%), *blaTEM* (60%), *blaSHV* (60%), and *blaCTX* (40%), while *qnrS* was detected in only a single *P. aeruginosa* isolate.	[207]
58.	January and May 2017	River water samples<br>Wastewater river inflow<br>WWTP samples	Columbia: Cali City	*blaTEM, blaSHV, blaCTX-M*	Culture-based method/Standard PCR	The prevalence of *bla* genes among the 166 analyzed isolates was high, with 75% and 88.4% of isolates testing positive during the dry and wet seasons, respectively, and over 30% of these carrying two or more resistance genes. The wastewater inflow sample demonstrated the highest concentrations of *bla*-positive isolates, reflecting the significant impact of domestic and industrial wastewater, while the Cauca River showed a 24.4% positive rate during the wet season. Molecular characterisation revealed that *blaVIM-2* was the most frequent resistance	[208]

						determinant (49.4%), followed by *blaTEM-1* (45.2%), *blaIMP-1* (22.9%), *blaAmpC* (20.5%), and *blaCTXM-9* (14.5%), though 16.9% of the isolates did not show amplification for the targeted genes.	
59.	*N.R.*	Surface water samoles, river samples, sediment samples, WWTP influent and effluent samples	Puerto Rico	*blaKPC, blaIMP, blaOXA, blaVIM, blaGES, blaCMY, blaCTX-M, blaSHV, blaTEM, blaMOX, sul1*	qPCR/Illumina NovaSeq 6000	WWTPs serve as a primary driver of riverine resistomes, with both the relative and absolute abundance of total ARGs increasing markedly downstream of WWTP discharges. These downstream areas showed a significant shift in resistome composition and a striking increase in β-lactam ARG richness, featuring mobile genes associated with pathogens of regional clinical concern. The detected β-lactamase genes included *blaIMP* (20 variants), *blaKPC* (4 variants), *blaOXA* (51 variants), *blaVIM* (1 variant), *blaGES* (5 variants), *blaCMY* (10 variants), *blaCTX-M* (12 variants), *blaSHV* (9 variants), *blaTEM* (9 variants), and *blaMOX* (9 variants).	[209]
60.	January 2021 – November 2023	49 hospital wastewater samples 28 community wastewater samples 29 wwtp influent samples 14 wwtp effluent samples	Egypt: Sohag City	*blaTEM, blaOXA-48, blaKPC, blaNDM, blaIMP, blaVIM, blaSHV, blaCTX−M*	Standard PCR	Among 58 *E. coli* isolates resistant to beta-lactam antibiotics, 82.8% (48/58) tested positive for at least one beta-lactamase or carbapenemase gene. The most prevalent extended-spectrum beta-lactamase (ESBL) genes were *blaTEM* (62.1%) and *blaCTX-M* (58.6%), while *blaSHV* was rare (6.9%); among carbapenemase genes, *blaOXA-48* (24.1%) was most common, followed by *blaKPC* (12.1%), with *blaIMP* absent entirely. The distribution of these genes varied significantly by source ($p = 0.008$ for *blaTEM* and *blaCTX-M*), with *blaCTX-M* predominating in hospital wastewater and treated WWTP effluent, whereas *blaTEM* was the most frequent gene in community wastewater and untreated WWTP influent.	[210]

61.	2020 – 2021	Wwtps water samples	Finland	*blaOXA-10, blaGES, blaACT, blaOXA-1, blaOXA-30, blaOXA-58, blaIMP, blaOXA-23, blaIMI, blaKPC, blaSHV, blaTEM, blaGES, blaACT, blaCTX-M*	RT-qPCR/HT-qPCR	The most prevalent resistance determinants were *blaOXA-10, blaGES, blaACT, blaOXA-1/blaOXA-30*, and *blaOXA-58*, whereas the least abundant included carbapenemase genes such as *blaSME, blaIMP, blaOXA-23, blaIMI*, and *blaKPC*. Notably, *blaSHV, blaOXA-1/blaOXA-30, blaTEM, blaCTX-M, blaACT*, and *blaGES* appeared in every sample.	[211]
62.	December 2016	Influent and effluent samples from four hospitals	China: Beijing	*blaSHV,* *blaTEM, blaCTX-M, blaNDM, blaKPC, blaIMP, blaVIM, blaOXA-48, mcr-1, mcr-2*	Standard PCR/Sequencing	Molecular analysis revealed that 9 *E. coli* isolates harboured the *mcr-1* gene while 12 carbapenem-resistant isolates carried *blaKPC-2* or *blaNDM-1*. These isolates also frequently carried other β-lactamase genes, including *blaTEM-1, blaSHV-12*, and *blaCTX-M-14*. Conjugation experiments confirmed the horizontal transferability of *mcr-1, blaKPC-2*, and *blaNDM-1* genes into *E. coli* recipient EC600, with *blaNDM-1* exhibiting the highest transfer frequency.	[212]
63.	2019 – 2021	River water samples	Germany	*blaTEM, blaCMY-2, blaOXA-48, blaVIM-2, blaSHV, blaNDM-1*	qPCR	qPCR confirmed the presence of clinically significant resistance genes, *blaTEM, blaCMY-2, blaOXA-48, blaVIM-2, blaSHV*, and *blaNDM-1*, at concentrations between 1.0 and 3.7 log10 gene copies per 100 mL. These findings indicate that oligotrophic environmental bacteria constitute a significant, often overlooked reservoir of antibiotic resistance genes, contributing to the broader dissemination of cephalosporinase and carbapenemase enzymes in aquatic environments.	[213]
64.	Fall 2017 – Summer 2018	River water and streams samples WWTP influent and effluent samples	U.S.A.: Georgia	*ermB, tetB, qnrS, blaCTX-M, blaSHV, blaKPC*	RT-qPCR	ARGs were identified in 67.2% of water sites sampled across the study, with *ermB* appearing most frequently (33.3% of total surface water samples), followed by *qnrS* (18.2%), *blaKPC* (15.4%), *tetB* (10.1%), *blaSHV* (9.4%), and *blaCTX-M* (2.0%). Seasonal analysis revealed that fall experienced the highest ARG prevalence (63.2% of sites), with specific locations testing positive for all six targeted genes, while detection rates during summer, winter, and spring were	[214]

						35.0%, 31.6%, and 23.5%, respectively. Excluding *ermB* in the fall, which reached a 60.5% detection rate, no other gene exceeded a 30% detection frequency in surface water throughout the year; notably, every wastewater influent sample remained positive for all six ARGs across all seasons.	
65.	July 2017	Storm drain outfall (SDO) samples	U.S.A.: Florida, Tampa Bay	*acrD, aadA5, blaAmpC, blaKPC, blaNPS, blaOXA, blaSHV, cadA, catB8, chrA, cmlB, copA, ereB, imp13, mefE, mer(A), mexB, nikA, qacF, qnrA, qnrB, rcnA, str(B), sul1, sul2, sul3, tet(A), tet(L), tet(M), tet(S), tet(W), tet(X)*	High-throughput microfluidic qPCR (MFQPCR)	35 out of 47 tested ARGs were detected in one or more samples with *intI1, sul1, tet(A), mer(A), str(B), blaSHV*, and *qacF,* demonstrating high prevalence (>70%) regardless of weather conditions. 27 ARGs, *includingblaAmpC, intI2, mexB, rcnA, and tet(W)*, exhibited higher prevalence during wet weather, with the latter five showing significantly increased concentrations, whereas *aadA5, blaNPS, copA*, and *qacF* were more prevalent or stable during dry weather. Moderate prevalence (25–70%) was observed *for sul2, mexB, aadA5, cadA, qnrA, blaAmpC, intI3, tet(X), tet(W), nikA, rcnA*, and *chrA*, while low prevalence (<25%) was noted for *acrD, blaKPC, blaNPS, blaOXA, catB8, cmlB, copA, ereB, imp13, intI2, mefE, sul3, qnrB, tet(L), tet(M), and tet(S).*	[215]
66.	October – November 2021	Surface river water samples	U.S.A.: Los Angeles	*sul1, tetW, blaSHV, ermF*	qPCR	qPCR analysis of ARGs *sul1, ermF, blaSHV, tetW*, and *intI1* revealed that genetic abundances were lowest in undeveloped areas, intermediate at beaches, and highest in developed sites. Developed locations, specifically the LA River, consistently exhibited significantly higher gene levels than undeveloped or beach sites ($p < 0.05$). While differences between developed and undeveloped land use were statistically significant for all genes, there was no significant difference between beach and undeveloped sites.	[216]
67.	*N.R.*		China: Nanchong	*penA, blaTLA, blaTEM, tetR-02, qacEdelta1-01/02, aadA1, aadA5-*	RT-qPCR/HT-qPCR	The analysis of ARG profiles reveals distinct distribution patterns across water samples. Several genes, including *penA, blaTLA, blaTEM, tetR-02,*	[217]

		8 river water samples<br>6 river branches water samples	City, Jialing River	*01, penA, blaTLA, blaVIM, mdtA, ermJ_ermD, mphA-01, ermC, mtrC-01, sulA-folP-03, tnpA-04*		*and qacEdelta1-01/02* (multidrug), were consistently abundant in all samples. 75.6% of ARGs were shared between the mainstream and its branches, while 10.9% and 13.5% were unique to each, respectively. Among shared genes, those conferring resistance to aminoglycosides (*aadA1, aadA5-01*), beta-lactams (*penA, blaTLA, blaVIM*), MLSB (*mdtA, ermJ_ermD, mphA-01, ermC*), multidrugs (*qacEdelta1-02, mtrC-01*), tetracyclines (*tetR-02*), and sulfonamides (*sulA-folP-03*) were significantly more enriched in the branches than in the mainstream ($p < 0.01$), with mdtA (MLSB) showing the highest enrichment at 182.57-fold ($p < 0.001$). While ARG patterns varied by branch, both mainstream and branch samples shared 12 mobile genetic elements, with *tnpA-04* and *intI-1LC* being consistently prevalent.	
68.	2009 and 2012	River water samples and hospital sewage samples	Brazil: Porto Alegre City, Dilúvio River	*blaTEM-1, blaSHV-1, blaSHV-5, blaCTX-M-8, blaKPC, blaGES, blaACT-1, blaMIR-1*	Multiplex PCR/ABI-Prism 3500 Genetic Analyzer	Multiplex PCR and sequencing identified β-lactamase genes in 75% (30/40) of isolates across various species (*K. pneumoniae*, *K. oxytoca*, *E. cloacae*, and *E. ludwigii*). The most prevalent gene was *blaTEM* (70%), followed by *blaSHV* (17.5%) and *blaCTX-M* (10%). Notably, 35% of isolates harbored two resistance genes, while 20% contained three (spanning BL, ESBL, and carbapenemase classes). *blaKPC-2* was the most frequently detected Class A carbapenemase, and GES-family carbapenemases were found in 20% of hospital-derived K. pneumoniae. Only one isolate carried *blaAmpC* (*blaACT-1* or *blaMIR-1*), and 15% of all strains tested negative for the genes investigated.	[218]
69.	*N.R.*	Recreational water samples<br>Hospital WWTP samples	Nigeria: Kaduna State, Zaria	*blaSHV, blaTEM, blaCTX*	Culture-based method/Multiplex PCR	*blaTEM* was the most frequently detected gene, while *blaCTX* was the least common. Notably, one *E. coli* isolate (B2A) from recreational water harbored all three targeted genes, highlighting a potential public health risk for residents using the lake for domestic and irrigation purposes. Most other positive isolates carried two genes, typically a *blaTEM* and *blaSHV* combination in *Klebsiella*	[219]

						spp., whereas *blaCTX* was exclusively found in *E. coli* strains.	
70.	21 October 2018	3 water samples<br>3 sediment samples	Ecuador	*blaCTX-M-12, blaOXA-10, blaKPC-2, fosA, aac(6')-lb, sul2, msr(E), mph(A)*	Standard PCR/Illumina MiSeq	Genomic analysis identified a robust resistome within the isolate, including *blaCTX-M-12, blaOXA-10, blaKPC-2, aac(6')-lb, sul2, dfrA14, fosA, msr(E), and mph(A).*	[220]
71.	January – June 2017	Water samples	Sudan: Khartoum	*blaNDM, blaIMP, blaVIM, blaSPM,*<br>*blaKPC, blaOXA_48*	Standard PCR	This study provides evidence of carbapenem-resistant *E. coli* in the drinking water of Khartoum, Sudan, with 28% of water isolates testing positive for at least one carbapenemase-encoding gene. Among the targeted genes, *blaOXA-48* was the most prevalent (15.5%), followed by *blaSPM* (8.8%) and *blaKPC* (4.4%), while *blaNDM*, *blaVIM*, and *blaIMP* were not detected in any of the samples.	[221]
72.	February – December 2018	50 well water samples<br>50 fish market effluent samples	India: Mangaluru	*blaCTX, blaSHV, blaTEM*	Standard PCR	The study identified a high prevalence of multidrug-resistant K. pneumoniae across the sampled environments, with the highest contamination observed in fish market effluents. Molecular screening confirmed the presence of ESBL-encoding genes in the isolates, with *blaTEM* being the most frequently detected (42.85%), followed by *blaCTX-M* (22.85%) and *blaSHV* (14.28%).	[222]
73.	September and December 2017	12 Matsumoto city WWTP inflow samples (A)<br>2 Nagano city from one WWTP's	Japan	*blaCTX-M-1, blaCTX-M-2, blaCTX-M-3, blaCTX-M-14, blaCTX-M-15, blaCTX-M-24, blaCTX-M-27, blaCTX-M-55, blaCTX-M-64, blaCTX-M-123, blaCTX-M-174*	Standard PCR/Sequencing ABI 3730xl genetic analyzer	Genomic analysis of 73 *E. coli* isolates from the WWTPs inflow samples revealed 31 distinct clonal lineages characterised by diverse *blaCTX-M* repertoires. In WWTP A, 50 isolates assigned to 25 sequence types (STs) were dominated by *blaCTX-M-14, blaCTX-M-15,* and *blaCTX-M-27*, with rare chimeric genes *blaCTX-M-64* and *blaCTX-M-123* also identified. Analysis of an additional 23 isolates from WWTPs B, C, and D further confirmed one isolate with *blaCTX-M-174*	[223]

		inflow samples (B)<br>2 Nagano city from the second wwtps inflow samples (C)<br>Azumino city WWTPS inflow samples (D)				alongside other varied STs harboring *blaCTX-M-14*.	
74.	April, June, and October 2014	WWTP influent and effluent samples	Croatia: Zagreb	*blaOXA-23-like, blaOXA-51-like, blaOXA-58-like, blaOXA-143-like, blaOXA-40-like*	Multiplex PCR	All isolates were classified as multidrug-resistant, harbouring the *blaOXA-51* gene, and acquired resistance genes were also identified. *blaOXA-23* was present in isolates IN4, IN18, and EF2, and *blaOXA-40-like* was found in isolate IN12. Phylogenetic analysis clustered these genes into three distinct groups, with sequence analysis showing 100% identity to clinically derived genes specifically *blaOXA-51-like* variants, *blaOXA-23*, and *blaOXA-72*.	[224]
75.	February, April and May 2012	Water discharge from two pharmaceut ical locations	Nigeria: Lagos and Ogun States	*blaTEM-like, blaSHV-like, blaCTX-M-like, blaCMY-like,*<br>*blaDHA-like, blaOXA-48-like, blaKPC-like, blaVIM-like, blaIMP-like, blaNDM-like*	Multiplex PCR/Sequenci ng	PCR screening and sequence analysis identified a diverse array of β-lactamase genes across the *K. pneumoniae* isolates. Among the six isolates exhibiting an ESBL phenotype, *blaCTX-M-15, blaSHV-2, blaSHV-12, blaTEM-1, blaSHV-1, blaSHV-11*, and *blaSHV-28* were detected. The single isolate lacking the ESBL phenotype harboured *blaTEM-1* and *blaSHV-11*.	[225]

76.	*N.R.*	48 irrigation water samples<br>13 freshwater stream samples<br>63 soil samples	South Africa	*blaCTX, blaTEM, blaOXA, blaSHV, sul1*	Standard PCR	Among the ESBL-producing *Salmonella spp.* isolates, *blaOXA* was the most frequently detected resistance gene at 75% (30/40), followed by *blaCTX-M* at 67.5% (27/40), *blaTEM* at 40% (16/40), and sul1 at 30% (12/40). Specifically, nine isolates recovered from soil and manure simultaneously harboured both *blaCTX-M* and *blaOXA*, while eight isolates from irrigation water and nearby freshwater streams harboured *blaOXA*. Notably, the *blaSHV* gene was absent across all sampled matrices.	[226]
77.	2017 – 2018	74 urban wastewater samples<br>80 rural wastewater samples<br>75 river water samples<br>71 pond water samples	Bangladesh	*blaCTX-M-1, blaCMY-2, blaTEM, blaSHV, blaOXA-1, blaOXA-47, qnrS, qnrB, oqxAB, qepA, aac(6')-Ib-cr, qnrA, qnrC, qnrD*	Standard PCR	Of 300 aquatic *E. coli* isolates, 66% (n=199) tested positive for PMQR genes, with *qnrS* being the most prevalent (82%). While most isolates carried a single PMQR gene, ten contained multiple co-occurring variants. None of the isolates was positive *for qnrA, qnrC* and *qnrD*. 87% of isolates were also confirmed as ESBL producers, primarily harbouring *blaCTX-M-1,* followed by *blaTEM*, *blaOXA-1, blaOXA-47 and blaCMY-2,* respectively. Additionally, 10% of these isolates carried the carbapenemase gene *blaNDM*-1, and 37% were positive for the class 1 integron gene *int1*. Statistical analysis revealed significant correlations between specific resistance determinants: for instance, *qnrS* was linked to *blaCTX-M-1*, while *qnrB* showed strong associations with *blaTEM, blaOXA-1*, and *blaNDM-1*.	[227]
78.	*N.R.*	17 surface river water samples<br>43 wwtp samples	Tunisia	*blaCTX-M, blaTEM, blaSHV*	Standard PCR	PCR screening for ESBL-encoding genes showed that *blaCTX-M* was the predominant determinant among these isolates, with *blaSHV* detected only sporadically and *blaTEM* not found, consistent with the overall study finding that *blaCTX-M* accounted for 87.5% (41/48) of all ESBL-positive isolates across both water and animal sources.	[228]

79.	28 February – 1 of March 2016<br>Spring 2017<br>17 – 24 July 2019	12 surface water samples<br>16 urban water pond samples<br>Wwtp influent and effluent samples	China: Xiamen City	*acrA-01, cfiA, mdtA, aph(2')-Id-01, blaSHV-02,blaAmpC-blaDHA, catA1, blaNDM-1, blaCTX-M-06, acrA-02, aacA-aphD, acrR-02, ermB, ermT-02, acrB-01, yceL-mdtH-03, yceL-mdtH-02, yceE-mdtG-01, yidY-mdtL-02, blaCTX-M-02, aacC2, blaPER, ermT-01, aph(2')-Id-02, and rarD-02, vanB-01, blaPSE, blaTEM, pikR2, tetR-02, tetG-02, tetG-01, sul2, tetJ(32), tetX, tetQ, tetM-01, tetM-02, tetPB-03, and tetA-02.*	HT-qPCR/Illumina HiSeq 2500	Aminoglycoside, beta-lactamase, MLSB, and multidrug genes such as *blaNDM-1, blaCTX-M* variants, *ermB*, and *acrA/acrB* efflux genes are strongly enriched in WWTP influent and effluent samples but show very low abundance in reservoirs and ponds, reflecting the heavy, clinically relevant ARG load carried by raw and even treated sewage. *vanB-01, blaPSE, blaTEM*, and *pikR2* are enriched specifically in WWTP effluent samples, marking these as distinct indicator genes for treated wastewater discharge, including a vancomycin-resistance signal not prominent elsewhere. Tetracycline-resistance genes like *tetA, tetM, tetQ, tetX, tetG*, and *tetPB*, along with *sul2*, show high abundance in reservoirs and ponds but low abundance in WWTP samples, suggesting that tetracycline and sulfonamide resistance genes are the characteristic "indicator" ARGs of these standing urban/drinking water bodies rather than being sewage-dominated genes.	[229]
80.	March – September 2014	Water samples (rivers, lakes, streams, ponds)	Tanzania: Longido, Monduli, Simanjiro, Moshi, Arumeru, Arusha	*blaTEM-1, blaSHV-1, blaCTX-M, CTX-M-1, CTX-M-79, CTX-M-9, tet(A), tet(B)*	Culture-based method/Standard PCR	Among the 143 ampicillin-resistant isolates, *blaTEM-1* was the most frequent gene (67%), followed by *blaCTX-M* (17.7%) and *blaSHV-1* (6.0%). Notably, while the broader *blaCTX-M* category showed no significant distribution bias, all 16 *blaCTX-M*-positive isolates originated exclusively from open water sources. Sequencing confirmed 15 of these as *blaCTX-M-15* and one as *blaCTX-M-79*. In the 364 tetracycline-resistant isolates, *tet(A)* was the dominant genotype (57.4%), followed by *tet(B)* (24.0%).	[230]
81.	May 2018 – April 2019	WWTP influent, effluent, and sewage sludge samples	Germany	*blaOXA-58, blaOXA-23, blaOXA-48,*<br>*blaGES, blaVIM, blaCTX-M-1, blaCTX-M-9, blaCMY-2, blaFOX, blaACT-MIR,*	RT-qPCR	This study identified a diverse range of resistance determinants within a wastewater treatment plant, detecting 11 of the 13 screened beta-lactamase gene types, most notably *blaGES*, which was the most frequent, followed by *blaOXA-58* and *blaFOX*, alongside the presence of mcr-1 in 67% of wastewater samples. While *blaKPC, blaNDM*, and *mcr-2* were not detected, most other genes	[231]

				blaDHA, mcr-1, mcr-2		were found across all sample types, with the specific exception of *blaOXA-23, blaCTX-M-9*, and *mcr-1*, which exhibited more restricted distributions.	
82.	October 2014 – January 2015	Sediments of polluted wetlands	Nigeria: Lagos, Ibadan	*blaCTX-M, blaCTX-M-1, blaAmpC*	RT-qPCR	The study identified significant AMR contamination in the Awba (AW) wetland, where the *blaCTX-M* and *blaAmpC* genes were the only clinically relevant determinants detected among those screened. *blaAmpC* was consistently present in November 2014 and January 2015, with normalised abundances significantly higher than those reported in other areas. While reliable quantification of *blaCTX-M-1* was limited to January 2015 due to technical constraints, successful bacterial isolations and PCR detections confirmed its persistent presence throughout the sampling period. Notably, the occurrence of both *blaCTX-M-1* and *blaAmpC* correlated strongly with the presence of *E. coli*, which was exclusively found at the AW site, highlighting the wetland as a substantial reservoir for clinically significant antibiotic resistance.	[232]
83.	February 2021	Lerma River water samples<br>Lake Chapala water samples	Mexico	*blaCTX, blaTEM, blaSHV, blaOXA, blaNDM, blaKPC, blaVIM, qnrA, qnrB, qnrS, aac(6')Ib-cr*	Culture-based method/Standard PCR/Illumina NextSeq 550	The 59 MDR Gram-negative isolates from the Lerma River basin revealed a high prevalence of resistance genes, with *blaCTX-M* being the most abundant β-lactamase gene (88%), followed by *blaSHV* (10%), *blaOXA* (7%), *blaTEM* (5%), and *blaVIM* (3%). Phenotypic resistance to β-lactams was high, particularly for cefotaxime and ceftazidime. Fluoroquinolone resistance genes *qnrB* (51%) and *qnrS* (14%) were also frequently detected, while the aminoglycoside resistance gene *aac(6')lb-cr* was identified in 10% of isolates. Whole-genome sequencing of selected *E. coli*, *C. freundii*, and *K. pneumoniae* strains uncovered a higher spectrum of resistance determinants, including genes for resistance to sulfonamides (*sul1, sul2*), tetracyclines (*tet(A-C)*),	[233]

						and other classes (e.g., *ampH, blaCMY-70, blaDHA-1, fosA*).	
84.	May – November 2018	WWTP upstream, downstream, influent and effluent water samples	South Africa	*blaTEM-1B, blaCTX-M-14, blaCTX-M-55, aph(3")-Ib, aph(6)-Id, aadA1, aadA5, tet(A), tet(M), tet(B), sul1, sul2, dfrA1, dfrA14, dfrA17, mdf(A), mph(A), mcr-9, qnrB19, gyrA, gyrB, parE*	Culture-based method/Illumina MiSeq	All *E. coli* isolates harboured ARGs showed multiple resistance classes: β-lactamases, *blaTEM-1B, blaCTX-M-14, blaCTX-M-55*, aminoglycosides, *aph(3")-Ib, aph(6)-Id, aadA1, aadA5*, tetracyclines, *tet(A), tet(M), tet(B),* sulfonamides, *sul1, sul2*, trimethoprim, *dfrA1, dfrA14, dfrA17*, macrolides, *mdf(A), mph(A),* colistin, *mcr-9*, and quinolones, *qnrB19*. The predominant β-lactamase was *blaTEM*-1B (58.3% of isolates), with *blaCTX-M-14* and *blaCTX-M-55* co-detected in a single novel ESBL-positive isolate (D106). The *mcr-9* and *qnrB19* genes represented notable findings associated with colistin and plasmid-mediated quinolone resistance, respectively.	[234]
85.	October 2016	2 WWTP water samples<br>One Svratka River sample<br>3 hospital inflow, outflow, and sewage samples	Czech Republic: Brno city	*blaCTX-M-15, blaCTX-M-14, blaCTX-M-1, blaCTX-M-27, blaCTX-M-3, blaCTX-M-32, blaCTX-M-174, blaCMY-2, blaDHA-1, mph(A), erm(B), mdf(A), gyrA, parC, parE.*	Culture-based method/Multiplex PCR/Illumina MiSeq 4000	Among the 78 sequenced *E. coli* isolates, ESBL production was predominantly encoded by *blaCTX-M* variants, *blaCTX-M-15, blaCTX-M-14, blaCTX-M-1, blaCTX-M-27, blaCTX-M-3, blaCTX-M-32, blaCTX-M-174*, with *blaCTX-M-15* accounting for the majority (62.8%), alongside *blaAmpC* genes *blaCMY-2 and blaDHA-1*; macrolide resistance was mainly conferred *by mph(A) and erm(B),* with the intrinsic *mdf(A*) gene present in nearly all isolates (96.2%); and fluoroquinolone resistance was mediated through cumulative chromosomal mutations in *gyrA, parC*, and *parE*, with all isolates harbouring three or more mutations exhibiting full phenotypic resistance to nalidixic acid and ciprofloxacin.	[235]
86.	*N.R.*	WWTP influent and effluent samples	Morocco: Marrakech	*blaCTX-M, blaTEM, blaSHV*	Standard PCR	*blaCTX*-M was the most frequently detected gene, present in 100% of isolates across all sites. *blaTEM* showed high prevalence in all the samples. *blaSHV* was less frequent but consistently detectable across all sampling sites.	[236]

						Notably, co-occurrence of all three genes was observed in all isolates.	
87.	March 2022	Upper, middle and low reaches of Tipitapa River samples	Nicaragua	*bla-SHV, blaSHV-13, blaSHV-24, bla-TEM, blaTEM-1, blaTEM-116, bla-CTX-M, bla-OXA*	Culture-based method/Multiplex PCR	ESBL-encoding genes were detected in 84% (21/25) of Gram-negative isolates, with *blaSHV* (80.95%) and *blaTEM* (42.85%) being the predominant resistance genes, co-occurring in 23.80% of isolates. Sequencing analysis identified four specific gene variants: *blaSHV-24* was the most prevalent, detected in 47.62% of isolates (n=10), followed by co-carriage of *blaSHV-24* and *blaTEM-1* in 23.81% (n=5), *blaTEM-1* alone in 14.28% (n=3), *blaSHV-13* in 9.52% (n=2), and *blaTEM-116* in a single isolate (4.76%), distributed across *Aeromonas, Acinetobacter, Enterobacter, Klebsiella, Escherichia, Providencia*, and *Vibrio* species.	[237]
88.	2017 – 2019	WWTP samples	Tunisia: Tunis, La Charguia	*blaCTX-M-15, blaCTX-M-27, blaCTX-M-55, blaSHV-12, blaTEM, blaSHV, tet(A), tet(B), sul1, sul2, sul3, aac(6')-Ib-cr, aac(3)-II, cmlA, floR, cat, mcr-1*	Standard PCR	Among the 57 ESBL-producing isolates, all but one, *E. cloacae* complex, which carried *blaSHV-12*, harboured a *blaCTX-M* gene, with *blaCTX-M-15* predominating (92.9%, n=52), followed by *blaCTX-M-27* (5.4%, n=3) and *blaCTX-M-55* (1.8%, n=1); *blaCTX-M-15* frequently co-occurred with *blaTEM* (13 *E. coli*, 1 *C. freundii*), *blaSHV* (1 *K. pneumoniae*), or both *blaTEM* and *blaSHV* (6 *K. pneumoniae*). Beyond β-lactams, tetracycline resistance was mediated by *tet(A)* (78.6%) and *tet(B)* (21.4%); sulfonamide resistance by *sul2* (62.9%), *sul1* (31.4%), and *sul1+sul3* (5.7%); fluoroquinolone resistance by *aac(6')-Ib-cr* (in 2 of 13 ciprofloxacin-resistant isolates); aminoglycoside resistance by *aac(3)-II* (n=7); and phenicol resistance by *cmlA* (n=2), *floR* (n=2), and *cat* (n=2). Among seven colistin-resistant isolates, only one *E. coli* harboured the plasmid-mediated *mcr-1* gene.	[238]
89.	*N.R.*	WWTP influent and	South Africa: Durban	*blaCTX-M, blaSHV, blaTEM*	RT-qPCR	Among the 39 ESBL-positive *E. coli* isolates, 77% (n=30) harboured ESBL antibiotic resistance genes (ARGs), with a higher prevalence in	[239]

		effluent samples				effluent samples (n=19) than in influent samples (n=11). *blaCTX-M* was the most frequently detected type (67%; n=20), followed by *blaTEM* (57%; n=17) and *blaSHV* (27%; n=8). Regarding co-occurrence, 13% of isolates carried all three types, while 13% co-carried *blaCTX-M* and *blaSHV*, and 10% co-carried *blaCTX-M* and *blaTEM*. Single-gene detection was observed in 30% of isolates for *blaCTX-M* and 33% for *blaTEM*, whereas no isolates carried only the *blaSHV* gene.	
90.	December 2024 – February 2025	Household wastewater samples	Nigeria: Gombe State	*blaTEM, blaSHV, blaCTX-M,, blaVIM, blaNDM, blaKPC*	Culture-based method/Standa rd PCR	PCR screening of ESBL genes among ten ESBL-producing Enterobacteriaceae isolates revealed *blaCTX-M* in all ten isolates, *blaTEM* in nine (90%), and *blaSHV* in six (60%), confirming *blaCTX-M* as the dominant ESBL gene in the study area. Among five carbapenem-resistant *P. aeruginosa* isolates screened for carbapenemase genes, *blaVIM* was detected in three (60%) and *blaKPC* in one (20%), while *blaNDM* was not detected.	[240]
91.	March – April 2019<br>June – August2019	Water samples from two distinct rivers<br>Hospital wastewater and sediments	Democratic Republic of Congo: Kinshasa	*blaCTX-M, blaOXA-48, blaIMP, blaTEM*	qPCR	*blaCTX-M* was abundant across all locations in both seasons, generally among the highest of the four genes, with somewhat more variable levels in the wet season. *blaIMP* also showed high relative abundance overall, peaking at the hospital site in both seasons and remaining notably elevated at the clinic in the wet season. In contrast, *blaOXA* consistently showed the lowest relative abundance of the four genes, dropping further in the wet season to below 10^{-6} copies/16S at all four locations. *blaTEM* maintained a relatively stable, intermediate abundance across locations and seasons, without the extreme peaks or drops seen in the other genes.	[241]
92.	June 2016	Svartån river water samples	Sweden: Örebro city	*blaCTX-M-1, blaSHV-156G, blaSHV-238G240E, blaACT-1,*	Culture-based method/qPCR	Analysis of 84 ARGs across hospital wastewater, WWTP, and environmental water samples detected 55 ARGs overall, with 18 of these (33%)	[242]

		Hjälmaren lake water samples<br>WWTP influent and effluent samples<br>Hospital wastewater samples		*blaACT-5/7, blaCMY-10, blaDHA, blaFOX, blaLAT, blaMIR, blaMOX, qnrB-1, qnrB-5, qnrB-8, tetA, tetB, aacC2, aadA1, blaIMP-1, blaIMP-2, blaIMP-5, blaIMP-12, blaVIM-1, blaVIM-13, blaOXA-23, aphA, vanC*		— including class A β-lactamases, *blaCTX-M-1, blaSHV-156G, blaSHV-238G240E*, class C β-lactamases, *blaACT-1, blaACT-5/7, blaCMY-10, blaDHA, blaFOX, blaLAT, blaMIR, blaMOX*, fluoroquinolone resistance genes, *qnrB-1, qnrB-5, qnrB-8*, tetracycline resistance genes, *tetA, tetB*, and aminoglycoside resistance genes, *aacC2, aadA1,* found to be widespread across every site. The clinically important class B β-lactamase carbapenemase genes, *blaIMP-1, blaIMP-2, blaIMP-5, blaIMP-12, blaVIM-1, blaVIM-13*, were concentrated almost exclusively in hospital wastewater, which also carried unique genes not found elsewhere, including additional carbapenemases, *blaIMP-1, blaIMP-2, blaOXA-23*, the aminoglycoside resistance gene *aphA*, and the vancomycin resistance gene *vanC*, marking hospital wastewater as a reservoir of particularly diverse and clinically significant ARGs.	
93.	September – November 2017	160 tap water samples<br>120 house tanks samples	Sudan: Khartoum State	*blaCTX-M-1*	Culture-based method/ Standard PCR	Of the 86 *E. coli* isolates analysed for *blaCTX-M-1* group detection, 40% (34/86) were positive, with prevalence varying by location: 31% (13/42) in Khartoum, 55% (11/20) in Khartoum North, and 42% (10/24) in Omdurman. Among the 34 *blaCTX*-M-1-positive isolates, 38% (18/47) were recovered from tap water and 41% (16/39) from tank water.	[243]
94.	February – June 2015	Drinking water samples	Jordan	*blaCTX, blaTEM, blaNDM, blaKPC, blaSHV*	Culture-based method/Multiplex PCR	Among the 109 isolates examined, β-lactamase genes were identified at varying frequencies, with *blaVIM* being the most prevalent (68.8%; 75/109), followed by *blaCTX* (50.5%; 55/109), *blaTEM* (45.9%; 50/109), *blaNDM* (11%; 12/109), *blaKPC* (4.6%; 5/109), and *blaSHV*, which was detected in only a single isolate (0.9%; 1/109).	[244]
95.	Mat – July 2017	9 WWTP influent and effluent samples	South Africa: Durban	*blaCTX-M, blaTEM, blaSHV*	Multiplex PCR	Among the 140 isolates, 40 (28.6%) carried both *blaTEM* and *blaCTX-M*, whereas *blaSHV* was not detected at all.	[245]

		6 surface final effluent samples					
96.	November 2016 – January 2017	WWTP influent and effluent samples	Portugal: North Lisboa, Algarve	*blaCTX-M-1, blaCTX-M-2, blaCTX-M-14, blaCTX-M-15, blaCTX-M-27, blaCTX-M-32, blaCTX-M-174, blaKPC-3, blaOXA-2, blaSED, aac(6)-Ib-cr5, aadA1,aadA2,*<br><br>*aadA5, dfrA1, dfrA12, dfrA15, dfrA17,*	Culture-based method/Standa rd PCR/Illumina Platform PE150	Among ESBL-encoding genes, *blaCTX-M-15* was the most common (n = 37), followed by *blaCTX-M-14* (n = 8) and *blaCTX-M-27* (n = 4), while *blaCTX-M-1, blaCTX-M-2, blaCTX-M-32*, and *blaCTX-M-174* were each found in a single isolate; *blaSED-1* was detected in *Citrobacter farmeri* and *blaCTX-M-15* in *Ochrobactrum intermedium*, with these ESBL genes conferring resistance to third-generation cephalosporins and being widespread among Enterobacteriaceae. Among carbapenemase genes, *blaKPC-3* was identified in one *K. oxytoca* isolate, while *Uruburuella suis* carried the narrow-spectrum *blaOXA-2* gene. Fluoroquinolone resistance was mediated by *aac(6')-Ib-cr5*, found in 26 isolates, 17 of which also carried *blaCTX-M-15*. Aminoglycoside resistance genes, *aadA1, aadA2, aadA5*, and trimethoprim resistance genes, *dfrA1, dfrA12, dfrA15, dfrA17*, were the most prevalent co-occurring genes, associated with class 1 integrons detected in 49 resistant wastewater isolates.	[246]
97.	May 2018	WWTP influent samples	South Korea: Seoul, Han River	*blaNDM-5*	Culture-based method/Standa rd PCR/PacBio sequencing	Of the 50 isolates, only a single isolate tested positive for carrying the *blaNDM-5* gene.	[247]
98.	December 2017 – January 2018<br><br>June – July 2018	Surface sediment of hospital effluents<br><br>River sediment samples	Democratic Republic of Congo: Kinshasa	*blaSHV,*<br><br>*blaCTX-M, blaNDM, blaKPC, blaOXA-48, blaVIM, blaIMP*	qPCR	All of the genes analysed were detected in substantial quantities across sampling sites, except *blaNDM*. Before the rain event, most genes showed no significant enrichment along the river, except for *blaCTX-M* and *blaIMP*, which were notably enriched near the hospital effluent discharge point. After the rain event, only *blaOXA-48* exhibited significant enrichment along	[248]

						the river, while the remaining genes, *blaCTX-M, blaIMP, blaKPC, blaNDM, blaSHV, and blaVIM*, showed no selective enrichment.	
99.	October, December, and March 2019	Sewage upstream and downstream water samples<br>Soil samples along the Min-xin River	China: Shijiazhuang	*tetC, tetO, sul1, sul2, sul3, ermA, ermB, blaOXA-10, blaSHV, blaCTX-M,blaAmpC*	qPCR	In samples collected across the study period, several antibiotic resistance genes consistently showed the highest absolute abundances and significant spatial variation. In October, *sul1* and *blaSHV* were the most abundant genes in water and soil, respectively, with sul1 and *blaOXA-10* showing significantly higher abundance at points P3 and P4 compared to P1 in water. In December, *sul1* dominated in water samples while *sul2* dominated in soil samples, with *tetC, sul1, sul2, sul3, blaOXA-10, blaSHV*, and *blaAmpC*, all showing significantly higher abundance at points P2, P3, and P4 relative to P1. By March, *blaSHV* had become the most abundant gene in both water and soil samples, with *tetC, sul1, sul2, blaSHV, blaOXA-10*, and *int1* again showing significantly elevated levels at P2, P3, and P4 compared to P1.	[249]
100.	March 2018 – February 2019	Sewage water samples	India: Delhi	*blaNDM-1*	Culture-based method/Standa rd PCR	The analysis of the 138 organisms for *Int1* and *blaNDM-1* genes revealed that 89.9% (124/138) carried at least one of these genes, while 10.1% (14/138) carried neither; specifically, 18.4% (26/138) carried only *int1*, 17.4% (24/138) carried only *blaNDM-1*, and 53.6% (74/138) were positive for both genes. Among the 98 *blaNDM-1*-positive, genotypically resistant organisms, 52 (37.7% of 138) originated from the untreated influent stream (66.7% of 78 influent isolates) and 46 (33.8% of 138) from the treated effluent stream (76.7% of 60 effluent isolates), reflecting a 10% increase in genotypic resistance in the outgoing stream that was observed across all organisms except *Escherichia spp.* Similarly, of the 74 double-gene-positive organisms (53.6% of 138, carrying both *blaNDM-1* and *Int-1*, 38 (27.5% of 138) were from the incoming stream (48.7% of	[250]

						78) and 36 (26.1% of 138) from the outgoing stream (60% of 60).	
101.	September 2019 – March 2020	Wastewater samples from Kaptan Bazar Slaughterhouse	Bangladesh: Dhaka	*dfrA1, dfrA17, blaOXA-48, tetA, blaOXA-47, blaTEM, blaNDM-1, blaKPC-1, blaKPC-2, blaKPC-3, blaVIM-1, blaVIM-2, mcr-1, mcr-2, mcr-3, mcr-4, mcr-5, sul1, sul2*	Culture-based method/Standard PCR	Among the resistance determinants examined, *dfrA17* was the most frequently detected, found in 25.46% of isolates, followed by *blaOXA-48* (21.76%) and *tetA* (13.89%), while other genes occurred at lower frequencies, ranging from 0.46% for *blaOXA-47* to for 6.94% *blaTEM*. None of the isolates carried *blaNDM-1, blaKPC-1, blaKPC-2, blaKPC-3, blaVIM-1, blaVIM-2*, or any of the *mcr* genes.	[251]
102.	July 2022 – April 2023	18 river and stream water samples<br>48 soil samples	Portugal	*blaCTX-M, tetA, sul2, qnrB, blaCTX-M-15, blaTEM-1, blaSHV-28, blaOXA-1, blaAmpC qnrB1, oqxA, oqxB19, aac(6')-Ibcr, sul2, dfrA14, tetA, aph(6)-Id, aph(3")-Ib, acrD, catB3, dfrA14*	Culture-based method/Standard PCR/Illumina MiSeq	Initially, a PCR analysis showed the *blaCTX-M*-1, *tetA, sul2,* and *qnrB* resistance genes, along with the IntI1 integrase responsible for mobilising resistance cassettes within integrons. WGS confirmed all PCR-detected genes and revealed further genomic detail, identifying the isolate as sequence type ST307 and harbouring multiple β-lactamase genes, including *blaCTX-M-15, blaTEM-1, blaSHV-28*, and *blaOXA-1*. Additional resistance genes detected included *qnrB1, oqxA, oqxB19, and aac(6')-Ib-cr*, conferring quinolone resistance; *sul2* and *dfrA14*, conferring sulfonamide and trimethoprim resistance; *tetA*, conferring tetracycline resistance; *aph(6)-Id, aph(3")-Ib*, and *acrD*, conferring aminoglycoside resistance; and *catB3*, conferring chloramphenicol resistance—although no phenotypic resistance to these latter drug classes was observed in this study.	[252]
103.	March – May 2018	Urban landscape water samples	China: Xi'an	*tetA, tetB, tetC, tetG, tetJ, tetK, tetM, tetO,*<br>*tetQ, tetX, sulI, sulII,*<br>*sulIII, blaampC, blaCTX-M,*	RT-qPCR/Illumina MiSeq	Sulfonamide resistance genes showed the highest overall relative abundance, significantly exceeding other gene classes, with *sulI* and *sulIII* being the most abundant genes detected overall. Among tetracycline resistance genes, *tetG* and *tetX* were the most widely distributed, while *tetB*, though detected in all samples, occurred at lower abundance. The remaining tetracycline genes were	[253]

				blaSHV, blaTEM, qnrA		rarely found. Of the β-lactam resistance genes, *blaAmpC* was the most abundant, with notably higher levels in reclaimed water (RW)-replenished waters compared to surface water (SW)-replenished waters, while *blaCTX-M*, *blaSHV*, and *blaTEM* showed similar abundances across all sites. Among quinolone resistance genes, *qnrA* occurred at low overall abundance but was significantly enriched in RW-replenished waters relative to SW-replenished waters.	
104.	February 2021 – January 2022	WWTP influent samples	Finland	*blaCTX-M-15, blaCTX-M-27, blaCTX-M-14, blaCTX-M-55, blaCTX-M-1, blaCTX-M-2, blaTEM-1, blaOXA-1, blaSHV-12, blaKPC-2, blaNDM-1, aac(6')-Ib-cr5, aadA1, aadA5, aph(3")-Ib, aph(6)-Id, qnrS1, gyrA_S83L, gyrA_D87N, parC_S80I, parC_E84V, parE_I529L, sul1, sul2, tet(A), tet(B), dfrA14, dfrA17, mphA, mcr-1.1, marR_S3N, uhpT_E350Q, ptsI_V25I*	Culture-based method/Illumina HiSeq PE150	Among the 73 isolates, *blaCTX-M* genes were highly prevalent (86.3%), frequently co-occurring with *blaTEM-1* and the blaAmpC-associated *blaOXA-1*, with *blaCTX-M-15* as the dominant variant, followed by *blaCTX-M-27*, while *blaCTX-M-14, blaCTX-M-55, blaCTX-M-1, blaCTX-M-2*, and *blaSHV-12* were detected less frequently; carbapenemase genes *blaKPC-2* and *blaNDM-1* were each found in only a single isolate. Fluoroquinolone resistance was widespread (82.2%), driven mainly by chromosomal mutations in *gyrA* and *parC/parE* rather than the plasmid-mediated qnrS1 gene. Other commonly detected resistance genes included the aminoglycoside genes *aac(6')-Ib-cr5, aadA1, aadA5, aph(3")-Ib*, and *aph(6)-Id*; the sulfonamide genes *sul1* and *sul2*; the tetracycline genes *tet(A)* and *tet(B);* the trimethoprim genes *dfrA14* and *dfrA17*; and the macrolide gene *mphA*. Colistin resistance, mediated by *mcr-1.1*, was identified in only one isolate and point mutations linked to chloramphenicol/rifampin resistance, *marR_S3N*, and fosfomycin resistance, *uhpT_E350Q, ptsI_V25I*, were also detected in a subset of isolates.	[254]
105.	*N.R.*	4 tap water samples	India: Telangana	*blaOXA-48, blaNDM, blaKPC, blaVIM, blaIMP-1*	Culture-based method/RT-qPCR	All the environmental samples were positive for ESBL and carbapenemase-producing bacteria, predominantly Enterobacteriaceae but also including non-fermenters, with *blaOXA-48* detected most frequently (22 samples), followed	[255]

		1 borehole water sample<br>23 environmental samples				by *blaNDM* (10), *blaKPC* (7), *blaVIM* (5), and *blaIMP-1* (5); notably, two samples, one from the Musi River, tested positive for all carbapenemase genes assessed. Among the samples collected near bulk drug manufacturing plants, *blaOXA-48* was again the predominant gene, followed by *blaNDM* and *blaKPC*. Despite this widespread contamination, two samples, both tap water from a four-star hotel in Banjara Hills, Hyderabad, tested negative for all MDR pathogens. Tap water from a food stall in Dundigal contained ESBL-producing Enterobacteriaceae and non-fermenting bacteria, including species positive for *blaOXA-48*, while a borehole sample from Dundigal and tap water from the village of Isnapur yielded Enterobacteriaceae and/or non-fermenters without evidence of multidrug resistance.	
106.	February – June 2024	16 Hospital WWTP influent and effluent samples	Cameroon	*blaCTX-M, blaTEM, blaSHV, aac-(6')-Ib-cr, oqxA, qnrB. qnrS*	Culture-based method/Standard PCR	Among the isolates tested, β-lactamase genes were common overall, with *blaCTX-M* as the most prevalent, showing an even higher detection rate in wastewater samples (85.0%, n = 68/80) compared to clinical samples (73.7%, n = 42/57), followed globally by *blaTEM* (58.79%) and *blaSHV* (28.57%), with *blaCTX-M* + *blaTEM* being the most frequent gene combination overall (36.26%). Among PMQR genes, *aac-(6')-Ib-cr* was the most frequently detected (61.88%), followed by *oqxA* (37.50%) and *qnrB* (30.00%), *with aac-(6')-Ib-cr, qnrB,* and *qnrS* being the predominant PMQR genes.	[256]
107.	November 2023	Surface water samples	South Africa: Durban, Pietermaritzburg	*blaTEM-1, sul1, sul3, dfrA12, qnrB19, qnrS1, tet(A), cmlA1, aadA1 and aadA2. tet(M), tet(L), msr(C), erm(B)*	Culture-based method/Illumina MiSeq	The study identified a range of ARGs across both *E. coli* and *E. faecium* isolates recovered from surface water. Cotrimoxazole resistance was associated with the presence of *sul1, sul2, sul3, dfrA12*, and *dfrA17* genes. In *E. faecium*, erythromycin resistance was mediated by *erm(B),* detected in two isolates, and the macrolide efflux pump gene *msr(C),* detected in all isolates, while the *efmA* efflux pump determinant conferred additional multidrug resistance, including to	[257]

						macrolides. Tetracycline resistance was conferred by two genes, *tet(M)* and *tet(L),* which functioned through distinct mechanisms and were detected across isolates and correlated with observed phenotypic tetracycline resistance. *blaTEM-1B* was only identified in the *E. coli* isolates.	
108.	2016 – 2023	34 urban water samples from Córdoba city<br>22 surface water samples from the Suquía River<br>6 surface water samples from Ansenuza National Park	Argentina: Córdoba	*blaCTX-M, blaKPC-2, blaNDM-1*	Culture-based method/Standard PCR/Illumina MiSeq	The predominant genetic determinant of ESBL production was *blaCTX-M*, while among carbapenemase-producing strains, *blaKPC* was detected in five isolates and *blaNDM* in one. ESBL-producing *E. coli* was the most prevalent resistant phenotype, accounting for 66% of isolates in wastewater and 62% in the Suquía River	[258]
109.	August – September 2022	8 water samples from the Belize River watershed<br>3 water samples from the coast<br>2 water samples	Belize	*sul1, sul2, ermF, tetA, blaSHV*	qPCR/Illumina Nova Seq 6000	Across 18 water samples. Spatially*, sul1, intI1*, and *sul2* clustered together. At the same time, *ermF* and *tetA* showed similar trends, with the highest gene abundances recorded at sites along Haulover Creek in Belize City, its coastal pourpoint, and near the treatment lagoon discharge point. Intermediate abundances were observed at inland Belize River sites and upstream Haulover Creek locations, while the lowest concentrations were found at all Glover's Reef Atoll sites and Gallow's Point Reef. Overall, ARG concentrations in Belize ranged from 2.62×10^{-3} to 5.42×10^{3} copies/L — significantly lower than comparable studies. Notably, while *sul1* values in	[259]

		from the coral areas<br>5 water samples fromglover's Reef Atoll				Belize were approximately three orders of magnitude lower than those in other studies, *sul2* concentrations were comparatively higher.	
110.	November 2020 – July 2023	Wwyps influent and effluent samples	Spain: Catalonia, Osona	*blaCTX-M-15, blaCTX-M-14, blaCTX-M-1, blaCTX-M-27, blaCTX-M-32, blaCTX-M-42, blaCTX-M-55, blaTEM-1B, blaTEM-30, blaTEM-141, blaTEM-206, blaTEM-207, blaSHV-12, blaOXA-1, qnrS1, qepA4, aac(6')-Ib-cr, aac(3)-IV, aac(3)-IId, aac(3)-IIa, sul1, sul2, dfrA1, dfrA12, dfrA14, dfrA17, tet(A), tet(B)*	Culture-based method/Illumina MiSeq	ARG analysis revealed that β-lactam resistance was predominantly mediated by *blaCTX-M-15* (24%) and *blaTEM-1B* (20%), followed by *blaCTX-M-14* and *blaCTX-M-1* (8% each), while *blaCTX-M-27, blaCTX-M-32, blaCTX-M-42, blaCTX-M-55, blaSHV-12, blaTEM-30, blaTEM-141, blaTEM-206, blaTEM-207,* and *blaOXA-1* were each detected in a single isolate (4%). Fluoroquinolone resistance was primarily associated with *qnrS1* (66.6%), with *aac(6')-Ib-cr* and *qepA4* each detected in one isolate; aminoglycoside resistance was mediated by *aac(3)-IV* (40%), *aac(3)-IId, aac(3)-IIa* combined with *aac(6')-Ib-cr*, and *aac(3)-IIa* alone (20% each). Trimethoprim/sulfamethoxazole resistance involved *sul2* alone in 2 isolates, *sul1 + sul2* in 1 isolate, *dfrA14* and *dfrA17* in 2 isolates each, and *dfrA1* and *dfrA12* in 1 isolate each. Tetracycline resistance was conferred by *tet(A)* in 7 isolates and *tet(B)* in 2 isolates.	[260]
111.	October 2017 – May 2019	Hospital wastewater samples	Peru: Lima	*blaCTX-M-14, blaCTX-M-15, aac3-IId, mcr-1*	Culture-based method/Illumina MiSeq	The distribution of ARGs differed between clinical and wastewater isolates, with wastewater samples exhibiting a greater diversity of ARGs across *E. coli* and *Klebsiella spp*., encompassing β-lactam resistance genes, *blaCTX-M-14* and *blaCTX-M-15*, aminoglycoside resistance, *aac3-IId*, and the colistin resistance gene *mcr-1*.	[261]
112.	September 2014	Surface water samples from the	South Korea: Gwangju City	*blaNDM-9, aadA2, ant(30)-Ia and aph(30)-Ia, blaCTX-M-65, blaLEN-13, blaTEM-1B,*	WGS with PacBio system	A total of 13 ARGs were identified, spanning seven antibiotic resistance classes: aminoglycoside resistance *aadA2, ant(3")-Ia, aph(3")-Ia*, class A β-lactamases *blaCTX-M-65, blaLEN-13, blaTEM-1B*, class B β-lactamase	[262]

		Yeongsan River Basin		*oqxA, oqxB, dfrA12, sul1, fosA, mph(A)*		*blaNDM-9*, fluoroquinolone and quinolone resistance *oqxA, oqxB*, folate pathway inhibitors *dfrA12, sul1*, fosfomycin resistance *fosA*, and macrolide resistance *mph(A)*.	
113.	October 2022 – March 2023	58 samples from WWTP<br>63 raw wastewater samples from a university campus	Canada: Ontario	*blaGES, blaOXA-48, and blaIMP-27*	RT-qPCR	Gene concentrations varied across sites. At WWTP Site 1, *blaGES* ranged from 5.2×10^5 to 9.2×10^6 copies/mL, *blaIMP-27* from 0 to 1.4×10^6, and *blaOXA-48* from 0 to 4.1×10^6. At the university campus, *blaGES* ranged from 2.9×10^5 to 1.7×10^7, *blaIMP-27* from 0 to 1.9×10^6, and *blaOXA-48* from 0 to 1.1×10^7. At WWTP Site 2, *blaGES* ranged from 1.4×10^6 to 2.4×10^7, *blaIMP-27* from 0 to 4.2×10^6, and *blaOXA-48* from 0 to 1.2×10^4. Statistically significant differences were observed between genes at all three sites, except between *blaIMP-27* and *blaOXA-48*; after normalization to site population, WWTP Site 2 was significantly different from both the university campus and WWTP Site 1 for all genes except *blaIMP-27*.	[263]
114.	May – June 2017	Wastewater samples<br>Creek water samples	Italy: Abruzzo region	*blaCTX-M-15, blaCTX-M-27, blaOXA-1, blaOXA-2, blaOXA-11,*<br>*blaOXA-23, blaAmpC, blaCphA*	Standard PCR	All 30 analysed strains were confirmed to carry β-lactamases, with the *blaAmpC* gene detected in all isolates except those of *A. hydrophila* and *A. veronii*. Plasmid-mediated *blaCTX-M-15* was identified in *E. coli*, *R. ornithinolytca*, *C. freundii*, and *E. cloacae*, while *blaCTX-M-27* was found in *S. liquefaciens*. In contrast, the chromosome of *A. hydrophila* and *A. veronii* exclusively harbours the *blaCphA* gene, which encodes a subclass B2 metallo-β-lactamase. Furthermore, while P. koreensis tested negative for the *blaOXA-1, blaOXA-2, blaOXA-11*, and *blaOXA-23* variants. However, the presence of an undetermined *blaOXA*-type enzyme is suspected due to the inherent heterogeneity of this group.	[264]
115.	May 2024 – December 2024	Water samples from	Italy: Apulia region	*blaNDM, blaKPC, blaOXA-48, blaVIM, blaIMP, blaCTX-M*	Culture-based method/RT-	Of the 176 isolates screened, 121 (68.8%) tested positive for at least one resistance gene, with the *blaCTX-M* being the most prevalent, identified in	[265]

		surface water bodies			qPCR/Illumina NextSeq	79.3% (96/121) of these positive isolates either independently or alongside carbapenemase genes. Carbapenemase-encoding genes were detected in 20.6% (25/121) of the positive samples, specifically including *blaVIM* (9.1%, 11/121), *blaOXA-48* (3.3%, 4/121), and *blaKPC* (1.7%, 2/121), while the remaining 31.2% (55/176) of the total isolates carried none of the investigated resistance targets.	
116.	August – September 2024	Water samples from Laguna Lake	Philippines: West Bay	*blaTEM-1, blaTEM-117, blaTEM-162, blaTEM-187, blaTEM-192, blaTEM-193, bacA, catI, epytA, mdL, mdbA*	Illumina NovaSeq	The ARG subtype distribution reveals varied levels of diversity across resistance classes. The β-lactam category shows high diversity, characterised by *blaTEM-1* (21.8%), *blaTEM-117* (18.8%), and several smaller percentages of other *blaTEM* variants (*blaTEM-162, blaTEM-187, blaTEM-192*, and *blaTEM-193*) alongside other subtypes (29.9%). In contrast, other classes demonstrate higher uniformity, with *bacA* (90.6%) dominating bacitracin resistance, *catI* (93.0%) representing the majority of chloramphenicol resistance, and *eptA* (71.2%) being the primary subtype for polymyxin. Multidrug and tetracycline resistance are largely composed of other subtypes, accounting for 65.9% and 87.2% of their respective distributions, with the multidrug category additionally including specific contributions from *mdL* (13.6%) and *msbA* (6.6%)	[266]
117.	*N.R.*	ETP samples from pharmaceutical manufacturers<br>Environmental water samples	Bangladesh: Dhaka	*blaNDM, blaCTX-M-1, blaTEM, blaSHV-1, blaVIM-1*	Culture-based method/Enterobacterial repetitive intergenic consensus sequence–PCR (ERIC-PCR)	The detection rates of target genes varied between ETP and environmental water isolates, with *blaCTX-M1* notably absent in ETP samples and present in only 6.56% of environmental isolates. While *blaTEM* prevalence was comparable across both groups, it remained the most frequent gene in environmental water at 54.10%, whereas *blaNDM-1* was most prevalent in ETP water at 79.31%. Furthermore, *blaSHV-1* and *blaVIM-1* were identified in 48.28% and 27.59% of ETP isolates, respectively, compared to 14.75% and 44.26% within the environmental water samples.	[267]

118.	*N.R.*	WWTP treated water samples<br>Potable water samples	Spain: Murcia	*blaCTX–M–1, blaTEM, sul1, tetA*	Culture-based method/qPCR	The prevalence and absolute abundance of the four target ARGs, *blaCTX–M–1, blaTEM, sul1,* and *tetA* varied significantly across the three irrigation water sources. Potable water exhibited the lowest ARG levels, with *blaCTX–M–1* and *tetA* falling below detection limits, while *sul1* and *blaTEM* were detected at low concentrations, log10 0.99 ± 0.35 and 1.18 ± 0.28 gc/100 mL, respectively. In contrast, both tertiary- and secondary-treated water showed 100% prevalence for all four genes, with secondary-treated water consistently displaying the highest absolute abundances, notably *sul1* (log10 5.80 ± 0.15 gc/100 mL) and *tetA* (log10 5.12 ± 1.40 gc/100 mL), while tertiary-treated water maintained significantly lower concentrations of these two genes compared to the secondary-treated samples.	[268]
119.	30 January – 21 March 2023<br>26 June – 25 July 2023	WWTP samples from the lower basin of the Llobregat River<br>WWTP samples from the Llobregat Delta corridor	Spain: Barcelona	*blaCTX-M-1 group, blaCTX-M-9 group, blaVEB, blaPER, blaTEM, blaSHV, blaGES, blaMOX, blaFOX, blaCIT, blaDHA, blaEBC, blaCPHA, blaKPC, blaVIM, qnrA, qnrB, qnrC, qnrS, aac(6')-lb-cr, aadA5, aph(6)-Id, aph(3")-lb, sul1, sul2, catI, cmlA, tetA, tetB, mcr-3, mcr-3.17, mcr-3.6*	Culture-based method/Standard PCR/ Illumina NextSeq	Among *Aeromonas spp*. isolates, diverse resistance genes were identified. ESBL producers harboured *blaCTX-M-1* group, *blaCTX-M-9* group, *blaVEB, blaPER, blaTEM, blaSHV*, and *blaGES*.blaAmpC-phenotype isolates carried *blaMOX, blaFOX,* and *blaCIT*, and carbapenem-resistant isolates contained *blaCPHA*, *blaKPC*, and *blaVIM*. Isolates non-susceptible to ciprofloxacin (CIP) carried *qnrS*; aminoglycoside-resistant isolates harbored *aac(6')-lb-cr, aadA5, aph(6)-Id*, and *aph(3")-lb*; sulfonamide-non-susceptible isolates possessed *sul1* and *sul2.* Chloramphenicol (CHL) non-susceptible isolates contained *catI* and *cmlA*, and tetracycline (TET) non-susceptible isolates carried *tetA* and *tetB*. While phenotypic colistin (COL) resistance testing was negative for *mcr-3*, sequencing revealed the presence of *mcr-3, mcr-3.17*, and *mcr-3.6.*	[269]
120.	Winter and summer 2018	Hospital sewer samples	Denmark, Spain, UK	*blaTEM, blaVIM, blaBEL, blaOXA, laCfxA, blaFOX,*	HT-qPCR/Illumina NextSeq	*blaTEM, blaVIM, blaBEL*, and *blaOXA* were ubiquitously detected on plasmids across all sewer compartments in all three countries, while *blaCfxA, blaFOX, blaGES, blaIMP, blaKPC,*	[270]

		Residential sewer samples<br>Influents of wwtps samples		*blaGES, blaIMP, blaKPC, blaCphA*		*blaOXA*, and *blaCphA* were exclusively abundant in Spanish hospital and receiving sewer samples. Notably, most of these clinically relevant ARGs were carried exclusively on non-transmissible plasmids, except for *blaCphA*, which was located on transmissible plasmids, suggesting a preferential association between specific ARGs and plasmid mobility types with implications for horizontal gene transfer risk.	
121.	August 2022 – June 2023	Soil samples	South Africa: Limpopo province	*blaSHV. blaCTX-M, blaTEM*	Culture-based method/Multiplex PCR	Among the three β-lactam resistance genes assessed in confirmed *E. coli* isolates, *blaTEM* was the predominant gene overall (33%, 70/211), while in soil samples specifically, *blaTEM* was detected in 26% (24/94) of isolates, *blaSHV* in 2% (2/94), and *blaCTX-M* was absent. Co-carriage of *blaSHV* and *blaTEM* was observed in 1% (1/94) of soil isolates.	[271]
122.	February and June 2024	21 river water samples<br>6 WWTP samples	Marocco	*blaCTX-M-15, blaOXA-1, blcCMY-42, blaDHA-1, blaCMY-2, blaLAP-2, blaADC-75, blaSHV-12, blaCTX-M-55, blaCTX-M-276, blaNDM-5, blaOXA-48, blaOXA-181, blaOXA-484, blaOXA-244*	Culture-based method/Illumina NextSeq 2000	Whole-genome sequencing revealed a diverse resistome dominated by β-lactam resistance genes, with *blaCTX-M-15* being the most prevalent (59.1%, n=13), followed by *blaOXA-1* (50.0%, n=11). Additional ESBL and *blaAmpC* genes were observed including *blaCMY-42* (13.6%, n=3), *blaDHA-1* (9.1%, n=2*), blaCMY-2, blaLAP-2, blaADC-75, blaSHV-12, blaCTX-M-55*, and *blaCTX-M-276* (4.5% each). Carbapenemase genes were also identified, including *blaNDM-5* and *blaOXA-484* (9.1% each, n=2), and single detections of *blaOXA-48, blaOXA-181*, and *blaOXA-244* (4.5% each).	[272]
123.	October – November 2020<br>March and April 2021	Seawater samples<br>Freshwater samples	France: Marseille	*blaTEM, blaSHV, blaCTX-a, blaCTX-b, blaOXA-23, blaOXA-58, blaOXA-24, blaOXA-48, blaNDM, blaKPC, blaVIM, mcr-1-5, mcr-8*	Culture-based method/RT-qPCR	ARG analysis across drinking water and wastewater samples identified a broad spectrum of resistance genes. In drinking water, *blaTEM-98* was detected in one *E. coli* isolate, *blaTEM-214* in one *E. cloacae* isolate, and *blaCTX-M-15* and *blaCTX-M-17* in an *E. coli* isolate from the Canal de Marseille source water. In wastewater, 43 of 114 Gram-negative isolates harboured ARGs,	[273]

		Drinking water samples<br>Wastewater samples				ESBL genes detected included *blaCTX-M-1, blaCTX-M-9, blaTEM*, and *blaSHV*, distributed across *E. coli*, *K. pneumoniae*, *C. youngae*, and *P. Mirabilis*. Carbapenemase genes identified were *blaKPC-2. blaOXA-48*, and *blaNDM-5*. Additionally, one *E. coli* isolate (CMP4c) co-harboured *blaOXA-10, blaCTX-M-9*, and *mcr-9*, representing the first detection of *mcr-9* in wastewater-derived *E. coli* in France.	
124.	March 2015	Water samples from the Soummam River	Algeria: Bejaia	*blaOXA-48, blaOXA-244, blaTEM-1, blaSHV blaCTX-M-15*	RT-PCR/Sequencing on ABI 3730	PCR screening and sequencing identified *blaOXA-48* as the predominant carbapenemase gene, detected in 17 isolates across multiple species (*E. coli* n=9, *K. pneumoniae* n=3, *R. ornithinolytica* n=3, *C. freundii* n=1, *C. braakii* n=1), while *blaOXA-244* was identified in three *E. coli* isolates. Co-production of *blaTEM-1* was observed in nine strains, and three strains co-harboured both *blaTEM-1* and *blaCTX-M-15.*	[274]
125.	29 April – 25 July 2025	Farm samples - water given to animals, farm soil, pasture, and animal bedding	Portugal	*laCTX-M-9-like, blaCTX-M-15-like, blaTEM, ermB, tetA, tetC, tetM, tetW*	RT-qPCR	At the gene level, *blaTEM* and *sul1* were more frequently detected in sheep farm samples, whereas *blaCTX-M-9*-like, *blaCTX-M-15*-like, and *sul2* were more prevalent in goat farm samples. Tetracycline resistance genes *tet(A), tet(C), tet(M)*, and *tet(W)* were generally more abundant in goat environments, though their distribution across individual gene variants was comparable between the two farm types. None of these differences reached statistical significance.	[275]
126.	September 2012 – September 2013	Water samples from different sources	Iran	*blaTEM, blaCTX, blaSHV, blaOXA, blaVEB*	Culture-based method/Standard PCR	Among 100 *E. coli* isolates, β-lactam resistance genes were detected at the following frequencies: *blaTEM* (37%), *blaCTX* (27%), *blaSHV* (27%), and *blaOXA* (25%), while *blaVEB* was not detected in any isolate. Multi-gene co-carriage was observed in 37% of isolates, with 11% harbouring two resistance genes, 12% harbouring three, and 14% harbouring four simultaneously.	[276]

127.	January, April, July, and October 2015	River water from upstream and downstream of the wastewater discharge point<br>Untreated and treated water samples	Poland	*blaCTX-M blaSHV,*<br>*blaTEM, blaOXA, (tet(A), tet(B), tet(C),*<br>*tet(D), tet(E), tet(K, tet(L), tet(M), tet(O), tetA(P), tet(S), tet(Q), tet(X), qnrS*	Culture-based method/ERIC-PCR	Of 292 amoxicillin-resistant isolates analysed, 256 harboured β-lactam resistance genes. *blaTEM* was the most prevalent across all sample matrices, while *blaOXA* was relatively more abundant in upstream river water (URW) samples. *blaCTX-M, blaTEM, blaSHV*, and *blaOXA* co-occurred exclusively in urban wastewater (UWW) isolates, whereas URW and downstream river water (DRW) isolates carried only *blaTEM* and *blaOXA*. Tetracycline resistance was predominantly mediated by *tet(A)* (19%), *tet(B)* (14%), and *tet(L)* (13%), with *tet(D)* absent in all isolates. Fluoroquinolone resistance was primarily associated with *qnrS*, detected in 60% of resistant isolates overall, with the highest prevalence in UWW (71%) and treated wastewater (TWW) (64%) samples.	[277]
128.	June 2015 – March 2016	Hospital wastewater samples<br>Municipal wastewater samples<br>WWTP samples	Switzerland: Basel	*blaVIM, blaKPC, blaNDM-1, blaOXA-48, blaTEM, blaSHV, blaCTX-M*	Culture-based method/Standard PCR	Among 49 isolates, *blaOXA-48* was the predominant carbapenemase gene (71.4%, n=35), followed by *blaNDM* variants (*blaNDM-1* n=2, *blaNDM-5* n=3, *blaNDM-9* n=1; 12.2% total), *blaKPC* (10.2%, exclusively in *K. pneumoniae*), *blaOXA*-181 (6%), and *blaVIM* (4%). Co-carriage of multiple carbapenemase genes was observed in four isolates (8%): two carried *blaOXA-48* and *blaVIM-1* (*E. coli* ST393 and *C. freundii*), and two *Citrobacter spp.* harboured *blaOXA-48* and *blaNDM-1* simultaneously. Additionally, 51% (n=25) of the isolates co-harboured *blaCTX-M* genes, predominantly *blaCTX-M-15* (56%), followed by *blaCTX-M-3* (24%), *blaCTX-M-24* (12%), and *blaCTX-M-14* (8%). *blaSHV-12* was detected in two isolates (4%), with *blaCTX-M-3* and *blaSHV-12* both restricted exclusively to *Citrobacter spp.*	[278]
129.	27 April 2011<br>26 May 2011	Sewage samples from two hospitals in the South	Brazil: Rio de Janeiro	*blaKPC, blaCfxA, blaGES, blaVEB, blaACC, blaFOX,*	Roche 454 pyrosequencing platform	Within Class A, *blaCfxA* was the most prevalent type (65% in SZ, 29% in NZ), with additional detection of *blaKPC* (11%) exclusively in NZ — with *blaKPC* genes taxonomically attributed to *K. pneumoniae*, *P. aeruginosa*, *S. marcescens*, *C.*	[279]

		Zone (SZ) and the North Zone (NZ)		*blaOXA-10, blaOXA-2, blaLCR-1*		*freundii*, and *E. coli*; *blaGES* and *blaVEB* types were detected in both samples (*blaGES*: 0.8% and 4.7%; *blaVEB*: 0.8% and 7.6% in SZ and NZ, respectively). Class C was dominated by *blaACC*-type in SZ (47%) and *blaACC* and *blaFOX* types in NZ (21% and 19%, respectively). Class D comprised *blaOXA-2* (22% SZ, 24% NZ), *blaOXA-10* (30% SZ, 32% NZ), and *blaLCR-1* (27% SZ, 20% NZ) in both samples.	
130.	Autumn 2014 – summer 2015	WWTP influent samples	Barcelona	*blaTEM, blaCTX-M-1, sul1*	Culture-based method/qPCR	Across all treatment processes, the bacterial fraction was generally more susceptible to ARG reduction than the bacteriophage fraction. Natural inactivation (particularly in summer) and UV treatment were the most effective at reducing all three genes (*sul1, blaTEM*, and *blaCTX-M-1*) in the bacterial fraction, with *blaTEM* and *blaCTX-M-1* falling below detection limits under both seasonal natural inactivation conditions. Chlorination also achieved highly significant reductions across all three genes in the bacterial fraction. Temperature treatments showed moderate effects, with reductions becoming more pronounced at 37°C, particularly for *blaCTX-M-1* and *blaTEM* in the bacteriophage fraction, while thermal treatments at 60°C and 80°C had minimal impact. pH conditions had a limited effect on the bacterial fraction but caused significant reductions in the bacteriophage fraction at pH 3 for all three genes.	[280]
131.	January 2009 – December 2012	Environmental samples<br>WWTP water samples<br>River water samples	Spain: Navarra	*blaTEM, blaSHV, blaOXA, blaCTX-M*	Multiplex PCR	Among environmental isolates, *blaCTX-M-1* was the predominant gene in river samples (37.0%), while *blaSHV-12* was the most prevalent in farm bed samples (51.0%).	[281]

		Far beds					
132.	November 2013 – April 2024	Raw influent, influent and effluent from 4 WWTPs	China: Harbin	*tetA, tetO, tetW,*<br><br>*sulI, sulII, blaTEM, blaSHV, blaCTX-M*	RT-qPCR	The six ARGs were detected in sewage and excess sludge across four WWTPs in Harbin, China. No significant seasonal differences in ARG content were observed between cold and warm sampling periods in the influent, suggesting that regional antibiotic use or other factors beyond temperature influence ARG fate in WWTPs. Among the ARGs investigated, *tet* genes were most readily reduced during wastewater treatment, while sul and *bla* genes proved more resistant to removal. Assessment of ARG removal efficiency across individual treatment units in Plant D revealed that biological treatment achieved the highest removal efficiency, followed by UV disinfection.<br><br>In the influent, the absolute abundances of *tetA, tetO, tetW, sulI, sulII, blaCTX-M, intI1*, and 16S rDNA ranged from $10^{4.18}$–$10^{5.05}$, $10^{4.35}$–$10^{4.91}$, $10^{6.12}$–$10^{6.70}$, $10^{5.49}$–$10^{5.84}$, $10^{6.88}$–$10^{7.53}$, $10^{3.72}$–$10^{4.69}$, $10^{6.49}$–$10^{7.36}$, and $10^{8.57}$–$10^{9.18}$ copies/mL, respectively.	[282]
133.	1923 – 2010	Soil samples from archive of the Askov-LTE	Denmark: South Jutland	*blaTEM, blaSHV, blaOXA, blaCTX-M*	qPCR	In pre-1940 soils, baseline abundances (log_{10} gene copies g^{-1} dry weight) were *blaTEM* (5.09 ± 0.11), *blaSHV* (4.27 ± 0.22), *blaOXA* (4.33 ± 0.44), *blaCTX-M* (4.61 ± 0.17), and intI1 (3.97 ± 0.24). No major differences in relative ARG abundances were observed before 1960. However, *blaTEM, blaSHV*, and *blaOXA* increased in manure-treated (M) soils from 1976, peaking in the mid-1980s before declining, while no equivalent increases were observed in inorganic fertiliser (IF) soils. *blaCTX-M* remained stable until the 1980s, then increased approximately 15-fold in M soils by 1988 before progressively declining through 2010. intI1 followed a slightly different trajectory, showing a shallow increase alongside *blaTEM, blaSHV*, and *blaOXA* in the 1970s–1980s, followed by a marked increase from the early 1990s through 2010, with all observed increases	[283]

						specifically associated with long-term manure application.	
134.	December 2014 – March 2015<br>December 2015 – March 2016	6 water samples from the Białka River<br>5 water samples from the Zakopianka River	Poland: Podhale	*blaCTX-M1, blaCTX-M3, blaTEM*	Culture-based method/Standard PCR	*blaTEM* was the most prevalent ESBL-encoding gene, detected in 89 isolates (45.4%), 42.9% from Białka and 47.9% from Zakopianka. *blaCTX-M-1* and *blaCTX-M-3* was also detected in a single ESBL-positive isolate recovered from site Z2, downstream of hospital sewage discharge in Zakopane.	[284]
135.	January 2015	12 soil samples from urban parks<br>2 soil samples from national parks	Austria: Victoria	*blaSHV, blaIMP-2, blaIMP-5, blaACT-1, blaMIR, blaACT-5/7, blaOXA-51, blaOXA-60, blaOXA-50.*	qPCR	β-lactam resistance genes of Classes A, B, C, and D collectively accounted for more than 50% of all detected ARGs in urban parks both with and without reclaimed water irrigation (RWI). The most prevalent genes within each class were: *blaSHV, blaIMP-2* group and *blaIMP-5* group, *blaACT-1* group, *blaMIR*, and *blaACT-5/7* group, and *blaOXA-51* group, *blaOXA-60*, and *blaOXA-50* group. *MLS_B* resistance genes were also frequently detected (19.07% and 16.34% in parks with and without RWI, respectively), alongside quinolone and fluoroquinolone resistance genes.	[285]
136.	*N.R.*	WWTP influent and effluent samples	Tunisia: Monastir Governorate	*laCTX-M,*<br>*blaTEM, qnrA, qnrS, sul I, and ermB*	qPCR	While *ermB* was significantly reduced following treatment at most facilities, *sul1* showed no significant relative reduction across any of the WWTPs, though absolute copy numbers declined significantly at Moknine. *blaCTX-M* and *qnrS* were altogether absent at certain sites (Sahline, Beni Hassen, and Moknine), suggesting site-specific ARG profiles. Notably*, blaCTX-M, blaTEM*, and *qnrS* were elevated in effluent from the Frina WWTP, which receives untreated hospital waste in addition to domestic and industrial sewage, highlighting hospital effluent as a key driver of ARG enrichment. Similarly, *blaTEM, qnrA*, and *sul1* were not reduced in	[286]

						effluent from Beni Hassen despite the application of UV disinfection following activated sludge treatment.	
137.	*N.R.*	Samples from the Tordera River streams	Spain: Catalonia	*blaCTX-M, qnrS, sulI, ermB*	qPCR	*blaCTX-M, qnrS, sul1*, and *ermB* were all significantly elevated at downstream and first downstream biofilm sites compared to upstream locations across multiple streams, with the most pronounced increases observed in the Santa Maria de Palautordera stream for *qnrS* and *ermB*. *blaCTX-M* was absent at upstream sites but consistently detected downstream, while sul1 and *ermB* increased in the Breda plant stream. A significant positive correlation between *ermB* and *qnrS* abundances suggests co-selection or shared dissemination pathways for these genes in stream biofilms	[287]
138.	*N.R.*	Wastewater and sludge samples from WWTP	China: Tianjin	*blaKPC-2, blaGES-1, blaIMP-1, blaVIM-2, blaOXA-48*	Culture-based method/Standard PCR/RT-qPCR	*blaKPC-2, blaGES-1*, and *blaIMP-1* were detected at all WWTP processing stages, while *blaVIM-2* and *blaOXA-48* were not detected in any sample. In the final effluent, *blaKPC-2, blaGES-1*, and *blaIMP-1* persisted at concentrations of $(1.54 \pm 0.61) \times 10^3$, $(2.14 \pm 0.41) \times 10^5$, and $(1.44 \pm 0.43) \times 10^5$ copies/mL, representing 0.7%, 22.8%, and 51.8% of influent levels, respectively, indicating incomplete removal across all treatment stages. Dewatered sludge contained substantially higher concentrations of all three genes, *blaKPC-2* $(6.51 \pm 0.14) \times 10^9$, *blaGES-1* $(6.18 \pm 0.63) \times 10^{10}$, and *blaIMP-1* $(3.49 \pm 0.81) \times 10^9$ copies/g.	[288]
139.	August 2014 and April 2015 June, 2016–April, 2017	Surface water<br>20 samples	U.S.A.: North Creek	*blaCTX-M, blaSHV, blaTEM, blaCMY-2*	Culture-based method/ qPCR	Multidrug resistant 39.58% *blaCMY-2* 18.36% *blaCTX-M*, *str*B, *sul1*, *tet*(A), *tet* (M) *blaCTX-M* were found in three water samples	[289]

140.	N.R.	Irrigation water and agricultural soil<br><br>19 samples	South Africa	*tet*A, *tet*B, *tet*C, *tet*D, *tet* E, *tet*G, *tet*K, *tet*L, *tet*M, *sul*I, *sul*II, *cml*A1, *cat*I, *cat*II, *str*A, *aad*A, aac(3)-IIa(aacC2), aph(3)-Ia(aphA1), aph(3)-IIa(aphA2), *blaTEM* (TEM-1 and TEM-2), *blaSHV* (SHV-1), *blaOXA*-1-like (OXA-1, OXA-4, OXA-30), *blaOXA*-48-like (OXA-48), *blaCTX*-M-1 (CTX-M-1, CTX-M-3, CTX-M-15), *blaCTX*-M-2 (CTX-M-2), *blaCTX*-M-9 (CTX-M-9, CTX-M-14), *blaCTX*-M group 8/25 (CTX-M-8, CTX-M-25, CTX-M-26, CTX-M-39 to CTX-M-41), *blaVEB* (VEB-1 to VEB-6), *blaPER* (PER-1, PER-3), *blaGES* (GES-1 to 8, GES-11)., plasmid-mediatedblaAmpC including *blaACC* (ACC -1, ACC-2), *blaFOX* (FOX-1 to FOX-5), *blaMOX* (MOX-1, MOX-2, CMY-1, CMY-8 to CMY-11, CMY-19), *blaDHA* (DHA-1, DHA-2), *blaCIT* (LAT-1 to LAT-3, BIL-1, CMY-2 to MY-7, CMY-12 to CMY-18 and CMY-21 to CMY-23), *blaEBC* (ACT-1, MIR-1) and	Culture-based method/ PCR assays	60% of the isolates confirmed as *E. coli* were collected from irrigation water samples, while 40% were collected from agricultural soil samples.<br><br>The most common resistance genes detected in the samples were *gulI, catII, tetA*, and *blaAmpC*. The *blaTEM* gene was also the most frequently identified gene. *blaPER*, and *blaGES* were also detected with a frequency of 4.2%.	[290]

				carbapenemases including *blaIMP* (IMP variants except for IMP-9, IMP-16, IMP-18, IMP-22 and IMP-25), *blaVIM* (VIM-1, VIM-2), *blaKPC* (KPC-1 to KPC-5)			
141.	N.R.	Water samples influent effluent, surface and ground water	India	*blaOXA-58, blaOXA-22, qnrA, qnrB, aac(6)-Ib-cr, aac (3)-XI, mec A, qepA, aadB, blaVIM, blaOXA-48* and *blaNDM*	Culture-based method/ PCR	The *qnrB* gene was found in 7 isolates (*Enterobacter aerogenes*), the *blaVIM* gene in 6 isolates (*Pseudomonas aeruginosa*) while the *blaNDM* resistance genes were present in three different isolates (*Klebsiella pneumoniae*); the *aac-6lb-cr* (*Aeromonas punctate*), *qepA* (*Citrobacter freundii*), *aadB* (*Stenotrophomonas maltophilia*), and *blaOXA*-58 and *blaOXA*- 22 (*Enterobacter aerogenes* and *Ralstonia picketti*). *aac (3)-XI* was not present in Gram-negative isolates.<br>Only three isolates showed ARGs *Corynebacterium sp.* (*aac(3)-XI*), *Staphylococcus aureus* (*mecA*) and *Staphylococcus aureus* (*qnrB*)	[291]
142.	N.R.	Hospital wastewater	Singapore	*blaNDM*, *blaKPC*, *blaSHV*, *blaCTX*-M, *aac(6')-Ib*, *sul1*, *sul2*, *dfrA*, *qnrA*, *qnrB*, and the *intI1*	Culture-based method/ qPCR	80.7% of isolates had *intl1*. Isolates were found to carry the *blaKPC*, *sul1, sul2, dfrA, aac(6')-Ib* and *qnrA* genes, as well as *intl1*. Concentrations of the ARGs ranged from 10^4 to 10^9 gene copies/ml. The cfr gene had an average concentration of 11.0 gene copies/ml, and the intI1 gene 2.31 × 106 gene copies/ml. *blaNDM, blaKPC, blaCTX-M* and *blaSHV* were detected at average concentrations of 2.29×10^6, 4.08×10^7, 1.25×10^6 and 6.19×10^5 gene copies/ml.	[292]
143.	N.R.	Hospital, wasteweter treatment plants, rivers, farms	South Africa: Eastern Cape Province	*blaNDM-1, blaKPC, blaOXA-48-like*	Culture-based method/ PCR	*Klebsiella spp.* isolates showed the highest incidence in soil samples (23%) from different effluent-insulated farms. The isolates from the hospital showed the *blaNDM-1* and *blaKPC* genes. These genes were also detected in WWTP samples.	[293]

						43% of *Klebsiella spp* isolates had carbapenem-resistant genes. Among these, *blaNDM*-110 (73), *blaKPC* (17) and *blaOXA*-48-like (10) were the most encountered.	
144.	2021 and 2023	Samples from the surrounding environment (soil and sewage)	China	*tet(X), blaEBR, and estT*	PCR	The results showed the presence of the *tet(X)* gene in sewage sample (30.6%) and soil samples (2.1%). *blaEBR* was found in 37 *Empedobacteri sp.* strains.	[294]
145.	March 2016	Hospital effluents	Brazil: Roraima	*blaSHV*, *blaCTX-M*, and *blaTEM*	Culture-based method/ PCR	The highest resistance rate was exhibited by *Klebsiella* sp. (42%), followed by *Pseudomonas sp.* (30%), *E. coli* (17%), and *Citrobacter freundii* (11%). *blaTEM* gene (37.5%) was found to be the most prevalent gene, followed by *blaSHV* gene (12.5%). The following gene associations were identified: blaTEM+*blaSHV* genes (12.5%). *blaCTX + blaTEM* (12.5%) and *blaSHV+ blaCTX-M +blaTEM* (25%).	[295]
146.	July 2021 to April 2022	Hospital effluents	Burkina Faso: Ouagadougou	*blaIMP*, *blaVIM*, *blaKPC*, *blaNDM*, and *blaOXA*-48	Culture-based method/ PCR	The following genes were detected in the studied isolates: *blaNDM* (71.43%), *blaVIM* (42.86%), *blaIMP* (28.57%), *blaKPC* (14.29%), *blaOXA*-48 (14.29%) in *E. coli*; *blaKPC* (68.42%), *blaNDM* (68.42%), *blaIMP* (10.53%), *blaVIM* (10.53%), and *blaOXA*-48 (5.26%) in *K. pneumoniae*.	[296]
147.	February 2018 and April 2019	Shoreline sediment samples	Canada: Bowring Park, Mundy Pond and Quidi Vidi Lake	*aac2'-Iia, aac3-IId, aadA, aadA2, aadA5, aadA8b, blaAmpC1, aph3'-Ia, aph3''-Ib, aph6-Id, cmlA1, blaCMY-2, blaCTX-M-15, blaCTX-M-27, blaCTX-M-55, dfrA1,*	Culture-based method/ PCR Illumina HiSeq	The highest frequency of multidrug-resistant strains was observed in samples collected from Pond Mundy (68.9%), followed by Bowring Park (61.8%) and Quidi Vidi (41.6%). Whole-genome sequencing revealed the presence of *blaCTX-M-14*, *blaCTX-M-15, blaCTX-M-27, blaCTX-M-55, blaCMY-2*, *blaDHA-1*, and *blaTEM-105*, *blaTEM-128*, *blaTEM-206*, *blaTEM-207* and *blaAmpC* in *E. coli* strains.	[297]

				dfrA8, dfrA12, dfrA14, dfrA17, blaDHA-1, floR, mphA, mphB, mphB4, mphB19, sul1, sul2, sul3, blaTEM-105, blaTEM-206, blaTEM-207, tetA, tetB *gyrA, parC, pareE,*			
148.	12 July 2023	Surface water	Brazil: Carioca River and Rodrigo de Freitas Lagoon	*blaSPM*, *blaSHV*, *blaTEM*, *blaCTX*-M, *blaKPC*, *blaNDM*, *blaOXA*-48-like, *blaGES*, *blaIMP*,, *blaVIM* *blaOXA*-23, *blaOXA*-24, *blaOXA*-51, *blaOXA*-58, *blaOXA*-143, *blaOXA*-235 *mcr*-1 to *mcr*-9	Culture-based method/ PCR Multiplex PCR	The *blaKPC* gene was detected in 20% of the isolated samples, the *blaTEM* gene, in 5%, *blaVIM* gene in 5% and *blaSPM* gene in 5% and *intl1* in 10%.	[298]
149.	May and August 2019	Surface water	Switzerland	*bla*KPC, *bla*NDM, *bla*OXA-48-like, *bla*VIM	PCR, WGS	*In the analysed samples, the genes blaKPC-2, blaKPC-3, blaNDM-5, blaOXA-48, blaOXA-181,* and *blaVIM-1* were detected.	[299]
150.	February 2020	Hospitals wastewater and treated wastewater	Mexico	*blaKPC, blaOXA-48-like, blaNDM-1* and *blaIMP*	Culture-based method/ PCR	*81.25% of the isolates of Klebsiella spp. wore blaKPC, while only one K. pneumoniae isolate showed blaKPC and blaOXA-48-like.*	[300]
151.	2014 – 2017	Hospital wastewater effluents	Slovakia; Czech Republic	*blaCTX-M-15, blaCTX-M-8/25, tetA* and *tetE*	Culture-based method/ multiplex PCR	*blaCTX-M-8/25 was identified in 10 isolates, while blaTEM was identified in 9 isolates.*	[301]
152.	July – September, October – December 2018	River water 108 samples	Nigeria	*blaCTX*-M, *blaSHV,* and *blaTEM*	PCR	In *A. hydrophila* isolates the frequency of detection ranged from 0% (*blaSHV*) to 26.3% (*blaCTX*-M), while for *E. coli* O157:H7 strains it was from 4.6% (*blaCTX*-M) to 58.4% (*blaEM*).	[302]

153.	February, April, July, September, and December 2017	Wastewater<br>River water	U.S.A. – Mexico Border	*blaTEM*, *blaSHV* and *blaCTX-M*	multiplex PCR	*blaCTX-M* gene was identified in 46.7% of isolates and the *blaTEM* gene in 60% of isolates. *blaSHV* gene was not detected.	[303]
154.	April – June 2022	Water samples	China	intI1, *tetA, ermA, ermB, qnrB, sul1, sul2, blaSHV*, and *blaCTX-M*	Culture-based method/ qPCR	The highest amount of ARG was found in water samples in the vicinity of hospitals, followed by those in wet areas. Most were transported by bacteria from *Acinetobacter*, *Aeromonas*, and various genera of *Enterobacteriaceae*. The abundance of the intI1 gene ranged from 4.25×10^2 copies/ml in hospital samples to 1.10×10^4 copies/ml in wet areas. The *tetA* gene also found more frequently in hospital and community samples than in wet areas. The *ermA*, *qnrB* and *sul2* genes were identified in the ambient water in the hospital, while the *blaSHV* had a greater abundance in the community region. *blaCTX-M* was found to be most abundant in hospital water samples, followed by those from the community and wetlands.	[304]
155.	September– Novermbe 2014	Sediment and water<br>4 samples	Croatia: Jadro River	blaOXA-*23-like, blaOXA-24-like, blaOXA-51-like, blaOXA-58-like, blaIMP, blaVIM, blaGIM, SIM, SPM, bla*TEM, *blaSHV, blaCTX-M, blaPER, VEB, blaGES/IBC, sul1 and sul2, intI1, intI2, intI3*	Multiplex PCR	The bla genes have been identified in all isolates analysed, particularly the *blaCTX-M* and *blaTEM* genes. Among the 12 *Acinetobacter* isolates, all carried *blaCTX-M-15*, while 11 isolates carried *blaTEM-116*, with 10 harboring both genes. Additionally, *blaTEM-1* was detected in one *A. calcoaceticus* isolate. *blaOXA-133* was identified in a *A. Radioresistens* isolate.	[305]
156.	June to September 2012 – 2013	Sediments hospital wastewater<br>45 samples	India: Cauvery	*blaTEM*, *blaCTX-M*, *blaSHV*, *blaNDM* and *aadA*	qPCR	High concentrations of the *aadA* and *blaTEM* genes were found in all sampling sites, while *blaSHV* and *blaNDM* genes were found in sediments contaminated by hospitals and city wastewater.	[306]

157.	August to December 2020 – 2021	Hospital effluents	India: Kerala	*bla*OXA-48, *bla*SHV, *bla*TEM, *bla*CMY, *bla*CTX-M	Culture-based method/ PCR	69.8% Gram-negative bacteria isolated from samples showed resistance to several drugs. The highest prevalence was found for *blaCTX-M* (n = 38) followed by *blaTEM* (n = 21) *blaOXA* (n = 3), *blaCMY* (n=5), and *blaSHV* (n = 18).	[307]
158.	January and February 2016	Industrial effluents and sediment samples	Croatia	N.R.	Culture-based method Sequencing	Industrial effluent and sediment from the receiving stream contained genes such as *blaGES-1* and *blaVEB-9* in *Klebsiella pneumoniae* and *Pseudomonas aeruginosa*. They also contained *blaCMY-10* in *Acinetobacter baumannii*.	[308]
159.	August – November 2015 and May – June 2016	Water samples Hospital wastewaters<br><br>24 samples	Colombia: Colombia city, Bogotá River	*AAC(3)-Ia, AAC(3)-Iia, AAC(3)-VIIa, AAC(6)-30/AAC(6)-Ib, AAC(6)-Ib, AAC(6)-Ib-cr, AAC(6)-Ib' AAC(6)-Ib11, AAC(6)-Ib3, AAC(6)-Ii, AAC(6)-IIa, aad(6), aadA11, aadA12, aadA16, aadA2, aadA21, aadA23,aadA24, aadA25,aadA5, aadA8, acrA acrD , acrE, acrF, acrS, aminocoumarin1, aminocoumarin2, amrA, ANT(2)-Ia, ANT(6)-Ia, ANT(6)-Ib, ANT(9)-Ia, APH(2)-Iia, APH(2)-Iva, APH(3)-Ia, APH(3)-Ib, APH(3)-IIa , APH(3)-IIIa H, APH(3)-VI, APH(6)-Id , arr-2, arr-8, arnA, bacA, baeR, baeS , Bifidobacteria, BlaB, bleomycin, catB2, catB3,catB8, catQ, catS, CblA-1, CcrA, CFE-1, CfxA, CfxA2, CfxA5, CfxA6, CMY-12, CMY-17, CMY-73, cphA5, cpxA, cpxR – CRP,*	Culture-based method/PCR Illumina MiSeq	Hospitals had the most ARGs, followed by the lower section (RL) and upstream river waters (river source (RS) and middle section (RM)). The lower section of the river had an abundance of ARGs comparable to hospitals. Genes present in pathogens have also been found in upstream water samples, which are considered less polluted by human activity. *blaCTX-M*, *blaTEM*, *blaKPC*, *blaNDM-1*, *blaVIM*, *blaZ*, *blaCTX-M*, *blaKPC*, *blaNDM*, *blaSHV*, *blaTEM* and *blaVIM* were present in hospital wastewater.	[309]

				CTX-M-101, CTX-M-107, CTX-M-11, CTX-M-117, CTX-M-12, CTX-M-72, CTX-M-9, dfrA12, dfrA14, dfrA15, dfrA17, dfrA19, dfrA21, dfrA8, dfrF, dfrG, efmA, efpA, emrA, emrB, emrD, emrK , emrR, emrY, Enterobacter - EreA2 - Erm(33) - ErmB ErmF - ErmG-1 - ErmG-2 - ErmT - Escherichia1 - Escherichia2 - evgA - evgS - floR - FosA3 - FosA5 - FOX-8 - gadE - gadW - gadX - GES-1 - GES-10 - GES-21 - GES-8 - H-NS Hospital 3.236558255 0.043114447 IMP-18 - kdpE - Klebsiella - KPC-1 KPC-11, KPC-13, KPC-15, KPC-22, KPC-8, KPC-9, LEN-13, leuO, lnuB, lnuC, lnuF, lsaA, lsaC, lsaE, marA, mdtA, mdtB, mdtC, mdtD, mdtE, mdtF, mdtG, mdtH mdtL, mdtM, mdtN, mdtO, mdtP, mecR1, mefA, mefB, mefC, mel, mfd,MOX-6,MOX-8, mphA, mphD,mphG – Mrx, msbA, msrB, msrC, msrE, NDM-10, NDM-12, NDM-9, NPS, oqxA, OXA-119, OXA-129, OXA-14, OXA-15, OXA-164, OXA-17, OXA-170, OXA-183, OXA-2, OXA-210, OXA-233, OXA-			

				239, OXA-25, OXA-253, OXA-27, OXA-31,OXA-333, OXA-334, OXA-34, OXA-36, OXA-36-2, OXA-361, OXA-368, OXA-370, OXA-398, OXA-415, OXA-420, OXA-422, OXA-454, OXA-46 , OXA-47, OXA-49 , OXA-53, OXA-56, OXA-9, OXA-96, patA, PC1, PER-3, plasmid, PmrC, PmrE, qacH, QnrB21, QnrB22 QnrB3, QnrB30, QnrB4, QnrB40, QnrB50, QnrB66, QnrB71, QnrB72, QnrD1, QnrS2, QnrS8, QnrS9, QnrVC1, sat-1,sat-4, SHV-115, SHV-135,SHV-159, SHV-167,SHV-29, SHV-34, sul1, sul2, sul3, TEM-10, TEM-101, TEM-102,TEM-103, TEM-106, TEM-108-1,TEM-108-2, TEM-112, -113,TEM-123, TEM-125-1, TEM-125-2, TEM-127, TEM-135,TEM-138,TEM-145, TEM-146, TEM-148, TEM-149-1,TEM-149-2, TEM-150, TEM-156,TEM-159,TEM-164,TEM-166,TEM-172, TEM-174, TEM-178,TEM-182,TEM-185, TEM-189, TEM-192 ,TEM-196, TEM-208-1, TEM-208-2,TEM-22, TEM-54-1,TEM-54-2, TEM-55-1,TEM-55-2,			

				TEM-57-1, TEM-57-2,TEM-59-1, TEM-59-2, TEM-89, TEM-95,tet31, tet32,tet39, tet40, tet44, tetA(P),tetB, tetC, tetD, tetE, tetL, tetM , tetO,tetQ H, tetS, tetT, tetW, tetX , tolC, vanA , vanHA, vanSA, vanSB,vanXA, vatE, VEB-9, vgaC VIM-23, YojI			
160.	March and December 2012	Effluents of treated wastewaters 37 samples	Czech Republic: Brno	*blaTEM*, *blaCTX*-M, *strA*, *sul2*, *tetB, sul*, *sul2, tetA*; *intI1,* class 1 integron, *qnrS,* *blaCMY*-2, *aac(6')-Ib*, *blaCTX*-M-27	Culture-based method/PCR/ WGS	A total of 450 *E. coli* isolates were obtained from water samples. At least one ARG was identified in each sample, with some isolates exhibiting multi-resistance. Sequencing of the *bla* and *qnr* genes showed the presence of *blaTEM-1*, *blaCTX-M-1* , *blaCTX-M-15*, *blaCTX-M-27, blaCTX-M-174* , *blaCMY*-2 and *qnrS1*.	[310]
161.	June and November 2018, and in March 2019	Municipal wastewater and river water	Poland: Warmia and Mazury regions	*blaGES*, *blaOXA*-58, *blaTEM*, *qnr*B, *qnr*S .*blaVEB*-1, *blaIMP*-1, *blaGES*, *blaOXA*-58, *blaCTX*-M, and *blaTEM* and *qnr*S	Illumina NovaSeq 6000 system	In untreated wastewater and primary clarifier samples, *blaGES* showed the highest abundance (2.36 and 2.34 ppm, respectively), followed by *blaOXA-58* (1.82 and 1.93 ppm), *blaTEM* (0.72 and 0.76 ppm), *qnrB* (0.25 and 0.36 ppm), and *qnrS* (1.08 and 1.45 ppm). Downstream river water also contained *blaVEB-1*, *blaIMP-1*, *blaGES*, *blaOXA-58*, *blaCTX-M*, *blaTEM*, and *qnrS*, with *A. baumannii* and *E. coli* identified among the associated bacteria.	[311]
162.	November 2013 to April 2014	Zoo water samples 7 samples	Brazil: Cuiabá-MT	*sul1, sul2, blaAmpC,* *t*et(K), tet(M), ermC, ermA, ermB, *blaPSE21*, *blaZ*, mecA, fenA, msrA, *aac(6')-aph(2''),* *ant(4')-Ia, aph3'-IIIa*	PCR	All of the analysed samples tested positive for resistance to at least three antimicrobial classes. *blaAmpC* was present in 66.7% of the samples, while *blaPSE21* was present in 77.8% of the samples. Only one sample (11.1%) tested positive for *blaZ*.	[312]
163.	21 and 24 July 2014	Drinking water samples 25 samples	Canada	*blaAmpC, blavanA, tetA, mecA, blaSHV, blaTEM, blaCTX, blaOXA1, blaCMY2, blaKPC,*	qPCR/Illumina MiSeq Platform	The isolates were positive for several antibiotic resistance genes, including *blaAmpC*, tet(A), mecA, β-lactamase genes (*blaSHV*, *blaTEM*, *blaCTX-M*, *blaOXA-1*, and *blaCMY-2*), and	[313]

				blaIMP, blaVIM, blaNDM, blaGES, blaOXA-48		carbapenemase genes (*blaKPC*, *blaIMP*, *blaVIM*, *blaNDM*, *blaGES*, and *blaOXA-48*).	
164.	Nov 2022 Feb 2023 Mar 2023 Oct 2023	Surface water	Philippines: Laguna Lake tributaries	*blaTEM*, *blaCTX*-M, and *blaSHV*	Culture-based method/ mPCR	38.9% of *E. coli* isolates harbored at least one *bla* gene. The most abundant was *blaTEM* followed *blaCTX*-M and *blaSHV.*	[314]
165.	14 February, until 17 December 2018	Water samples	U.S.A.: Bowling Green, Kentucky; Urban Karst, Florida	*erm*B, *msr, msr*C, *erm*Q, *tet*M, *tet*L, *tet*O, *tet*S, *tet*A, *tet*B, *blaCMY*-2, *blaCTX*-M	Culture-based method/ end-point PCR	Antibiotic-resistant *Enterococcus spp* and *E. coli* have been identified in the studied areas. *E. coli* isolates from Bowling Green were found to carry the *blaCTX-M* (76%) and *blaCMY-2* (23%) genes. The *blaOXA*-1 (1%) and *blaDHA* (1%) genes were also detected. In Florida, the *blaOXA*-1 (11.8%) gene was detected, whereas *blaDHA* was not present. *blaCMY*-2 (58.8%) has the highest prevalence in Florida.	[315]
166.	December 2022 and March 2023	Sewage samples	Bangladesh: Dhaka City	*blaCTX*, *blaTEM*, *blaOXA1*, *blaSHV*, *blaNDM*-1, and *blaKPC*	PCR	Antibiotic-resistant strains of *Escherichia coli* (42.7%), *Acinetobacter baumannii* (25.9%), *Pseudomonas aeruginosa* (25.9%), and *Enterobacter cloacae* (5.6%) were identified. The most prevalent gene was *blaNDM*-1(100%), followed by *blaOXA*-1(41.2%), *blaSHV* (5.9%), *blaCTX*-M (5.9%), and *blaKPC* (5.9%). The *blaTEM* gene was not identified in any of the samples collected.	[316]
167.	N.R.	Raw wastewater	Brazil: São Leopoldo	*blaSPM* and *blaNDM*	Culture-based method/Standard PCR/ Multiplex PCR	*Pseudomonas aeruginosa* was identified in the analysed samples. *blaNDM* and *blaSPM* genes were present in the *Pseudomonas aeruginosa* isolates.	[317]
168.	June and October 2014 April and October 2014	Influent and effluent wastewater	Croatia: Zagreb	*blaOXA*-51-like, *blaOXA*-40-like, *blaOXA*-23-likeand *blaOXA*-58-like	Culture-based method/ end-point PCR,	*Acinetobacter baumannii* was detected in 21 wastewater samples (15 from the influent and six from the effluent). All 14 *A. baumannii* isolates carried the constitutive *blaOXA-51-like* gene. Of these, 10 also harbored *blaOXA-23-like*, three carried *blaOXA-40-like*, while one lacked any acquired *blaOXA* gene.	[318]

169.	N.R.	Hospital wastewaters	Burkina Faso	*bla*CTX-M, *bla*SHV, *bla*TEM, *bla*DHA, *bla*FOX, *bla*OXA-48, *bla*NDM, *bla*VIM, *bla*KPC and *bla*IMP	PCR	*E. coli and Klebsiella spp. have been identified in the hospital wastewater. The blaCTX-M gene was the most abundant (88.7% of E. coli and 85.7% of Klebsiella spp.), followed by blaTEM (66.0% and 71.4%), blaNDM (15.1% and 7.5%), and blaOXA-48 (9.5% and 9.5%). 1.9% of E. coli and 4.8% of Klebsiella spp. in the wastewater also carried ESBL, blaAmpC, and carbapenemase genes.*	[319]
170.	2013	Soil samples	Antarctica: Ardley Island	*blaOXA*−23−like *blaTEM*−1B, *blaSCO*−1, aac(3)-IIa, *blaPER*−2(pl); aph(3′)-VIa(pl); strA(pl);strB(pl);tet(B)(pl)	PCR/WGS	*Acinetobacter spp* has been identified in soil samples. *blaPER*−2, *blaSCO*−1, *tet(B)*, *aph(3')-Vla*, *strA* and *strB* were identified.	[320]
171.	Spring 2016	Sewage water	Lebanon: Al-Qaa	*blaTEM*, *blaSHV*, *blaCTX*-M, *blaOXA*, *blaampC*, *blaVIM-1*, *blaVIM-2*, *blaNDM*, *blaKPC*, *blaKPC*-2, *blaIMP*, *blaOXA*-48, aac(6′)-1b, aac(3′)-II, ant(2′′)-Ia, ant(4′)-Iia, aph(3′)-Ia, armA I, rmtB, gyrA and parC	Culture-based method/ PCR	*Pseudomonas spp.*, *Acinetobacter spp.*, *Pasteurella spp.*, *Aeromonas spp.*, *Proteus spp.*, *Klebsiella spp.*, *Enterobacter spp.*, *Alcaligenes spp.* and *E. coli* isolates were selected for ESBL screening.<br>53.1% of the isolates taken from samples in the Al-Qaa region had ARGs (*blaCTX-M-14*, *blaOXA-1*, *blaSHV-12*, *blaCMY-2*, *aac(6)-Ib*, *acc(6)*, *acc(3)-II* and *Int-I1*), compared to 49.1% of the isolates taken from river effluents. The following genes were not identified: *armA*, *rmtB*, *ant(4')-Iia*, *aph(3')-Iia*, *blaVIM-1*, *blaVIM-2*, *blaNDM*, *blaKPC*, *blaKPC-2* and *blaIMP*. Additionally, the *blaCTX-M*, *blaTEM* and *blaSHV* genes were detected at higher percentages in the isolates from the two locations, with similar percentages observed for the *blaCTX-M* and *blaTEM* genes.	[321]
172.	December 2019, August 2020 and November 2020	Wastewater	Czech Republic	*blaTEM*, *blaNDM*-1, *blaKPC*, *blaOXA*-48, mecA, tetW, and vanA	Culture-based method/ qPCR	Multi-resistant strains have been identified in *Aeromonas sp.* The *blaTEM*, *tetW*, *blaNDM*-1 and *vanA* genes were detected. The largest abundance was observed in *blaTEM*, followed by *tetW*, *blaNDM*-1 and *vanA*.	[322]

173.	N.R.	River	India: Cuttack City, Kathajodi River	*blaKPC, blaNDM, blaIMP, blaOXA-40, blaOXA-58, blaOXA-23, blaOXA-48*	Culture-based method/ Standard PCR	The *blaNDM* gene was the most prevalent, being present in 55 (75.34%) of the isolates, followed by the *blaOXA-48*-like gene (31/73; 42.46%), and the *blaKPC* gene (8/73; 10.95%). *blaOXA-40, blaOXA-58*, *blaOXA-23*, and *blaIMP* were not detected. CRAB isolates were co-harboured by *blaTEM* (79.45%; n = 58), *blaCTX-M* (24.65%; n = 18), and *blaSHV* (21.91%; n = 16).	[323]
174.	N.R.	Wastewater from hospital settings, hospital washroom water, and rainwater	Bangladesh: Dhaka	*blaNDM, blaCTX-M1, blaTEM, blaSHV1, blaVIM1*	Culture-based method/ Standard PCR	Hospital wastewater samples contained *blaTEM* and *blaCTX*-M-1, with prevalence rates of 76.09% and 71.74%, respectively, followed by *blaVIM-1*, at 63.04%. *blaSHV-1* had a frequency of 41.30%, and *blaNDM* was present in 67.39% of the isolates.	[324]
175.	September 2022 (spring) and 2023, January (summer), June (autumn), and August (winter)	Hospital wastewater	Brazil: Curitiba	*blaKPC, blaNDM, blaOXA-48-like, blaVIM, blaIMP, blaSPM, blaGES and blaOXA-23-like, vanA*	Culture-based method/ Sequencing: Illumina Nextera XT	The most prevalent gene, *blaKPC*-2, was detected in 84.6% (44/52) of the isolates, followed by *blaGES*-5 (5.8%, 3/52), *blaNDM*-1 (3.8%, 2/52) and *blaKPC*-3 (1.92%, 1/52).	[325]
176.	February 2021 – February 2022	Untreated community wastewater from WWTP<br><br>89 samples	Finland	N.R.	Sequencing: Illumina NovaSeq 6000	The most frequently detected genes in *E. coli* were: *blaKPC-2* (6/26, 23.1%), *blaKPC-3* (5/26, 19.2%) and *blaNDM-5* (4/26, 17.4%). In *K. pneumoniae*, the most prevalent was *blaKPC-3* (11/25, 44.0%), followed by *blaOXA-48* (4/25, 16.0%). The presence of *blaCTX-M-15*, *blaCTX-M-14* and *blaSHV-27* was also observed.	[326]
177.	August – October 2024	Raw wastewater	Chile: Puerto Montt	N.R.	Sequencing:<br><br>Illumina NovaSeq 6000	*blaCTX-M-15*, *blaGES*-14, *blaKPC*-1, *blaOXA*-1, *blaOXA*-10, *blaOXA*-101, *blaOXA*-129, *blaOXA*-146, *blaOXA*-17,<br><br>*blaOXA*-2, *blaOXA*-205, *blaOXA*-209, *blaOXA*-211, *blaOXA*-280, *blaOXA*-333,	[327]

						blaOXA-360, *blaOXA*-464, *blaOXA*-58, *blaPER*-1 were detected	
178.	March – December 2024	Untreated hospital wastewater, treated hospital wastewater, and community water	Thailand: Chonburi	*blaNDM, blaKPC, blaOXA−23, blaOXA−24, blaOXA−51, blaOXA−58*	RT-PCR; Sequencing: Illumina MiSeq Benchtop	*blaNDM, blaTEM, blaSHV, blaOXA, blaCTX−M−1, blaCTX−M−9, blaCTX − M−8/25, blaDHA, blaGES, blaVEB* were found in *K. pneumoniae* isolates*; blaNDM, blaGES, blaVEB* were present in *P. aeruginosa; blaNDM, blaTEM, blaOXA – 23, blaOXA – 51, blaOXA – 69* were also found in *A. baumannii. blaKPC, blaNDM, blaTEM, blaOXA, blaOXA–48, blaCTX − M−1, blaCTX − M−9, blaCTX − M−8/25* were present in *E. coli; blaNDM, blaTEM, blaCTX − M−1, blaCTX − M−8/25, blaGES, blaVEB* were found in *E. cloacae* isolates.	[328]
179.	From 2016 to 2017, every May	River<br>15 samples	South Korea: Han River	*blaOXA*-harbouring strains, *blaGES*, *blaNMC*, *blaKPC*, *blaPER*, *blaSME*, *blaSIM*-1, *blaIND*, *blaIMP*, *blaNDM*, *blaVIM*, *blaOXA*-23 subfamily, *blaOXA*-51 subfamily	PCR; Sequencing: PacBio RS II (Pacific Biosciences, U.S.A.) platform	*blaOXA-334* and *blaOXA-652* were detected in *Acinetobacter johnsonii* from the Han River.	[329]
180.	N.R.	Hospital wastewater, Treated wastewater<br>158 samples of untreated wastewater	Gabon: Libreville, Franceville, Lambaréné, Tchibanga, Port-Gentil, Moanda	*blaCTX-M-1, blaTEM, blaSHV*	Standard PCR	The most prevalent gene from *E. coli* isolates was *blaCTX-M-1* (66.6%), followed by *blaSHV* (33.3%). *blaTEM* was not detected.	[330]
181.	December 2021 – August 2022	Raw sewage, treated sewage,	Brazil: Santa Catarina	*blaCTX-M, blaSHV, blaTEM, blaKPC*	Standard PCR	*blaCTX-M*, *blaSHV*, *blaTEM* and *blaKPC* were detected in *E. coli*, *Citrobacter farmeri*, *Citrobacter freundii*, *Klebsiella pneumoniae*,	[331]

		sludge, and bioaerosols<br><br>20 samples				*Burkholderia cepacia* complex, and *Aeromonas hydrophila* complex isolates.	
182.	March – May 2023	Influent and effluent wastewater, river<br><br>18 samples	Nepal: Kathmandu	*blaNDM*, *blaIMP*, *blaVIM*, *blaKPC*, *blaOXA*, *blaTEM*, *blaCTX-M*, and *blaSHV*	RT-qPCR	*blaNDM*, *blaKPC*, and *blaOXA* were found in Carbapenemase-producing bacteria. *blaIMP*, *blaVIM* were not found. The prevalence of *blaNDM* was highest in municipal influent (46.67%), while *blaOXA* was more prevalent in isolates from river water (50.00%). The most abundant beta-lactamases in influent wastewater were *blaCTX-M* (60.00%), *blaSHV* (54.55%) and *blaTEM* (52.94%). *blaNDM* (40.00 %) was frequently found in *E. coli* isolates. *blaKPC* (40.00 %) and *blaOXA* (25.00 %) were often found in *K. pneumoniae*. The *blaKPC* and *blaOXA* genes were found together in two *K. pneumoniae* strains, one *C. freundii* strains and one *Acinetobacter* sp.	[332]
183.	8 November 2022, 6 February 2023, 19 May 2023	Sediments from aquacultur e	China: Jiangsu Province	*blaTEM*	RT-qPCR	*blaTEM* gene was detected in sediment samples.	[333]
184.	N.R.	Influent and effluents at different stages of the WWTPC<br><br>7 samples	India: Aligarh	*N.R.*	Sequencing: Illumina HiSeq 4000	*blaNDM-1* was present at all stages except one WWTP. Various ARGs, including beta-lactamases (*blaNDM-43*) and *blaOXA* variants (*blaOXA-114c*, *blaOXA-140*, *blaOXA-21*, *blaOXA-246*, *blaOXA-282*, *blaOXA-283*, *blaOXA-284*, *blaOXA-347*, *blaOXA-373*, *blaOXA-496*, *blaOXA-58*, *blaOXA-644*, *blaOXA-646*, *blaOXA-900* and *blaOXA-915*), were absent in the influent but were detected at later stages of treatment.	[334]
185.	November 10, 2022	Surface water	Brazil: Carioca River and Rodrigo de Freitas	*blaSPM*, *blaSHV*, *blaTEM*, *blaCTX−M*, *blaKPC*, *blaNDM*, *blaOXA−48−like*, *blaGES*, *blaIMP*,	Standard PCR	The following genes were detected in bacterial isolates: *blaKPC* (41%; 19/46), *blaGES* (26%; 12/46), *blaNDM* (6%; 3/46), *blaCTX−M* (6%; 3/46) and *blaVIM* (2%; 1/46).	[335]

			Lagoon, Rio de Janeiro	*blaVIM, blaOXA−23, blaOXA−24, blaOXA−51, blaOXA−58, blaOXA−143, and blaOXA−235*			
186.	October 2022 – June 2023	Lake water	Philippines: San Pablo (Bunot, Calibato, Mohicap, Palakpakin, Pandin, Sampaloc, and Yambo Lakes)	*blaCTXM*, *blaTEM*, *blaSHV*, *blaAmpC*, *blaKPC*	Culture-based method/ Standard PCR	Of the 846 isolates, 487 harboured *blaAmpC*, 21 harboured *blaTEM*, 10 harboured *blaSHV* and two harboured *blaCTX-M*. *blaKPC* was not detected in any of the isolates. Of the isolates harbouring two β-lactamase genes, 257 possessed *blaTEM*/*blaAmpC*; 16 were detected with *blaSHV/blaAmpC* in both the wet and dry seasons; Three harboured *blaCTXM*/*blaAmpC* and two harboured *blaTEM*/*blaSHV* during the dry season. No isolates were identified with the *blaTEM/blaCTX-M* or *blaSHV/blaCTX-M* gene combinations. Eight isolates harboured *blaSHV*/*blaTEM*/*blaAmpC*. The various *E. coli* isolates exhibited a high prevalence of *blaAmpC* and a combination of *blaAmpC*/*blaTEM* among the various β-lactamase resistance patterns.	[336]
187.	26 February 2023	Raw municipal sewage effluent<br><br>16 samples	India: Cuttack	*blaKPC, blaNDM, blaOXA-48-like, blaIMP, blaVIM, blaSHV, blaTEM* and *blaCTX-M*	Standard PCR	The isolates harboured several genes, including *blaKPC* (18.18%), *blaIMP* (18.18%), *blaNDM* (9.09%), *blaVIM* (54.54%) and *blaOXA-48-like* (54.54%). The most frequent combination was *blaVIM-blaOXA-48-like* (45.45%), followed by *blaNDM* (78.5%) and *blaOXA-48-like* (71.4%) in clinical CRKP isolates, with *blaVIM* (14.3%) being the least abundant. *blaKPC* and *blaIMP* were not detected.	[337]
188.	January – December 2015	River water<br><br>27 samples	Ecuador: Monjas, Machángara, San Pedro, Pita, and	*blaSPM, blaSIM, blaGIM, blaGES, blaOXA-23, blaOXA-24, blaOXA-51, blaOXA-58, and blaOXA-143*	Standard PCR	Seven river isolates showed the presence of the *blaOXA*-51 gene, as well as possibly the *blaOXA-259* gene	[338]

			Guayllabamba				
189.	January – December 2020	Influent and effluent wastewater<br>11 influent samples,<br>25 effluent samples	Portugal	*aphA1/7, aadA1, strB, catB3, floR 1, cmlA 2, ereA-1, ermB-1, matA/mel, mphA-2, qnrA, qnrB, blaVEB, blaOXA10-1, blaGES, blaVIM, mecA, blaSHV-2, blaTEM-1, blaPER 1, blaIMP-2, blaCTX-M-8, blaNDM, blaKPC-2, mdtE, sul3 1, sul1 2, tetA 2, tetW, dfrA1-1, dfrA12, intI1 2, intI3 1, intI2 2, acrA 3, sul2 2, tetM-3, qnrS-1, mcr1, vanA, blaOXA48*	Quantification: High-throughput quantitative PCR (HT-qPCR)<br>Sequencing: Illumina MiSeq	*blaVEB, blaOXA10-1, blaGES, blaVIM, blaSHV-2, blaTEM-1, blaPER-1, blaIMP-2, blaCTX-M_8, blaNDM, blaKPC-2* were found to positive in influent wastewater.<br>*blaOXA10-1, blaGES, blaVIM, blaSHV-2, blaTEM-1, blaPER-1, blaCTX-M-8, blaKPC-2* were present in effluent wastewater.	[339]
190.	March 2020	River water<br>20 samples	Vietnam: Hue City	*blaTEM*, *blaSHV*, *blaCTX-M*, *qnrA*, *qnrB*, *qnrS*	Culture-based method/ PCR standard	58.3% of *E. coli* isolates carried *blaTEM* and *blaCTX-M-15*, 16.7% had *blaTEM* and *blaCTX-M-65*, and none carried *blaSHV*.	[340]
191.	November 2021 – January 2022; September 2022 – November 2022	Lake water<br>15 samples	Côte d'Ivoire: Yamoussoukro	*blaTEM*, *blaSHV*, *blaCTX-M*	Culture-based method/ Standard PCR	10 *E. coli* strains were found in lake samples, and 5 from lettuce samples. *E. coli* isolates were found to be positive for *blaCTX-M* gene (100%). The *blaTEM* gene was identified in 11 of the isolates (73.33%), alongside the *blaCTX-M* gene, whereas *blaSHV* gene was not found in any of the samples.	[341]
192.	June 2022 – July 2023	Wastewater from dairy farms	Indonesia: Pasuruan District, Malang District, Tulungagung District, Blitar	*blaSHV* and *blaTEM*	Culture-based method/ Standard PCR	*K. pneumoniae* isolates harboured *blaSHV* 63.1% (12/19) and *blaTEM* 31.57% (6/19). In Blitar, 33.33% (4/12) of isolates were positive for both *blaSHV* and *blaTEM*. In Malang, 8.33% (1/12) of isolates were positive for both genes, while in Batu City and Pasuruan, both of which had 25% (3/12) of isolates positive for both genes. In	[342]

			District, Batu City and Kediri District			Tulungagung, 8.33% (1/12) of isolates were positive for only *blaSHV*.	
193.	11 May 2022 – 02 June 2022; March 2022 – May 23 2022	Hospital wastewater<br>21 samples	France: Montpellier	*blaCTX-M*, *blaOXA-48*, *blaNDM*, *blaKPC* and *vanA*	Culture-based method/ qPCR and dPCR	The carbapenemase-encoding genes *blaOXA*-48, *blaKPC* and *blaNDM* were present in more than half of the isolated bacteria (76/145 = 52%)<br>The following carbapenem resistotypes were observed in Enterobacterales: *OXA-48*-producing *Citrobacter freundii* (20 isolates from HRR and 1 isolate from SRR), *blaKPC*-producing *C. freundii* (10 isolates from each building), and *KPC*-producing *C. braakii* (9 isolates from HRR and 2 isolates from SRR). Six strains carried both the *blaOXA*-48 and the *blaKPC* genes (four *C. freundii*, one *C. braakii* and one *Klebsiella oxytoca*); all of these were isolated from HRR wastewater. Overall, 49 out of 53 *Citrobacter spp.* strains isolated in this study encoded the *blaOXA*-48, *blaKPC* or *blaNDM* genes.	[343]
194.	September/ November 2018 – March 2019; October 2018, April and July 2019	River water	Italy: Monte Ossolano	*blaTEM* and *mefA/E*	Culture-based method/ qPCR	*blaTEM* was present in 58% of the isolates (69 colonies). *blaTEM* was found in isolates from all antibiotic treatments, most frequently in plates supplemented with ampicillin (15 isolate) and amoxicillin (13 isolates).	[344]
195.	1 June – 15 September 2021	Marine water and freshwater<br>186 samples	Northen Ireland: Waterfoot, Carnlough, Ballygally, Brown's Bay, Ballyholme, and Newcastle	*blaOXA-48*, *blaKPC*, and *blaNDM-1*	RT-qPCR	AMR gene *blaOXA-48* was identified in all six freshwater locations. However, *blaNDM-1* was not detected in any of the samples, whereas *blaKPC* was present in only one sample.	[345]

196.	January 2023 – July 2024	Wastewater, freshwater	India: New Delhi	*blaCTX-M, blaTEM, blaSHV, blaNDM, blaOXA-48, blaKPC, blaIMP, blaCMY, sul1*<br>*DfrA12, DfrA17, qnrA*<br>*qnrB, qnrS, intl1, intl2, tetA, tetB, aac(3)-II, fimH, iucC, mrkD, rmpA, ecpA*	Culture-based method/ Standard PCR	*blaTEM* was present in all isolates. Meanwhile, *blaSHV* was detected in 76% (25) of strains, and *blaCTX-M* in 55% (18). The genes *blaNDM* and *blaOXA−48* were found in 27% (9 out of 33) and 39% (13 out of 33) of the strains, respectively. *blaCMY* was present in 48% (16 out of 33) of the samples. 15% (5 out of 33) *K. pneumoniae* isolates contained all the β-lactamase genes (*blaCTX−M*, *blaTEM*, *blaSHV*, *blaNDM*, *blaOXA−48*, *blaCMY*).	[346]
197.	N.R.	Leachate and surface water<br>64 samples	Nigeria:<br>Ibadan	*blaSHV, blaTEM, blaCTX−M*	Culture-based method/ Standard PCR	*blaCTX-M* was found in all 35 *E. coli* isolates, 60% (21) carried the *blaTEM* gene, while *blaSHV* was not detected in any of the isolates.	[347]
198.	N.R.	Hospital influent and effluent wastewater	Philippines: Manila, Quezon City	*blaCTXM-1, bla CTXM-9, blaTEM-1,* and *blaSHV-1*	Culture-based method/ Standard PCR	Two and three of the four *E. coli* isolates from influent wastewater carried *blaCTX-M* and *blaTEM*, respectively. *blaSHV* was not detected in the influent. Isolates from the effluent tank contained the *blaCTX-M* gene. One isolate carried the *blaCTXM-1* gene, while the other carried the *blaCTXM-9* gene.	[348]
199.	September 2020	River	Indonesia: Citarum River	*blaCTX-M-15*	Culture-based method/ Standard PCR	No *blaCTX-M-15* was detected in *E. coli* isolates from Citarum River.	[349]
200.	September 2022 – August 2023, July 2024	Wastewater influent<br>172 samples	U.S.A: Milwaukee	*blaKPC, blaOXA-24/40, blaOXA-48, blaVIM, blaIMP, blaNDM*	ddPCR	*blaKPC*, *blaOXA*-24/40, and *blaOXA*-48 were present in high concentrations. *blaVIM* and *blaIMP* were present in 99% of samples in lower concentration (mean of ~1.5E+05 cn/L and 1.0E+04 cn/L, respectively). *blaNDM* was found in 49% of samples (~1.0E+03 cn/L).	[350]
201.	September 2012 –	River, sediments	Spain: Catalonia	*blaTEM, blaCTX-M, qnrA, qnrS, mecA, sul1*	Culture-based method/qPCR	All samples were positive for at least one ARG across both bacterial and bacteriophage fractions.	[351]

	December 2014	139 samples				The prevalence of *blaTEM* was high in both bacteria (97%) and bacteriophages (100%). The presence of *blaCTX-M* was found to be lower in both the bacterial (48%) and bacteriophage (41%). Generally, ARG concentrations in the bacterial fraction were equal to or higher than those in the bacteriophage fraction across all genes.	
202.	3, 10, 17 March 2017	WWTP influent	Canada: Arnprior, Ottawa, Toronto	*blaKPC, blaKPC-2, blaCTX-M, blaPER, blaOXY-6, blaOXY-6-2, blaOXA-10, blaOXA-62, blaOXA-153, ampS, blaACT, blaACT-4, blaACT-9, blaACT-28, blaCARB, blaCEPH, cphA, blaFOX, blaMOX, blaOXY, blaL1, blaPAM.*	Culture-based method/Standard PCR/Illumina MiSeq	PCR and WGS analyses identified a broad range of β-lactamase genes across isolates from both WWTPs. *blaKPC* was among the most common ARGs identified by PCR, found in 9 *Klebsiella* isolates. All sequenced *K. oxytoca* isolates co-harboured *blaKPC-2* and *blaOXY-6-2*. PCR initially identified *blaCTX-M* (n=8) and *blaPER* (n=1) as class A ESBLs. However, WGS revealed that four *blaCTX-M* PCR-positive isolates instead carried *blaOXY-6*, attributable to cross-reactivity of universal CTX-M primers with OXY-type. Additional β-lactamase genes detected exclusively by WGS included *ampS, blaACT, blaCARB, blaCEPH, cphA, blaFOX, blaMOX, blaOXY, blaL1*, and *blaPAM*. Notably, *blaOXA-153* was detected in two *P. apista* isolates, with *blaOXA*-62 only detectable at reduced identity thresholds (80%). Similarly, *blaL1* was identified in four S. maltophilia isolates, and *blaACT-4* and *blaACT-9* were identified in an *E. asburiae* isolate at lower thresholds.	[352]
203.	2017	Wastewater samples from different sites on the Lis River	Portugal	*blaTEM-1, blaOXA-9, blaKPC-3, blaSHV-1, aacA4, aadA1, strB, dfrA14*	Culture-based methods/BOX-PCR/Illumina HiSeq 2500	Both *K. pneumoniae* isolates (CR12, ST231, and CR20, ST147) harboured *blaKPC-3*. In CR12, additional resistance genes included β-lactam resistance genes, *blaTEM-1*, *blaOXA-9*, aminoglycoside resistance genes, *aacA4*, *aadA1, strB,* sulfonamide resistance, *sul2*, and trimethoprim resistance, *dfrA14*. All of these were absent in CR20. Both isolates harboured the fosfomycin resistance gene fosA and naturally occurring β-lactamase genes *blaSHV-1* in CR12 and *blaSHV-11* in CR20. Fluoroquinolone resistance-associated mutations were also identified: the S80I substitution in *parC* was present in both isolates, while the S83I	[353]

						substitution in *gyrA* was detected exclusively in CR20.	
204.	September 2016	Lake water	Romania: Buzău, Brăila	*blaOXA-48, blaNDM, blaTEM, blaSHV, blaCTX-M, blaOXA-23, blaOXA-24, blaOXA-51, blaOXA-58, blaOXA-143, blaOXA-235, blaKPC, blaVIM-2, qnrA, qnrB, qnrS, aac3Ia, tet(A), tet(B), tet(C), tet(D), tet(M), sul1, sul2, intI1, dfrA1-aadA1.*	Culture-based methods/Simplex and Multiplex PCR	Molecular screening identified a broad range of ARGs across isolates from fishery lakes. Among β-lactam resistance genes, *blaCTX-M-like* (33%), *blaNDM-like* (8.33%), and *blaIMP*-like (8.33%) were detected, encoding ESBLs and carbapenemases. Non-β-lactam resistance was mediated by *sul1* (75%) and *sul2* (4.16%) for sulfonamides, *tet(A)* (25%) and *tet(C)* (15%) for tetracyclines, *qnrS* (4.16%) for quinolones, and *aac3Ia* (4.16%) for aminoglycosides. In Gram-positive isolates, *vanA* was detected in 5% of *E. faecalis* and *ermA* in 10% of *E. faecalis* and 5% of *E. faecium*, correlating with phenotypic resistance to vancomycin and erythromycin, respectively.	[354]
205.	October 2016<br>January 2027<br>March 2017<br>May 2017<br>August 2017	WWTP influent, primary settling tank, secondary settling tank, membrane reactor, and wet well samples	Singapore	*bacA, sul1, sul2, aac, aad, ant, aph, blaLCR, blaAER, blaJOHN, blaGOB, blaIMP, blaOXA, blaKPC, blaPDC, blaPER, cmlA, floR, rosAB, ere, erm, ermF, macAB, msrA, arnA, qepA, arr, mexF, mexW, mexD, mexI, mexT, adeJ, tet36, tet39, tet41, tet43, tet(A), tet(C), tet(E), tet(G), tet(L), tet(Q), tet(R), tet(V), tet(X), dfrAB, vanRSX.*	Illumina HiSeq 2500	The most abundant subtypes of ARGs included bacitracin, sulfonamide, aminoglycoside, class A β-lactamases, multidrug resistance transporters, and macrolide-lincosamide-streptogramin (MLS). In MBR effluent, a broader resistome was identified, aminoglycoside resistance genes, *aac, aad, ant, aph*, bacitracin, *bacA,* β-lactamases, *blaLCR, blaAER, blaJOHN, blaGOB, blaIMP, blaOXA, blaKPC, blaPDC, blaPER,* chloramphenicol resistance, *cmlA, floR*, fosmidomycin resistance, *rosAB,* MLS resistance, *ere, erm, macAB, msrA*, polymyxin resistance, *arnA*, quinolone resistance, *qepA*, rifamycin resistance, *arr*, sulfonamide resistance, *sul1, sul2*, tetracycline resistance, *tet36, tet39, tet41, tet43, tet(A), tet(C), tet(E), tet(G), tet(L), tet(Q), tet(R), tet(V), tet(X)*, trimethoprim resistance, *dfrAB*, and vancomycin resistance, *vanRSX*.	[355]
206.	*N.R.*	Water samples from the Clinton River	U.S.A.: Michigan, Oakland and Macomb Counties	*blaSHV, blaTEM, blaCTX-M*	Culture-based methods/RT-qPCR	Total DNA from river water was analysed by qPCR for three ESBL genes and normalised against the 16S rRNA gene. Copy numbers per mL of river water were 1.19×10^2 for *blaSHV*, 1.21×10^2 for *blaTEM*, and 1.35×10^3 for *blaCTX-M-1*, with the latter being the most abundant gene detected, against a background	[356]

						bacterial density of 8.53×10^4 bacteria/mL.	
207.	*N.R.*	WWTP effluent samples	India: Mangaluru	*blaNDM*-1	RT-qPCR	RT-qPCR revealed *blaNDM-1* gene abundances in effluent samples ranging from 4.1×10^5 to 1.6×10^8 copies/ng DNA. Among the 12 *blaNDM-1*-positive isolates, the highest copy numbers were recorded in three multidrug-resistant *Klebsiella* strains, strain 264K (resistant to 12 antibiotics) had the highest *blaNDM-1* copy number at 1.6×10^8 copies/ng DNA, followed by strain 138K (resistant to 11 antibiotics) at 1.3×10^7 copies/ng DNA, and strain 277K (resistant to 12 antibiotics) at 4.4×10^6 copies/ng DNA.	[357]
208.	September – December 2015	16 potable water samples<br>16 wastewater samples<br>16 sewer samples. All from slaughterhouses	Mexico	*blaTEM, qnrA, qnrB*	Standard PCR	*blaTEM* was the most prevalent ARG in both bacterial and phage isolates, consistent with its globally widespread distribution, particularly among Gram-negative bacteria, and its broad dissemination via mobile genetic elements and migratory waterfowl. In bacterial isolates, quinolone resistance genes *qnrB* (29.7%) and *qnrA* (8.1%) were also detected, with lower prevalence in phage isolates (*qnrB* 10.8%, *qnrA* 2.7%).	[358]
209.	February 2019	Wastewater from a railway train depot – influent and effluent samples	China: Beijing	*blaNDM-1, tet(A), tet(G), qnrA, ermF*	Culture-based methods/qPCR /Illumina MiSeq	*tet(G)* was the dominant ARG across all samples, followed by *qnrA*, while *ermF* and *blaNDM-1* were the least abundant. Significant positive correlations were identified between *intI1* and *tet(A), tet(G), blaNDM-1*, and *qnrA*.	[359]
210.	June 2016 – March 2017	Water samples from ponds and lakes	U.S.A.: California, Los Angeles	*blaIMI-2, blaL1*	Culture-based methods/Standard PCR/Sequencing	All *E. asburiae* isolates carried *blaIMI-2* with 100% identity to the reference sequence, while all 45 *S. maltophilia* isolates harboured *blaL1*, with considerable sequence diversity observed among environmental variants (83–99% identity to the reference *blaL1* gene).	[360]

211.	*N.R.*	Water samples from Yamuna River	India: Delhi	*blaCMY, qnrS.*	Culture-based methods/Standard PCR	Of 73 bacterial strains, 20.5% (n=15) harboured *blaCMY* genes, comprising of two variants: *blaCMY-2* (n=8) and *blaCMY-42* (n=7). Among the PMQR genes screened, *qnrA, qnrB, qnrC, qnrD, qnrS,* and *aac(6')-Ib*, only *qnrS* was detected, identified in 22 bacterial strains, while all other PMQR genes were absent.	[361]
212.	*N.R.*	River water	South Africa: North West Province, Crocodile West and Marico rivers	*blaAmpC*	Culture-based methods/qPCR / dPCR	*AmpC* β-lactamase genes were detected across both rivers, with the Marico River showing higher average abundance (4,656.45 copies/ng DNA) compared to the Crocodile West River (1,859.40 copies/ng DNA). The highest *blaAmpC* gene abundances were recorded at sites 3, 4, and 5 of the Crocodile West River and sites 2 and 5 of the Marico River, while the lowest abundances were found at sites 4 and 6 of the Marico River, and no target *blaAmpC* genes were detected at site 7 of the Marico River.	[362]
213.	September 2018 – October 2019	River samples	Portugal: Lis River	*blaKPC, blaNDM, blaCTX-M, tet(A)*	Culture-based methods/qPCR	*blaKPC* was detected at sites P3, P6, and P9, with absolute abundances of 2.36×10^2 to 1.78×10^3 copies/mL, highest at P3 in both sampling years, and significantly higher relative abundance in 2018. *blaNDM* was detected at all sites except P2 in at least one year, with the highest absolute abundance at P14 and a slightly higher average relative abundance in 2019 (2.06×10^{-4} vs. 8.16×10^{-5} copies/mL). *blaCTX-M* was present at nearly all sites in both years, with a significant overall increase from 2018 to 2019 and the highest absolute abundance consistently at P3. *tet(A)* was the most abundant ARG overall, detected at most sites with absolute abundances ranging from 2.78 $\times 10^4$ to 6.49×10^6 copies/mL, with the highest abundances recorded at P7 (2018) and P2 (2019).	[363]
214.	September 2018 – August 2019	Hospital sewage tanks, WWTP wastewater	Romania: Bucharest, Târgoviște, Râmnicu-Vâlcea, Cluj,	*blaVIM, blaIMP, blaNDM, blaKPC, blaOXA-51/69-like, blaOXA-23-like, blaOXA-24-like, blaOXA-58-like, blaOXA-143, blaOXA-*	Standard PCR/Illumina MiSeq	Across the transmission chain from clinical settings to hospital sewage, WWTPs, and receiving waters, a broad and overlapping spectrum of carbapenemase (CP) and ESBL genes was identified in both *Acinetobacter baumannii* (Ab) and *Pseudomonas aeruginosa* (Pa) strains. In Ab strains, the predominant genes across all	[364]

			Timișoara, Iași, Galați	*235, blaNDM, blaCTX-M, blaTEM, blaSHV, blaPER, blaVEB, blaGES*		settings and years were *blaOXA-23* and *blaOXA-24*, with *blaOXA-23* increasing in hospital sewage from 67.34% (2018) to 96.29% (2019); additional genes detected included *blaVIM, blaTEM, blaSHV, blaGES, blaVEB, blaCTX-M, blaPER*, and *blaNDM*, with *blaNDM* exclusively detected in WWTP Ab strains in 2018 (14.28%) and *blaGES* emerging predominantly in 2019 across hospital sewage (18.51%) and WWTPs (19.87–32.09%). In Pa strains, MBLs dominated, with *blaIMP* and *blaVIM* in clinical isolates, with *blaNDM* emerging in hospital sewage in 2019 (12.5%); ESBLs were represented by *blaVEB*, *blaGES*, and *blaTEM*.	
215.	October and November 2017	WWTP wastewater	South Africa: Eastern Cape Province	*blaCTX-M-2, blaCTX-M-1, blaCTX-M-9, blaTEM, blaSHV, blaIMP, blaOXA-1, blaOXA-48, blaPER, blaVIM, blaKPC, blaGES*	Duplex PCR	Among the β-lactamase genes detected, *blaCTX-M* was the most prevalent (21.5%), comprising *blaCTX-M-2* group (13.8%), *blaCTX-M-1* group (4.6%), and *blaCTX-M-9* group (3.1%), followed by *blaTEM* (20%), *blaSHV* (16.9%), *blaIMP* (12.3%), *blaOXA-1-like* (10.8%), and *blaOXA-48-like* (6.1%). The least prevalent genes were *blaPER*, *blaVIM*, *blaKPC*, and *blaGES* (3.1% each). *blaTEM*, *blaSHV*, *blaCTX-M-2* group, and *blaIMP* were distributed across all identified bacteria.	[365]
216.	May – July 2017	hospital effluent,<br>municipal wastewater pre-entries to the hospital,<br>municipal wastewater post-entry to the hospital<br>14 samples	Ireland	*blaVIM, blaIMP, blaOXA-48 blaNDM, blaKPC*	RT-qPCR	Among 23 carbapenemase-producing Enterobacterales (CPE), *blaOXA-48* was the predominant carbapenemase gene, detected in hospital effluent (n=7), post-hospital wastewater (n=3), and as a co-harboured gene alongside *blaIMP* in two *E. cloacae* complex isolates. *blaKPC* was identified in two hospital effluent isolates and two *C. freundii* from post-hospital wastewater; *blaIMP* in two hospital effluent isolates, one *K. pneumoniae* from post-hospital wastewater, and co-occurring with *blaOXA-48* in two *E. cloacae* complex isolates. *blaVIM* in one hospital effluent isolate and one *K. oxytoca* from post-hospital wastewater, and *blaNDM* was detected exclusively in a single *E. coli* recovered from pre-hospital wastewater. CPE were detected across all sampling sites, with the highest	[366]

						prevalence in hospital effluent (100% of samples), followed by post-hospital (75%) and pre-hospital wastewater (20%).	
217.	January 2017	Lake water	Brazil: Lake Bolonha, Belém, Pará	*blaTEM, blaVIM, blaIMP, blaCTX, blaSHV, blaKPC*	Culture-based methods/Standard PCR	Five β-lactamase gene types were identified across isolates obtained with medium supplemented with cefotaxime and imipenem. *blaCTX* was the most prevalent, detected across *Pseudomonas* and *Acinetobacter* in both media types. *blaIMP* was the predominant carbapenemase, found mainly in BI isolates across *Pseudomonas*, *Acinetobacter*, and *Stenotrophomonas*. *blaVIM* was distributed across multiple genera, including *Pseudomonas*, *Acinetobacter*, *Klebsiella*, *Pantoea*, and *Stenotrophomonas*. *blaTEM* was broadly detected across all major genera in both BC and BI groups; *blaSHV* was identified in *Acinetobacter*, *Pseudomonas*, *Klebsiella*, and *Pantoea*, and *blaKPC* was exclusively found in three *Klebsiella* isolates (BI25, BI39, BI59). Notable co-carriage patterns included *blaCTX* + *blaIMP* + *blaVIM, blaCTX* + *blaTEM* + *blaIMP*, and *blaTEM* + *blaIMP* + *blaVIM.*	[367]
218.	*N.R.*	Surface water samples from Zimapan Dam	Mexico	*blaCTX-M, blaTEM-2*	Culture-based methods/Standard PCR	Of 47 β-lactam-resistant strains, 77.5% were confirmed ESBL producers, with *blaCTX-M* and *blaTEM-2* being the predominant resistance genes detected. *blaCTX-M*-carrying strains were distributed between the pisciculture area (38%) and recreational area (62%), while *blaTEM-2* was similarly distributed (41% and 51%, respectively). Despite significant phenotypic resistance to imipenem and meropenem, no carbapenemase genes were detected in any of the isolates.	[368]
219.	January 2019	16 sediment samples from lakes and rivers	India: Hyderabad	*blaNDM-1*	RT-qPCR	*blaNDM-1* was detected in 13 of 16 sediment samples, with three sites, Durgamcheru lake (S4), Kandi lake (S8), and downstream of Singur dam (S16), testing negative. Among the positive samples, the highest *blaNDM-1* gene copy number was recorded in Amberpet STP sludge.	[369]

220.	2017	Wastewater influent (RWW), secondary wastewater treatment effluent (stww), tertiary wastewater treatment final effluent(ttww), river water, tap water and recycled water samples from the U.S.A.<br><br>RWW and stww of a WWTP in Portugal	U.S.A. and Portugal	*blaOXA-1, blaCTX-M, sul1, sul2, tet(O), gadAB, uidA*	Culture-based methods/qPCR	Among the targeted ARGs, *sul1* and *sul2* and *intI1* were the most abundant and prevalent across all samples, followed by tetracycline resistance genes tet(A) and tet(O), and β-lactam resistance genes *blaOXA-1* and *blaCTX-M*. In US samples, *blaOXA-1, tet(A), tet(O), sul2*, and *intI1* were below the limit of detection, while 23S rRNA, *gadAB, uidA*, and *blaCTX-M* were below the limit of quantification. Secondary treatment led to significant reductions in gene abundances in US samples but not in PT samples, with influent wastewater consistently presenting the highest absolute ARG abundances across both systems.	[370]
221.	*N.R.*	Drinking water sources	Ghana	*ermF, mexB, blaNDM-1, sul1, sul2, tet(G), tet(O), tet(W)*	Culture-based methods/Standard PCR	Across 84 total isolates, *sul1* was the most prevalent ARG (45.98%, n=80), followed by *blaNDM-1* (35.06%, n=61), *mexB* (5.17%, n=9), *sul2* (4.02%, n=7), *tet(G)* (3.45%, n=6), *tet(O)* (2.87%, n=5), and *tet(W)* (3.45%, n=6), while *ermF* was absent in all isolates (0%). *blaNDM-1, sul1, tet(O),* and *tet(W)* were consistently detected across all water sources, with the highest overall ARG burden recorded in the largest sample group (n=36 isolates, 47.70% of total ARGs, n=83). *sul1* and *blaNDM-1* showed the most consistent co-occurrence across all sampling groups, while *sul2* and *tet(G)* were more variably distributed, being absent or rare in smaller sample groups.	[371]

222.	December 2014 – November 2015	River water from different streams of the Po River<br>WWTP samples	Northern Italy: Varzi	*blaCTX-M-1, blaCTX-M-15, blaCTX-M-14, blaCTX-M-28, blaCTX-M-138, blaSHV-12, blaDHA, blaKPC-2, blaKPC-3*	Culture-based methods/Standard PCR	Among 21 ESBL-producing isolates, *blaCTX-M-1* was the most prevalent variant (38%, 8/21), followed by *blaCTX-M-15* (23.8%, 5/21), *blaCTX-M-14* and *blaCTX-M-28* (14.3% each, 3/21), and *blaCTX-M-138* (9.5%, 2/21). *blaCTX-M* co-existed with other β-lactamase determinants in four isolates. *blaSHV-12* was detected in three isolates (*E. coli* 11T, *E. coli* 7T, and *K. oxytoca* 6T), and *blaDHA*-type was identified in a single cefoxitin-resistant *Y. enterocolitica* isolate (11T), while the remaining seven *Y. enterocolitica* strains were negative for all *bla* genes. Carbapenemase genes *blaKPC-2* and *blaKPC-3* were confirmed by PCR and sequencing in *K. pneumoniae* strains 5TP and 9P, respectively.	[372]
223.	February – March 2020	Water samples from rivers and streams	Brazil: São Paulo	*mcr-type, blaCTX-M-2, blaCMY-2, sul1, mdf(A), aaadA1*	Culture-based methods/Standard PCR/Illumina HiSeq 4000	Resistome analysis of strain EW827 identified ARGs spanning seven antibiotic resistance classes: colistin resistance (*mcr-1.1*), β-lactam resistance (*blaCTX-M-2* on a chromosomal Tn21-like element; *blaCMY-2* on the IncI1-ST12 plasmid within the ISEcp1-*blaCMY*-2-blc-sugE context), tetracycline resistance (*tet(A)*), aminoglycoside resistance (*aadA1*), folate pathway inhibitor resistance (*sul1*), phenicol resistance (*catA1*), and macrolide resistance (*mdf(A)*), with *mcr-1.1* and *blaCMY-2* confirmed as transferable via conjugation on the IncX4 and IncI1-ST12 plasmids, respectively.	[373]
224.	2009 – 2019	Biosolids from wwtps	Canada	*aadA1, blaGES, blaVEB, blaOXA-2, ereB, ermB, mefA, qnrB-5, qnrB-8, qnrS, tet(A), tet(B)*	qPCR/Illumina MiSeq	Of 84 targeted ARGs across nine antibiotic resistance classes, 71 were detected with varying abundances. Twelve ARGs were consistently present in all individual samples - aminoglycoside, *aadA1*, β-lactam, *blaGES, blaVEB, blaOXA-2*, macrolide, *ereB, ermB, mefA*, quinolone, *qnrB-5, qnrB-8, qnrS*, and tetracycline, *tet(A), tet(B)* resistance genes. Among macrolide resistance genes, *ermA, ermB, ermC, mefA*, and *msrA* were detected in most samples, with *ermB* recording the highest target copy numbers.	[374]
225.	2015 – 2016	Lentic and effluent	India: Delhi	*blaCTX-M-1, blaCTX-M-15, blaCTX-M-25,*	Culture-based methods/Stand	Among 106 ESBL-producing isolates, *blaCTX-M* was the predominant gene (64%, 68/106), with	[375]

		water samples from the Hauz Khas lake<br>Water samples from Lodhi Gardenpond<br>Effluent of the WWTP near Yamuna River<br>Jasola WWTP samples		*blaCTX-M-205, blaCTX-M-152, blaTEM-116, blaTEM-1, blaSHV*	ard PCR/Sequencing	*blaCTX-M-1* (52/68) and *blaCTX-M-25* (22/68) being the major groups; specific variants identified were *blaCTX-M-15* (70.5%), *blaCTX-M-205* (22.05%), and *blaCTX-M-152* (4.41%). *blaTEM* was detected in 62% of isolates as *blaTEM-116* (ESBL-type) and in 33% as non-ESBL *blaTEM-1*, while *blaSHV* was identified in 17.64% of isolates. Co-carriage of *blaTEM* and *blaCTX-M* was observed in 35.29% of ESBL-producing isolates. Site-wise prevalence of *blaCTX-M* ranged from 57.14% (Hauz Khas lake) to 77.27% (Jasola WWTP).	
226.	September 2011, October 2011, April 2014, June 2014	Influent and effluent samples from UWTP	Poland: Warsaw	*blaTEM, blaOXA, blaSHV, blaCTX-M, blaCMY, blaMOX, blaFOX, blaACT, blaGES, blaPER, blaVIM, blaKPC, blaORN*	Multiplex PCR	Among 128 *bla* genes identified across 24 screened gene types, 13 were detected, *blaTEM, blaOXA, blaSHV, blaCTX-M, blaCMY, blaMOX, blaFOX, blaACT, blaGES, blaPER, blaVIM, blaKPC*, and *blaORN*. *blaTEM* was the most prevalent, identified in 33 strains across all bacterial genera except *Pseudomonas spp.* and *Acinetobacter spp.* Genes consistently detected at all three sampling points were *blaTEM, blaOXA, blaSHV, blaCMY*, and *blaVIM*, with no statistically significant differences in gene type distribution between sampling points.	[376]
227.	April 2017 – March 2018	WWTP samples from Leeuwarden<br>WWTP samples from	Netherlands	*sul1, sul2, tetM, qnrS, ermB, blaCTX-M*	qPCR	Among the selected ARGs quantified in WWTP influents, *ermB* showed the highest average annual concentration (6.39 log gene copies/mL), followed by *sul1* (5.85 log gene copies/mL), while *blaCTX-M* had the lowest concentration (4.05 log gene copies/mL), with similar trends observed across all three WWTPs.	[377]

		Harnaschpolder WWTP samples from Garmerwolde					
228.	July 2017 and July 2018	Hospital outflow samples	Slovenia and Austria	*blaCTX-M, blaVIM, blaKPC, blaNDM-1, blaOXA-48*	Culture-based methods/RT-qPCR	*blaCTX-M* was the dominant ESBL, with *blaCTX-M-1* detected in 14 isolates across *E. coli*, *C. freundii*, *K. pneumoniae*, *P. mosselii*, and *B. subtilis*, and *blaCTX-M-9* in five isolates (*C. freundii*, *E. faecium*, *E. coli*). *blaOXA-48* and *blaVIM* were each detected in 10 isolates across multiple species, while three isolates co-harboured *blaVIM* + *blaCTX-M* and *blaCTX-M* + *blaOXA-48*. *blaKPC* and *blaNDM* were not detected in any isolate. *blaOXA-48*-producing CPE were present at the lowest concentrations across all sampling sites, with *blaOXA-48*-type *E. coli* detected at 1×10^5 cfu/mL in the Austrian private surgery clinic in one year but absent in 2018.	[378]
229.	October 2014	Water samples from Água Preta Lake	Brazil: Pará	*blaTEM, blaSHV,* *blaCTX−M, blaIMP, blaVIM, blaKPC*	Culture-based methods/Standard PCR/Sequencing	*blaCTX-M* was the most prevalent β-lactamase gene (n=12), followed by *blaTEM* (n=5) and *blaSHV* (n=4), while carbapenemase genes *blaIMP*, *blaVIM*, and *blaKPC* were absent in all isolates. Among *blaCTX*-M variants, *blaCTX-M-15* was the most common (n=5, in *Klebsiella*, *Escherichia*, and *Shigella*), followed by *blaCTX-M-14* (n=1, *Escherichia*) and *blaCTX-M-2* (n=1, *Shigella*). Notably, 10 of 22 third-generation cephalosporin-resistant isolates were negative for *blaCTX-M*, suggesting alternative resistance mechanisms in isolates such as *Acinetobacter*, *Pseudomonas*, and *Enterobacter*.	[379]
230.	17 March 2017	Surface water samples from upstream	India: Cuttack city	*blaKPC, blaNDM, blaVIM, blaIMP, blaOXA-48-like,*	Culture-based methods/Standard PCR	Among 70 carbapenem-resistant *K. pneumoniae* (CRKP) isolates, *blaNDM* was the predominant carbapenemase gene, detected at 100% prevalence at sites K3 and K4, and at 92.3% and 57.14% at K2 and K5, respectively. *blaOXA-48*-like was	[380]

		and downstream of the Mahanadi River<br>River water samples from upstream Cuttack city<br>2 water samples from the city's main drainage system		*blaTEM, blaSHV, blaCTX-M*		identified across all sites (7.69–57.14%), while *blaKPC* was detected at K3 (21.42%) and K2 (7.69%), and *blaVIM* in a single isolate at K2. Most CRKP isolates co-harboured ESBL genes, with *blaTEM* and *blaSHV* present in all isolates, and *blaCTX-M* detected predominantly at K3 (50%), followed by K5 (28.57%), K2 (23.07%), and K4 (22.72%).	
231.	13 May and 14 May 2019	7 canal water samples<br>3 streams and springs water samples<br>2 pond water samples<br>1 wwtp sample<br>5 small lake and sewer water samples	Northern Italy: Pavia	*blaCTX-M, blaVEB, blaKPC, blaOXA, blaMOX, aac(6')-Ib-cr, qnrS*	Culture-based methods/Standard PCR/Sequel I sequencing	Among 30 ESBL-producing Enterobacterales isolates, *blaCTX-M*-type, predominantly *blaCTX-M-1*, was the most prevalent gene (80%, n=24), followed by *blaOXA-244* (13.3%, n=4), and *blaKPC-2*, *blaMOX-2*, and *blaVEB-1* (3.3% each). *blaCTX-M* co-existed with *blaOXA-244* in four *E. coli* isolates and with *blaKPC-2* in one *K. pneumoniae* isolate. Additionally, *blaCTX-M* co-occurred with *aac(6')-Ib-cr* in 45% and with *qnrS* in 15% of isolates, while one *E. coli* isolate co-harboured *blaVEB-1* and *blaMOX-2*.	[381]
232.	July 2015 – April 2016	Water samples from	U.S.A.: Texas	*tetA, tetW, blaAmpC, blaTEM, mecA, aacA, ermA*	Culture-based methods/Standard PCR/qPCR	ARG abundances were significantly higher at downstream sites compared to upstream locations, with *blaTEM*, *ermA, intI1, tet(A),* and *tet(W)* all	[382]

		Carters Creek and Burton Creek				significantly elevated. *ermA* showed the most pronounced enrichment, averaging 4.3 × 10^5 copies/100 mL downstream, 48-fold higher than upstream (9 × 10^3 copies/100 mL), peaking at site 4 (6 × 10^5 copies/100 mL). *blaTEM* was significantly greater downstream, with site 4 recording 7.33 × 10^4 vs. 9.6 × 10^3 copies/100 mL upstream, while aacA showed a similar but non-significant trend. All ARGs were consistently detected across all sites and sampling events, with the exception of *mecA, aacA, emrA*, and *blaTEM* at specific sites and sampling events.	
233.	June and November 2018	Sewage, water and sludge samples from WWTPs	Poland: Warmia and Mazury regions	*blaTEM, blaOXA, blaSHV,*	Standard PCR	*blaTEM* was detected in all wastewater samples from both WWTPs in June and November, including treated wastewater and sludge. *blaOXA* was present at all treatment stages in the Silesia WWTP in both months, detected in sludge only in November, but absent in downstream river water at the Warmia and Mazury WWTP. *blaSHV* was not detected in any June samples from Silesia, whereas in November, it was detected in downstream river water but was effectively removed by the Silesia WWTP. *blaSHV* persisted across nearly all treatment stages at the Warmia and Mazury WWTP in both sampling months.	[383]
234.	*N.R.*	Water samples from 32 abattoirs	Iran: Mazandaran Province	*blaSHV*	Culture-based methods/Standard PCR	Of 15 *E. coli* isolates, 93.33% (n=14) were phenotypically ESBL-positive, with molecular analysis confirming *blaSHV* in 93.34% (14/15) of isolates, while one isolate (6.66%) tested negative for *blaSHV*.	[384]
235.	December 2018	Surface water samples of Ganga, Gomti, Yamuna, and Hindon rivers	India	*blaCTX-M, blaTEM,* *blaSHV, blaOXA, blaCIT,* *blaDHA, blaACC, blaEBC, blaMOX, blaFOX*	Culture-based methods/Multiplex PCR	*blaTEM* was the most prevalent ESBL gene (96.84%), followed by *blaCTX-M* (18.95%), *blaSHV* (27.37%), and *blaOXA* (13.68%). Among *blaAmpC* producers (7.46% by genotypic analysis), the following *blaAmpC* genes were identified across 10 isolates: *blaACC* (60%), *blaDHA* (30%), *blaCIT* (30%), *blaFOX* (20%), *blaEBC* (20%), and *blaMOX* (10%).	[385]

236.	*N.R.*	Enterobacteriaceae isolates obtained from water samples from Wester *Black* Sea and Lake Manyas strains	Turkey	*blaTEM, blaSHV, blaCTX-M, blaCIT*	Standard PCR	PCR analysis identified *blaTEM* in three *E. coli* isolates (B89, B91, B120), *blaSHV* in six *E. coli* (B120, B176, B180, B185, B233, B235) and one *K. pneumoniae* (M55), *blaCTX-M* in three *E. coli* (B236, B237, B243), and the *blaAmpC*, *blaCIT* gene group in two *E. coli* (B185, B243); *blaIMP, blaMOX*, and *blaFOX* gene groups were absent in all isolates. Co-carriage of multiple genes was observed in three strains: B120 (*blaTEM + blaSHV*), B243 (*blaCTX-M + blaCIT*), and B185 (*blaSHV + blaCIT*).	[386]
237.	June, November 2018<br>March 2019	Water samples from WWTP in region of Warmia and Mazury<br>Water samples from WWTP in region of Silesia	Poland	*blaGES, blaIMP, blaKPC, blaNDM, blaVIM, blaOXA, blaOXA-23, blaOXA-24, blaOXA-48, blaOXA-58, blaCfxA*	Illumina TruSeq	The dominant β-lactamase genes across both WWTPs were *blaOXA, blaGES, blaBEL, blaCfxA*, and *blaTEM*, with no statistically significant differences in abundance between plants or between raw and treated wastewater. The most abundant carbapenemase genes were *blaGES* and *blaOXA* subtypes, *blaOXA-23, blaOXA-24*, and *blaOXA-48*. Notably, activated sludge treatment increased the relative abundance of several genes, such as *blaOXA* (2.7-fold) and *blaBEL* (4.5-fold) in WM-WWTP, and *blaTEM* (2.9-fold) and *blaBEL* (5.7-fold) in S-WWTP. *blaNDM* and *blaKPC*, detected only in WM-WWTP influent, were eliminated after mechanical treatment, while *blaIMP* increased following biological treatment in both plants (WM-WWTP: 0.24% to 4.27%; S-WWTP: 0.00% to 5.24%).	[387]
238.	January – March 2021	5 water samples from Douro River	Portugal: Vila Nova de Gaia	*blaKPC, blaOXA-48, blaNDM, blaVIM, blaIMP, blaCTX-M, blashv.*	Culture-based methods/Multiplex PCR	All 15 carbapenemase-producing isolates confirmed by CIM harboured *blaKPC* as the sole carbapenemase gene, with no other carbapenemase genes detected. Additionally, all isolates co-carried one to three ESBL genes: *blaCTX-M-2* (46.7%, n=7), *blaTEM* (40%, n=6), and *blaOXA* (26.7%, n=4), while *blaCTX-M- 9* and *blaSHV* were absent in all isolates.	[388]

239.	2013 – 2014 September and November 2015	Influent and effluent samples from Waste Stabilizatio n Ponds (wsps) from Pond Inlet and Clyde River Influent and effluent samples from WWTP in Iqaluit	Canada: Baffin Island, Qikiqtani Region, Nunavut	*blaCTX-M, blaTEM, erm(B), qnrS, sul1, sul2, tet(o), mecA*	Culture-based methods/qPCR	Across all three WWTPs, ARG abundances were consistently higher in influent compared to effluent samples, with *ermB* and *qnrS* among the most abundant genes detected across all sites. At Iqaluit's WWTP, no significant differences were observed between influent and effluent for any ARG, including *blaCTX-M, intI1, blaTEM, sul1, sul2, mecA, tetO, qnrS*, and *ermB*. At Clyde River's WWTP, *blaCTX-M, intI1, blaTEM, sul1, sul2, mecA, tetP, qnrS*, and *ermB* were detected in both influent and secondary treatment effluent, with significant reductions observed for several ARGs following secondary treatment, and no significant seasonal or annual differences noted. At Pond Inlet's WWTP, the same ARGs were quantified in influent, with significant reductions in ARG content following treatment.	[389]
240.	2017 – 2019	Water samples from 3 Recycled water treatment plants (RWTPS)	Australia	*blaNDM, blaOXA-48-like, blaVIM, blaIMI, blaSME, blaKPC, mcr-1, mcr-3, qnrS*	RT-qPCR	Among the targeted ARGs, *blaNDM, blaOXA-48-like, blaVIM, blaIMI,* and *blaSME* were not detected in influent or treated recycled water at any of the three RWTPs, while *blaTEM, blaGES, blaKPC*, and *blaSHV* were repeatedly detected, with *blaSHV* reduced below the limits in final recycled water from RWTPs B and C. *qnrS* was the most abundant ARG across all plants (up to 1.7×10^{10} copies/mL influent), and *mcr-1* was consistently detected across all three plants (2.7×10^{2} to 1.3×10^{6} copies/mL influent), while *mcr-3* remained below detection.	[390]
241.	February and March 2020	Hospital influent and effluent water samples	Mexico: Mexico City	*blaCTX-M, blaSHV, blaAMPc, blaGES-2, blaKPC, blaOXA-48-like, blaNDM-1, blaIMP*	Culture-based methods/Stand ard PCR	PCR molecular analysis identified *blaKPC* in one *E. coli* isolate and *blaCTX-M* in another, with two additional isolates co-harbouring more than one β-lactamase gene.	[391]
242.	*N.R.*	Untreated graywater (GW) from households	Israel: Bedouin villages	*sul1, qnrS, blaCTX-M-32, blaTEM*	Culture-based methods/qPCR	Among all the ARGs quantified by qPCR in GW samples, *intI1* was the most abundant across all villages (1.4×10^{-1} gene copies/16S rRNA), followed by *sul1* (5.2×10^{-2} copies/16S rRNA), *qnrS* (1.7×10^{-2} copies/16S rRNA), *blaTEM* (3.5	[392]

						× 10^{-3} copies/16S rRNA), and *blaCTX-M-32* (2.2 × 10^{-5} copies/16S rRNA). Significant positive correlations were identified between *qnrS* and both *intI1* and *sul1* (p=0.03), between *blaTEM* and both *blaCTX-M-32* (p=0.01) and *qnrS* (p=0.0001), and between *intI1* and *sul1*, suggesting co-selection and potential co-dissemination of these ARGs via mobile genetic elements in groundwater environments.	
243.	April 2013 – March 2014	Water samples from the La Paz River<br>Soil samples from Mecapaca river shorelines	Bolivia: La Paz	*blaTEM, blaCTX-M-3, blaOXA-1, tet(A), sul1, sul2, qnrS1, qnrB1*	Culture-based methods/RT-qPCR/Illumina MiSeq	Of 101 isolates, five were ESBL-positive, and no carbapenemase genes were detected by PCR. The five positive isolates carried multiple ARGs, including β-lactam resistance genes, *blaTEM, blaCTX-M-3, blaOXA-1*, tetracycline resistance, *tet(A),* sulfonamide resistance, *sul1, sul2*, and quinolone resistance, *qnrS1, qnrB1*.	[393]
244.	April – October 2023	Surface water samples from the Burr Oak (BO) creek	U.S.A.: North Iowa	*aph(3'')-Ib, aph(6)-Id, aph(3')-Ia, aadA2, aac(6')-Ib-cr, blaTEM-34, blaTEM, blaCTX-M-15, blaCTX-M, blaOXA-1, blaNDM-5, catB3, sul1, sul2, tet(A), dfrA12, dfrA, mdf(A), mph(A).*	Culture-based methods/Standard PCR/Illumina MiSeq	Genetic analysis of NDM-5 producing *E. coli* strain BO1 identified 15 ARGs spanning six resistance classes: aminoglycosides, *aph(3'')-Ib, aph(6)-Id, aph(3')-Ia, aadA2, aac(6')-Ib-cr*, β-lactams, *blaTEM-34, blaCTX-M-15, blaOXA-1, blaNDM-5*, phenicols, *catB3*, sulfonamides, *sul1, sul2*, tetracycline, *tet(A)*, and trimethoprim, *dfrA12*. Among clinical strains, carrying 8–19 ARGs each, the most prevalent genes were *dfrA* and *sul1* (n=13 each), followed by *aadA2* (n=12), *mdf(A)* (n=11), *blaTEM* and *tet(A)* (n=9 each), *mph(A)* (n=8), *blaCTX-M* and *sul2* (n=6 each), and several additional ARGs detected in one to five isolates.	[394]
245.	July – August 2021	Hospital effluent samples	China: Shanghai	*BlaVIM, blaOXA-48, blaKPC, blaNDM, blaTEM, fosB, fosM1, fosA6, oqxB, tet(A), mcr-8.1*	Culture-based methods/Illumina NovaSeq 6000	Several identified ARGs were exclusive to specific hospital tiers: *blaVIM-4/15/23/29/32* were restricted to tertiary hospitals, *mcr-8.1* to secondary hospitals, and *blaOXA-48* and *blaVIM-48* to primary hospitals, while *blaKPC*-8, *blaNDM*-1/2/3/5/6/7/9/10/12/14/15/17/19, *fosB*,	[395]

						and *fosM1* were unique to private healthcare institutions. Among the 62 core ARGs shared across all ten geographical districts, notable geographic variation in abundance was observed, with *tet(A)*, *fosA6*, and *oqxB* predominating in the Jinshan district, and *blaTEM-1* and *blaTEM-192* more prevalent in the Pudong district.	
246.	*N.R.*	Samples from tertiary treatment processes ff WWTP	Australia: Melbourne	*blaTEM, aac(6')-Ib-cr, drfA1, sul1, sul2, mefA, ermb, tetA, tetB, tetX*	qPCR/Illumina MiSeq	Nine of 10 targeted ARGs, all except *tetB,* were detected as intracellular ARGs (iARGs) in secondary effluent (SE), ranging from 5.86 × 10^3 to 5.86 × 10^7 gene copies/L, with *sul1* being the most abundant and *blaTEM* the least. Ultrafiltration (UF) significantly reduced all iARG concentrations by 0.70–3.37 log (overall 1.94 log, 98.86% reduction), achieving complete removal of *blaTEM*. For extracellular ARGs (eARGs), all 10 ARGs were detected in SE (3.50 × 10^3 to 9.46 × 10^6 gene copies/L), with UF achieving a lower overall reduction of 0.60 log (74.87%). *aac(6')-Ib-cr, sul2, mefA, tet(A),* and *tetB* were significantly reduced (0.44–1.57 log), while *blaTEM* and *ermB* were only slightly reduced (0.02–0.06 log), confirming that UF is more effective at removing iARGs than eARGs.	[396]
247.	June – December 2020	Surface water samples from around two pharmaceutical industries	Bangladesh: Savar area	*blaTEM, blaSHV, blaCTX-M-15, blaCTX-M-13*	Culture-based methods/Standard PCR/ABI Prism 310 Genetic Analyser	Among 51 *E. coli* isolates, 69% (n=35) were confirmed ESBL-positive. Four β-lactamase genes were detected: *blaTEM* (70.6%, n=36), *blaCTX-M-13* (60.8%, n=32), *blaCTX-M-15* (54.9%, n=28), and *blaSHV* (1.96%, n=1).	[397]
248.	January – November 2021	Hospital wastewater	Japan: Kisarazu City	*blaCTX-M, blaCTX-M-14, blaCTX-M-27, blaCTX-M-8, blaCTX-M-27, blaCTX-M-55, blaDHA-1*	Culture-based methods/Standard PCR/Illumina HiSeq X Five	All 140 ESBL-producing *E. coli* isolates harboured *blaCTX-M* group genes, with *blaCTX-M-14* being the predominant variant (n=132, 94.3%), followed by *blaCTX-M-8* (n=6) and *blaCTX-M-27* (n=2). The vast majority of *blaCTX-M-14*-positive isolates (128/132) belonged to the pandemic B2-O25b: H4-ST131-H30R/non-Rx sublineage, detected across January,	[398]

						May, and July sampling events. In September and November, *blaCTX-M* group genes and the ST131 lineage were absent, replaced by *blaDHA-1*-carrying isolates predominantly associated with clade I-O8:H33-ST3910-fimH1074 (26/28 isolates) and B2-O25b: H4-ST131-fimH41 (3/3 isolates), indicating a seasonal shift in both the dominant ARG and the associated clonal lineages in hospital wastewater.	
249.	February 2019 – February 2020	Water samples from rivers such as Evros, Axios Aliakmonas, Gallikos, Strymonas, Nestos, and Vosvozis	Greece	*blaCTX-M-1, blaCTX-M-2, blaCTX-M-9, blaOXA-1, blaSHV, blaTEM*	Culture-based methods/Multiplex PCR/Illumina NovaSeq 6000	Among 87 ESBL-producing *E. coli* isolates, 93.1% harboured at least one of seven β-lactamase gene families, with 63.2% (55/87) carrying more than one gene. *blaOXA* was the most prevalent (69%, 60/87), followed by *blaCTX-M-1* group (55.2%, 48/87), *blaCTX-M-9* group (25.3%, 22/87), and *blaTEM* (17.2%, 15/87), while *blaCTX-M-2* and *blaSHV* were not detected. The colistin resistance gene *mcr*-1 was identified in the single colistin-resistant isolate.	[399]
250.	September 2021 – June 2022	Influent and effluent samples from 4 wwtps	Spain: Seville	*blaKPC-2, blaOXA-48, blaVIM-1, blaCTX-M-1, blaCTX-M-9, blaCTX-M-15, blaTLA-4, blaVEB-1.*	Culture-based methods/Illumina MiSeq	Among 152 ESBL/carbapenemase-producing isolates, *blaKPC-2* was the most prevalent carbapenemase, followed by the *blaOXA-48* group. Eight isolates co-harboured both *blaKPC*-2 and *blaOXA-48*, and three additionally carried ESBL genes. Two *blaVIM-1*-producing isolates were identified, one carrying *blaVIM-1* alone and the other co-harbouring *blaVIM-1*, *blaOXA-48*, and *blaCTX-M-15*. Among ESBL producers, *blaCTX-M-1* group was the primary determinant (36.1%, n=35), followed by *blaCTX-M-9* group (15.5%, n=15), with two isolates co-producing both clinical and environmental ESBL genes, *blaCTX-M-15 + blaTLA-4; blaCTX-M-15 + blaVEB-1*.	[400]
251.	January 2022 – March 2024	Sewage samples in 33 access points	Japan	*blaCTX-M-14, blaCTX-M-27, blaCTX-M-15, blaCTX-M-24, blaCTX-M-55*	Culture-based methods/Illumina MiSeq	Among ST131 isolates, *blaCTX-M-27* was the predominant gene (15/21), followed by *blaCTX-M-15* (3/21), *blaCTX-M-14* (2/21), and *blaCTX-M-55* (1/21), while ST38 isolates were predominantly characterised by *blaCTX-M-14*	[401]

						(16/21), followed by *blaCTX-M-15* (3/21), *blaCTX-M-24* (1/21), and *blaCTX-M-27* (1/21). Overall, *blaCTX-M-14* was the most prevalent ESBL gene across all isolates (39.0%, 23/59), followed by *blaCTX-M-27* (28.8%, 17/59) and *blaCTX-M-15* (15.3%, 9/59).	
252.	September 2022 to August 2023 July 2024	Wastewater influent samples<br>122 samples	U.S.A.: Milwaukee, Wisconsin	*blaKPC, blaOXA-40/24-like, blaOXA-48-like duplex, blaVIM, blaIMP, blaNDM*	Culture-based method/ddPCR	The culture of select media has shown a proliferation of *Klebsiella pneumoniae, Escherichia coli, and Acinetobacter baumannii* The analysed genes were identified in all samples taken from both WWTPs. These genes showed extremely high abundance *blaKPC* (3.55E+06 cn/L), *blaOXA*-24/40 (1.11E+06 cn/L), *blaOXA*-48 (4.08E+05 cn/L) and *blaVIM* (1.58E+05 cn/L). The *blaIMP* and *blaNDM* was detected in most samples but at lower concentrations (1.13E+04 cn/L), respectively (1.05E+03 cn/L).	[402]
253.	N.R.	Water from tap, underground well, stream, spring, and borehole<br>180 samples	Nigeria	*blaCTX*-M, *blaSHV*, and *blaTEM*	Culture-based method/PCR	The twenty-five *E. coli* isolates obtained from water samples showed ESBL resistance genes. The *blaTEM* showed increased abundance (44%), while the *blaSHV* was identified in a lower abundance (16%). The *blaCTX-M* gene has not been identified in any evidence.	[403]
254.	February 2019 to February 2020	Wastewater samples<br>316 samples	Japan: Sendai, Miyagi prefecture	*aac*(6')-Ib, *qnrA, qnrB, qnrC, qnrD*, and *qnrS*, *blaCTX*-M, *blaTEM*, *blaSHV*	PCR	The majority of ESBL-EC isolates (98.1%) showed *blaCTX*-M, with the highest abundance (62.3%). In addition, strains carrying multiple *blaCTX-M* groups were identified. *blaTEM* was detected in 27.5%, while *blaSHV* universal was not detected in any of the isolates.	[404]
255.	February 2020 June 2020	Groundwater, rainwater,	Slovenia	*blaSHV*, *blaTEM*, and *blaCTX-M*, *blaKPC*, *blaOXA*-48, *blaVIM*, *blaIMP*,	Culture-based method/ ERIC-PCR	Among the isolates analysed, 37.5% were *Pseudomonas spp.*, followed by *Stenotrophomonas spp.* (21.7%) and *Aeromonas spp.* (10.8%). Enterobacteriaceae accounted for	[405]

		and leachate<br><br>8 samples		and *blaNDM-1* *blaKPC*, *blaNDM*, *blaVIM*, *blaIMP*, *bla*OXA-48 like, *blaMCR*, *blaGES*, *blaGIM*, *blaSPM*, *bla OXA-23 like*, *blaOXA-24/40 like*, and *blaOXA-58 like*	NextSeq 2000 Illumina	7.9% of all identified isolates, including *Klebsiella*, *Citrobacter*, *Enterobacter*, *Escherichia*, and *Raoultella*. In the tested samples, two ESBL-producing isolates and two CP-producing isolates were identified (*Klebsiella oxitoca* carrying the *blaCTX-M-1* gene and the *blaOXA-48* gene, *Serratia fonticola* carrying the FONA-2 gene, and *Pseudomonas aeruginosa* VIN gen.	
256.	2023 –2024	Water samples	China: Henan Province, Yellow River	*blaCTX-M-G9*	qPCR/ Illumina Nova Seq PE150 platform	The *blaCTX*-M-G9 gene was identified in all samples taken, with greater abundance in the dry season (10^{-5} -10^{-3} copies/16S rDNA) compared to the rainy season (10-^{6}-10^{-4}copies/16S rDNA)	[406]
257.	December 2020 to October 2021	Wastewater samples River water<br><br>36 samples	Japan: Matsumoto City	*blaIMP, blaNDM, blaKPC, blaGES*, and *blaOXA*-48	PCR/WGS	The dominant species *Aeromonas caviae* (33.3%) was followed by *Enterobacterales* (*Klebsiella ssp., Raoultella ssp.).* All 18 bacterial species identified in this study showed an isolated gene *blaGES* (96%). Also, *blaGES-24* and *blaIMP-1* were identified in two isolates (96%).	[407]
258.	August 2020 and April 2021	Raw hospital wastewater, inflow and outflow from the mwwtps, and river water 242 samples	Czech Republic	*blaGES*, *blaIMI*, *blaNMC*−A, *blaIMP*, *blaKPC*, *blaNDM*, *blaOXA48*−like, *blaVIM*	Culture-based method/ PCR/HiSeq 4000, Illumina	The *blaCTX*−M−15 was identified in the majority of isolates (99.7%). In addition, *blaGES*−51 (n = 10), *blaOXA*−48 (n = 4), *blaNDM*−1 (n = 4), and *blaKPC*−3 (n = 1) were detected in all the isolates obtained from the samples collected, except those from river water. The *mcr* gene was not identified in any isolate, but the *oqxA/oqxB*, *sul2*, *aph(6)-Id*, *dfr14*, *qnrB1*, and *aac(6')-Iboscones* genes were also identified.	[408]
259.	May to September 2019	Wastewater and<br><br>Sludge 24 samples	Iran: Qom	*BlaCTX-M-4, blaCTX-M-32, blaTEM-1, blaVIM-4, blaIMP-2,blaAmpC, blanps-1, blaoxa-1, mecA*	RT PCR	All the genes analysed in this study, except *blaCTX-M-4* and *blaVIM-4*, were present at all locations analysed. The most common genes identified at all sampling points were *blaCTX-M-32* (4.81E+03 copy/100 mL) and *blanps-1* (5.16E+02 copy/100 mL), followed by *blaAmpC* and *blaOXA-1* (9.74E+01 copy/100 mL) genes. The *blaIMP-2*, *mecA*, and *blaTEM-1* genes were less abundant in wastewater. In the in the primary	[409]

						sludge step, the highest amount was the gene *blaCTX-M-32* (5.54E+03 copy/100 mL), followed by the *blanps-1*(6.03E+02 copy/100 mL)and *blaOXA-1*.	
260.	March and August 2024, September–October 2024	Wastewater<br>55 samples	Sweden: Kristianstad	*blaNDM, blaOXA*-48-like	PCR/Illumina NovaSeq X+	A total of 18 isolates were identified, specifically *E. coli* (10) and *K. pneumoniae* (8). The analyses indicated the presence of the *blaNDM* genes (94%)and the *blaOXA-48* genes (56%).	[410]
261.	November 2022 – September 2023	Water samples	São Tomé and Príncipe: São Tomé	aac(3)- Iia, aac(3)- Iid, aac(6')- Ib-cr, aac(6')- Ib3, aadA1, aadA2, aadA5, aph(3")- Ib, aph(6)- Id, *bla*CMY-2, *bla*CTX- M-1, *bla*CTX-M-14, *bla*CTX- M-15, *bla*CTX- M-27, *bla*CTX- M-55, *bla*OXA-1, *bla*TEM-122, *bla*TEM- 135, *bla*TEM- 141, *bla*TEM-1A, *bla*TEM- 1B, *bla*TEM- 1C, *bla*TEM-209, *bla*TEM- 29, *bla*TEM- 30, *bla*TEM-55, *bla*TEM- 5	MinION Mk1c platform	*E.coli*<br>*The most common genes identified in the isolated samples were blaCTX-M-15* (80%, sul2 (54%), *bla*TEM-1B (36%) and *bla*OXA-1 (29%). The remaining genes analysed had frequencies of 4% or less.	[411]
262.	2022	Surface water	Belgium: Wallonia	*bla*OXA-48, *bla*KPC, *bla*NDM, *bla*VIM, and *bla*IMP	Culture-based method/ RT-PCR/WGS	*E. coli* and *K. pneumoniae* were isolated in surface waters. In *E. coli* isolates, the high abundance showed the *blaKPC* (83.3%) followed by the *blaOXA*-48 (16.6%) gene, and in *K. pneumoniae*, the *blaKPC* (50.0%), *blaOXA*-48 (940.9%), *blaNDM* (4.5%) and *blaVIM* (4.5%).	[412]
263.	June 2024 – March 2025	Wastewater influents	Japan: Kanto Prefecture, Kinki Prefecture	*blaCTX-M, blaCTX-M-1, blaCTX-M-2, blaCTX-8, blaCTX-M-9, blaCTX-M-25, blaGES, blaSHV and blaTEM, intI1*	qPCR	*E. coli* abundances were 8.9×10^3–5.1×10^4 CFU/mL. The *E. coli* isolates exhibited a broad spectrum of the *blaCTX*-M-1(42%), *blaCTX*-M-9 (17%), and *blaTEM* (8%) genes. *blaSHV* and *blaTEM* were consistently detected in all wastewater samples collected from the two locations; in	[413]

						contrast, *blaGES* was detected more frequently in the wastewater from City A.	
264.	*N.R.*	Soil and manure samples and water sampels from organic farms	India	*blaTEM, blaAmpC, blaCTX-M-9*	Culture-based methods/Standard PCR	Of the 11 *Salmonella* isolates, *blaCTX-M-9* was detected exclusively in the three *S. Miyazaki* isolates, two of which also showed phenotypic resistance. No isolate carried *blaTEM* or *blaAmpC*. The *blaCTX-M-9* gene was uniformly present across all *S. Miyazaki* isolates. Additionally, *S. Virchow* and *S. Infantis*, both recovered from water samples, displayed multidrug-resistant phenotypes.	[414]
265.	January – December 2019	WWTP influent and effluent samples	Algeria	*blaCTX-M, blaOXA-58, blaTEM, blaIMP, blaKPC, qnrS, sul1, tetO*	Culture-based methods/qPCR	*blaIMP, blaTEM, blaCTX-M, blaKPC, blaOXA-58, sul1, tetO,* and *qnrS* were detected across all samples, with their distribution during wastewater treatment generally tracking total bacterial abundance. By comparison, ARG abundance ranged more widely, from 1.4 to 5.8 log-units in influent samples and 3 to 5.8 log-units in effluents, suggesting that ARG removal did not consistently mirror the reduction seen in total bacterial load.	[415]
266.	May, October and December 2015	WWTP Wastewater	Costa Rica	*blaOXA, blaTEM, sul1, sul2, tetA*	Culture-based methods/ ERIC-PCR	Four of the five target resistance genes were detected in the isolates, *blaOXA* being the exception. Genotypic profiling identified 18 distinct resistance gene profiles among the multidrug-resistant isolates, with the combination of *blaTEM, sulI, sulII, intI1*, and *tetA* emerging as the most prevalent, particularly in effluent samples from WWTP 1. Of the 30 isolates, 26 displayed unique phenotype-genotype combinations. At the gene level, *blaTEM* was the most widely distributed, present in 19 isolates, of which 16 also carried sulI, 16 carried *sul2*, 14 carried *tetA*, and 15 carried at least one *intI* gene. In comparison, five isolates carried a *sul* gene without an accompanying *intI* gene, with no notable differences in gene distribution between influent and effluent samples.	[416]

267.	14 October 2024	Hospital wastewater	Indonesia: Jakarta	*blaTEM-1B, blaCTX-M-15, blaCMY-2, aac(6')-Ib-cr, qnrS1, qnrS13, qacH, mdtF, acrS, mdtH, mdtM, gyrA, parC, pare, aac(3)-IIA, aadA5, aph(6)-Id, tet(A), catA2, catB3, sul1, sul3.*	Culture-based methods/Illumina NextSeq 2000	A total of 57 AMR genes were identified across the five *E. coli* strains, with β-lactam resistance genes predominating, four strains carried either *blaTEM-1B* or *blaCTX-M-15*, consistent with their ESBL phenotypes, while two strains harbored *blaCMY-2*, a plasmid-mediated *blaAmpC* β-lactamase gene.	[417]
268.	*N.R.*	Hospital wastewater	Philippines: Manila and Quezon City	*blaCTX-M-1, blaCTX-M-9, blaCTX-M-15, bla-CTX-M-27, blaTEM-1*	Standard PCR/Sanger sequencing	Sanger sequencing of PCR amplicons from ESBL-positive *E. coli* isolates produced 17 high-quality sequences, comprising nine *blaTEM-1* genes (52.94%) and eight *blaCTX-M* genes (47.06%), among the latter, six were identified as *blaCTX-M-15*, belonging to the *blaCTX-M-1* group, and two as *blaCTX-M-27*, belonging to the *blaCTX-M-9* group.	[418]
269.	March 2022 – June 2023	Domestic and hospital wastewater 23 water samples	India: Aligarh	*blaCTX-M, blaSHV, blaNDM-1*	Standard PCR	PCR analysis revealed that among the confirmed metallo-β-lactamase (MβL) producers, 11 isolates (73.33%) carried the *blaCTX-M* gene, 6 (46.15%) carried *blaSHV*, and 7 (53.84%) carried *blaNDM-1*.	[419]
270.	June 2024	Drinking water (Well-water, borehole-water and sachet-packaged water) 45 samples	Nigeria: Katsina	*blaNDM*, *blaVIM*, *blaIMP*, *qnrA*, *parC*	Culture-based methods/Standard PCR	Of the 27 MDR isolates recovered, 13 were selected for targeted ARGs analysis, including all 9 isolates that were both MDR and phenotypically carbapenemase producers, plus 4 MDR isolates resistant to fluoroquinolones. These isolates originated from well water (n=5), borehole water (n=5), and sachet water (n=3). Gene distribution varied by water source; *blaNDM* and *parC* were detected exclusively in well water isolates, while *blaVIM* and *qnrA* were found only in borehole water isolates, and no targeted carbapenem or quinolone resistance genes were detected in sachet-packaged water. Overall, ARGs were more prevalent in well and borehole water than in sachet water, with 20% of borehole isolates carrying *blaVIM* and *qnrA*, 20% of well water isolates carrying *blaNDM*, and 40% carrying *parC*. Separately, carbapenemase production was assessed across 32 isolates from five sampling locations, with an overall prevalence of 28.1%,	[420]

						ranging from 0% in Kofar Kwaya to 9.3% in Kofar Marusa.	
271.	June 2022 – February 2023	Influent and effluent wastewater, pharmaceutical WWTPs, influent and effluent from one municipal WWTP, untreated municipal wastewater, and river water 49 samples	Nepal: Kathmandu	*blaTEM, blaCTX-M, blaSHV, qnrA, qnrS, aac(6')-Ib-cr, aadA9, ermB, sul1, sul2, tetA, tetX, tetG*	Culture-based methods/RT-PCR/Illumina NovaSeq 6000	Among ESBL genes, *blaTEM* (50.00%) and *blaCTX-M* (35.33%) were most prevalent, alongside *sul1* (58.67%), *aac(6')Ib-cr* (32.67%), *tetA* (24.00%), and *intI1* (58.67%), while *intI2*, *tetX*, and *aadA9* were absent from all isolates. *E. coli* and *K. pneumoniae* carried the majority of ARGs, averaging 2.8 and 4.2 determinants per isolate, respectively, with *E. coli* commonly co-harbouring *blaTEM, blaCTX-M, qnrS, sul1, sul2*, and *intI1*, and *K. pneumoniae* co-harbouring *blaTEM, blaSHV, sul1, sul2*, and *intI1*. Separately, the 10 carbapenemase-producing isolates carried 43 ARGs, with *blaNDM-1* the most common carbapenemase gene (8 isolates), followed by *blaOXA (2)* and *blaKPC (1),* while *blaIMP* and *blaVIM* were undetected. *sul1* was present in all ten isolates, followed by *tetA* (7/10), *blaTEM* (6/10), *ermB* (3/10), *qnrS* and *aac(6')Ib-cr* (2/10 each), and *blaSHV* and *sul2* (1/10 each), with *blaCTX-M*, *qnrA, aadA9, tetX*, and *tetG* absent throughout.	[421]
272.	September – October 2023	WWTP influent and effluent water 32 samples	Namibia: Windhoek	*aadA1, blaCTX-M-15, blaTEM-1, drfA8, sul1, and tet(B) in ST410; aac(6')-Ib-cr, aph(3'')-Ib, blaCTX-M-15, blaOXA-1, drfA17, sul1, and tet(B) in ST44; aac(3)-IIa, aac(6')-Ib-cr, aadA, aph(3')-Ia, arr-3, sul1, sul2, tet(A), tet(D), blaTEM-1-D, blaCTX-M-15, mphA, qnrS1, and blaSHV in the plasmid-borne group; aac(6')-Ib-cr, aadA, aadA2, aph(3')-Ia, aph(3'')-Ib, arr-3, blaCTX-M-15, dfrA12, sul1, mphA, qnrS1, fosA*	Culture-based methods/Illumina NextSeq 2000	Several high-risk clones were identified, including ST10 and ST410, with ST410 (recovered from effluent) harboring *aadA1, blaCTX-M-15, blaTEM-1, drfA8, sul1*, and *tet(B),* consistent with its phenotypic profile, while ST10 itself lacked detectable ARGs despite its clonal complex ST44 carrying multiple genes, including *aac(6')-Ib-cr, aph(3'')-Ib, blaCTX-M-15, blaOXA-1, drfA17, sul1*, and *tet(B).* Half of the isolates carried multiple plasmid-borne ARGs such as *aac(3)-IIa, aac(6')-Ib-cr, aadA, aph(3')-Ia, arr-3, sul1, sul2, tet(A), tet(D), blaTEM-1-D, blaCTX-M-15, mphA,* and *qnrS1*, with all isolates also carrying the chromosomal *blaSHV* variant. High-risk clones ST219 (influent) and ST20 (a hospital-associated clone, effluent) carried genes like *aac(6')-Ib-cr, aadA, aadA2, aph(3')-Ia, aph(3'')-Ib, arr-3, blaCTX-M-15, dfrA12, sul1, mphA, qnrS1, fosA,* while other clones such as ST1788 and ST515	[422]

						also harbored multiple ARGs.	
273.	January, February, June, July of 2020	Municipal WWTP Influent and effluent	Croatia: Zagreb, Zadar, Karlovac,<br><br>Vinkovci, Bjelovar, Čakovec and Varždin	*blaTEM, blaCTX-M-32, blaKPC-3, blaNDM, blaOXA-48-like, blaIMP and blaVIM*	Culture-based methods/RT-qPCR	RT-qPCR analysis revealed *blaTEM* in almost all samples, with higher abundance in summer than winter and only minor reductions after treatment. *blaCTX-M-32* was present in all winter influents but undetectable in winter effluents, while in summer it was consistently detected in both influents and effluents with no significant difference between them. *blaKPC-3* and *blaNDM* appeared only sporadically, mainly in winter and *blaOXA-48*-like was common in influents but rarely found in effluents. *blaIMP* was frequently detected in effluents despite being largely absent from paired influents and *blaVIM* showed variable patterns, generally more prevalent in summer influents, with inconsistent increases or decreases after treatment across plants.	[423]
274.	October – December 2019	River water	China: Lanzhou region, Yellow River	*ampC-01, blaAmpC-02, blaAmpC-04, blaAmpC-05, blaAmpC-06, blaAmpC, bla-DHA, bla-L1, blaIMP-1, blaIMP-2, mecA*	Standard PCR/HT-qPCR/Illumina MiSeq	The β-lactamase genes,blaAmpC-*01,blaAmpC-02,blaAmpC-04,blaAmpC-05,blaAmpC-06,blaAmpC, bla-DHA, bla-L1, blaIMP-1, blaIMP-2*, and *mecA* generally showed lower and more sporadic relative abundance compared to other ARG classes, with occasional elevated abundance appearing in isolated samples during summer and autumn.	[424]
275.	January and August 2018<br><br>January 2018 – January 2019	136 river water and 51 sediment samples from Xiaoqing River,<br><br>189 WWTP water samples	China: Jinan City	*blaCTX-M-14, blaCTX-M-15, blaCTX-M-27, blaCTX-M-55*	Culture-based methods/Standard PCR/Illumina NovaSeq 6000	*blaCTX-M-14* was the most common gene encoding third-generation cephalosporin resistance (42.9%, 9/21), followed by *blaCTX-M-55* (23.8%, 5/21), *blaCTX-M-15* (19.0%, 4/21), and *blaCTX-M-27* (9.5%, 2/21).	[425]

276.	2015	Surface sediments from lakes	China: Khanka Lake, Weishan Lake, Hungze Lake, Chao Lake, Suzhou Tai Lake, Wuxi Tai Lake, Huzhou Tai Lake, Yangcheng Lake, Poyang Lake, Dongting Lake, Dianchi Lake, Basongtso Lake, Qinghai Lake, Chaka Salt Lake and Namtso Lake	*blaCTX-M-1, blaCTX-M-2, blaCTX-M-3, blaCTX-M-4, blaSHV, blaTEM, strA, strB, tet(G), tet(K)*	Standard PCR/qPCR	Across the analysed samples *strA, strB blaCTX-M-4, blaSHV* and *blaTEM* genes were detected in every lake, with *blaCTX-M* as the dominant ESBL gene and *blaTEM* the most prevalent β-lactamase overall. Among tetracycline resistance genes, *tet(G)* and *tet(K)* showed higher detection frequency across the lakes, while genes for tetracycline, β-lactam, and aminoglycoside resistance were detected in all sediment samples. Poyang Lake and Dongting Lake showed the highest ARG positivity, with 75% of target genes (n=28) detected simultaneously, while several other major lakes exceeded 50% detection rates. In contrast, only eight ARGs were found in plateau lakes such as Basongtso and Chaka Salt Lake, with overall ARG distribution concentrated mainly along the middle and lower Yangtze River lakes.	[426]
277.	May – August 2021	River, lake and city moats<br>86 samples	China: Shandong province	*blaNDM, blaNDM-5, blaKPC-2, mcr-1*	Culture-based methods/Standard PCR/Illumina HiSeq	Of the 86 water samples, 14 (16.3%) carried carbapenemase genes, with seven positive for *blaNDM* and *blaKPC-2*. *blaNDM* was detected across all four cities, while *blaKPC-2* was found only in Binzhou (21.2%, 7/33), which also showed the highest overall carbapenemase detection rate (24.2%, 8/33), followed by Weifang (23.1%), Qingdao (20%), and Liaocheng (5.7%). The *mcr-1* gene was detected in 24 samples (27.9%) across all four cities, with 8 samples (9.3%) co-carrying *mcr-1* and carbapenemase genes. Among the 48 isolates (38 *E. coli*, 10 *K. pneumoniae*) recovered from the 30 *mcr-1* carbapenemase-positive	[427]

						samples, 36 *E. coli* isolates carried *mcr-1*, 12 carried *blaNDM-5*, and 10 of these carried both genes simultaneously. All *blaKPC-2*-positive isolates were CRKP, with no *blaNDM* or *mcr-1* detected in this group, and three samples contained both mcr-1-positive *E. coli* and *blaKPC-2*-positive CRKP.	
278.	March – May 2021	sea water, WWTP, surface waters, industrial effluent<br><br>162 samples	Tunisia: Monastir	*blaCTX-M-1, blaCTX-M-3, blaCTX-M-15, blaCTX-M-55, blaDHA-1, blaTEM, blaSHV, blaOXA-1, aph(6)-Id and aph(3")-Ib, sul1, sul2, tet(A)*	Culture-based methods/Illumina NovaSeq 6000	All isolates carried a *blaCTX-M-1* group ESBL gene, with *blaCTX-M-15* the most prevalent (56.4%, 22/38 isolates: 10 *E. coli*, 10 *K. pneumoniae*, 2 *C. freundii*), while *blaCTX-M-55* (n=8), *blaCTX-M-3* (n=7), and *blaCTX*-M-1 (n=2) were found only in *E. coli*, with the clonal ST349 *E. coli* isolates co-harboring *blaCTX-M-3* and *blaDHA-1*. Additional non-ESBL beta-lactamase genes included *blaTEM*-like (n=15), *blaSHV*-like (n=10), and *blaOXA-1* (n=9). Non-β-lactam resistance genes included *aph(6)-Id and aph(3")-Ib, and sul1, sul2, tet(A)*, consistent with the high phenotypic resistance rates observed for these antibiotic classes in both *E. coli* and *K. pneumoniae*, while no isolates were resistant to amikacin or colistin.	[428]
279.	February – March, July – August 2020	Hospital wastewater, WWTPs, river water upstream and downstream of WWTPs, swimming lakes<br><br>366 samples	Germany	*blaKPC-like, blaNDM-like, blaVIM-like, blaOXA-48-like, blaGES-like, blaIMI-like, blaNMC-A-like, blaIMP-like, blaGIM-like, khe, blaAmpC*	Culture-based methods/Standard PCR/Illumina NextSeq 550	Carbapenemase-producing isolates, predominantly from hospital wastewater, most frequently carried *blaKPC*-like (n=109), *blaOXA*-48-like (n=45), and *blaVIM*-like (n=37) genes, with 21 isolates harbouring more than one carbapenemase gene. WWTP effluents (plants 4 and 6) yielded 24 carbapenemase-positive isolates carrying *blaKPC-2* (n=9), *blaVIM*-like (n=8), *blaOXA-48*-like (n=5), and *blaNDM-5* (n=4), while river water samples showed *blaVIM-1* predominance upstream and *blaKPC-2* predominance downstream of WWTPs. No carbapenem-resistant bacteria were found in swimming lake samples. Overall, PCR detected carbapenemase genes in 243 of 287 isolates, with *blaKPC*-type genes present in half (124/243), followed by *blaVIM*-like (n=55) and *blaOXA-48*-like (n=52), while *blaNDM*-like, *blaIMI*-like, and *blaGIM*-like were rare (n=4, 2, and 2, respectively). 24 isolates	[429]

						carried multiple carbapenemase genes.	
280.	*N.R.*	River water samples	South Africa: North-flowing Crocodile West River	*blaAmpC*	Standard PCR/qPCR/Illumina MiSeq	Among all the sites, *ACT/MIR* was the most widely detected *blaAmpC* gene group (7/7), followed by *blaMOX/blaCMY* and *blaFOX* (6/7 each), *blaBIL/blaLAT/blaCMY* (5/7), *blaDHA* (4/7), and *blaACC* (1/7), with Site 1 showing the highest gene group diversity (6/6) and Site 7 the lowest (2/6). qPCR quantification revealed that *blaFOX* (16.04 copies/ng DNA) and *blaMOX/blaCMY* (27.57 copies/ng DNA) occurred at significantly higher copy numbers than other *blaAmpC* groups, and overall *blaAmpC* gene abundance was significantly higher at upstream sites (13.11 copies/ng DNA) than at downstream sites (0.47 copies/ng DNA).	[430]
281.	January – July, March – August 2016	WWTP wastewater samples,<br>river and canal<br>185 samples	U.S.A.: Washington State	*blaTEM, blaCTX-M-groups, blaPSE, blaCMY-2, blaKPC, blaOXA-48, blaNDM-1*	Standard PCR	Across all samples, *blaOXA-48* was the most frequently detected gene (57.3%), followed by *blaPSE* (52.8%), *blaCTX-M* (52.2%), *blaKPC* (42.7%), *blaCMY-2* (38.8%), and *blaNDM-1* (7.3%); in bacterial DNA fractions specifically, *blaOXA-48* remained most common (36.8%), followed by *blaTEM* (34.6%), *blaCTX-M* and *blaPSE* (32.4% each), *blaCMY-2* (30.8%), *blaKPC* (25.4%), and *blaNDM-1* (5.4%). All seven genes were significantly more prevalent in wastewater treatment plant samples than in combined river and canal samples.	[431]
282.	February 2020	WWTP effluent	Croatia: Zagreb	*blaCTX-M-1, blaCTX-M-3, blaCTX-M-15, blaCTX-M-55, blaTEM-1, blaTEM-116, blaTEM-135, blaSHV-1, blaSHV-12, blaSHV-28, blaGES-5, blaGES-7, blaLEN, blaEBC, blaDHA, blaACC, blaCIT, blaFOX, blaOXA-48, blaNDM-1, blaVIM, pAmpC*	Culture-based methods/Standard PCR/ION Torrent PGM sequencing	Among the 68 isolates, *blaCTX-M* (80.9%) and *blaTEM-116/135* (45.6%) were the predominant ESBL gene groups. Four *blaCTX-M*-subtypes were identified: *blaCTX-M-15* (n=41, mainly *E. coli*), *blaCTX-M-3* (n=9, mainly *K. cryocrescens*), *blaCTX-M-1* (n=4), and *blaCTX-M-55* (n=1), the latter two found only in *E. coli* alongside three *blaTEM* subtypes, *blaTEM-1* (n=35), *blaTEM-116* (n=30, mainly *E. coli* and *Klebsiella spp*.), and *blaTEM-135* (n=1, *E. coli* only). Less common β-lactamase genes included *blaSHV* (12.5%, with subtypes *blaSHV-1*, *blaSHV-12*, and *blaSHV-28*), *blaGES* (7.8%), and *blaLEN* (n=1). Sixteen isolates (23.5%) also carried *pAmpC* genes,	[432]

						predominantly *blaEBC* (n=11, mainly *E. cloacae* complex), along with *blaDHA, blaACC, blaCIT*, and *blaFOX* (n=1 or 2 each). Carbapenem resistance genes were less frequent (13.2%), including *blaOXA-48, blaNDM-1*, and *blaVIM-1* (1 or 2 isolates each), in addition to *blaGES-5*. Notably, 66.2% of isolates carried two β-lactamase genes, most commonly the *blaTEM-116/blaCTX-M* combination, predominantly in *E. coli* (n=13).	
283.	December 2020 – February 2021	Water samples	India: Ganga River	*acrA, mexA, adeI, smeD, mtrC, cmeA,* *mrcA, acrB, mexB, adeJ,* *smeE, mtrD, cmeB, ftsI, mrdA, nagZ, and tolC, ddl, alr, mraY, murG, murF*	Illumina MiSeq 2500	β-lactam resistance genes are not spread evenly across the Ganga River sites. The metallo-β-lactamase group, *bla2/blm/ccrA/blaB,* peaks dramatically at site AW1, and *blaAmpC* peaks just as sharply at AW2, while the penicillin-binding protein genes, *mrcA, mrdA, ftsI*, along with the regulatory gene *ampG*, cluster together as consistently elevated at site KW2 and moderately at AW1/KW3. By contrast, the HW1–HW3 sample sites are consistently low across almost all these genes, marking them as the least affected sites.	[433]
284.	June, September and October 2021	WWTP influent, treated water and effluent	Japan	*blaTEM, blaSHV, blaCTX-M-1, blaCTX-M-2, blaCTX-M-9*	Culture-based methods/Multi plex PCR	ESBL gene carriage among *E. coli* isolates was dominated by *blaCTX*-M-9 (62.9%) and *blaTEM* (47.2%), with *blaSHV* (15.7%) and *blaCTX-M-1* (25.8%) less common and *blaCTX-M-2* absent, and the *blaTEM/blaCTX-M-9* combination being the most frequent pairing. Carriage patterns shifted seasonally, with *blaCTX-M-9* and *blaTEM* dominating June influent samples, total gene carriage rising by September, and carriage dropping by November to only *blaCTX-M-9* in the effluent. Among non-*E. coli* ESBL-producing coliforms, *blaCTX-M-9* was again the most prevalent genotype overall (42.2%), followed by *blaSHV* (34.1%), *blaTEM* (31.9%), *blaCTX-M-1* (23.0%), and *blaCTX-M-2* (15.6%).	[434]
285.	2022	River water,	Thailand: Bangkok	*blaVIM, blaNDM, blaTEM, sul1, mcr-1, tetQ*	qPCR/ddPCR	Gene concentrations were consistently highest in WWTP influents, lower in effluents, and lowest in river water, with *intI1* the most abundant gene at every stage (~7.89, 6.67, and 5.60 log10	[435]

		WWTP influent and effluent water				copies/100 mL, respectively), followed broadly by *blaTEM, sul1,* and *tetQ*. At the same time, *blaVIM, mcr-1*, and *blaNDM* were consistently the least abundant. Detection was universal (100%) for all genes in influents and river samples except for select genes with partial detection, *blaNDM* was missing from one influent (91.7%) and most effluents (25%), and in river water *mcr-1* (25%), *blaVIM* (66.7%), *blaTEM* (91.7%), and *blaNDM* (8.3%) each showing reduced detection frequency.	
286.	May – July 2023	Drinking water	Ethiopia: Addis Ababa	*blaTEM, blaSHV, blaCTX-M, blaNDM, blaKPC, blaOXA-48*	Standard PCR	Conventional PCR analysis revealed that *blaKPC* and *blaNDM* genes were each detected in 50% of isolates, while the *blaOXA-48* gene was found in 25%. Among ESBL-producing *E. coli* O157:H7 isolates, *blaCTX-M* and *blaTEM* were equally prevalent (85.0% each), while *blaSHV* was detected less frequently overall. Similarly, carbapenem-resistant isolates showed equal detection rates for *blaNDM* and *blaKPC* (50.0% each). Geographically, *blaTEM* and *blaCTX-M* were more common in wastewater isolates from Akaki Kality (7 isolates each) compared to Addis Ketema, and carbapenem resistance genes were also predominantly found in Akaki Kality, with *blaNDM* and *blaKPC* each detected in 2 isolates there, while *blaOXA*-48 appeared in 1 isolate from each sub-city.	[436]
287.	January 2018 – February 2019	Sewage Sludge<br>324 samples	China: Jinan	*blaKPC*-, *blaNDM*-, *blaIMP*- and *blaOXA*-48-types	Culture-based method/PCR and Sanger sequencing	From environmental samples *R. ornithinolytica* and *R. planticola* were isolated. The genes detected in this study were: *blaKPC-2* (n = 13), *blaNDM-1* (n = 8), *blaNDM-5* (n = 1) and *blaIMP-4* (n = 1).isolates carried both *blaKPC-2* and *blaNDM-1*, 1 isolate co-harbored *blaKPC-2* and *blaNDM-5*, and 1 isolate carried both *blaKPC-2* and *blaIMP-4*.	[437]
288.	March 2017 – July 2018	Wastewater<br>Slasughterhoues<br>Water	Germany	*blaSHV*, *blaTEM*, *blaCTX-M*	Standard PCR	The samples were positive for antimicrobial-resistant bacteria (95.5% slaughterhouses samples and 97.2% municipal WWTPs). The following isolates were analysed: *E. coli* (n = 321), *Klebsiella spp.* (n = 142), *E. cloacae* complex (n =	[438]

		bodies 103 samples				29), and *Citrobacter spp.* (n = 9).<br>The most abundant genes in *E. coli* isolates in the slaughterhouse samples were *blaCTX-M* (76.3%), *blaTEM* (11.6%) and *blaSHV* (2.2%), followed by *blaSHV-12* (4.2%). From the mWWTPs samples (n = 131), exhibited a *blaCTX-M* genotype (78.6%), *blaTEM-1* (9.2%). Only one isolate (0.8%) was shown to carry *blaSHV-12*.<br>In isolated *K. pneumoniae* (61.4%), *blaCTX*-M-*1* in combinations with *blaSHV-1/-11/-27/-60* or *blaTEM-1B*. The *blaCTX-M-15*, alone or in combination with *blaSHV-1/-11/-28/-133* and/or *blaTEM*-1A/1B, was detected in 32.4% of the isolates. In the mWWTPs were also found *blaSHV*-31 (7.5%) and *blaSHV-69* (5.0%). Only 9.1% of *K. oxytoca* isolates (from mWWTP were carriers of *blaCTX*-M-9 and 4.5% of *blaCTX-M-1*/*blaSHV-1*/*blaTEM-1B*. Among the isolates of the *E. cloacae* complex in S1/S2, only 41.7% had *blaCTX-M-1,* while the isolates in WWTP showed *blaCTX-M-15* (29.4%). Among the isolates of the *E. cloacae* complex in S1/S2, 41.7% had *blaCTX-M-1*. While among the *Citrobacter spp.* isolated from slaughterhouses, all had *blaCTX-M-1* or in combination with *blaTEM-1* and those from mWWTP were positive for *blaCTX-M-15* (29.4%). Two out of three *Citrobacter spp.* isolates from WWTPs wore *blaCTX-M-15* and *blaSHV-12*.	
289.	August – September 2020	Wastewater	Indonesia: Surabaya	*blaOXA*-23-like ,*blaOXA*-24-like *blaOXA*-48-like *blaNDM*-1, *blaVIM*, *blaIMP*-1, *blaOXA*-23-like, 2-*blaOXA*-24-like, *blaOXA*-48-like,*blaNDM*-1,*blaVIM*, *blaIMP*-1), *blaOXA*-24-like, *blaOXA*-48-like,*blaNDM*-	mPCR	In the environmentsamples *Pseudomonas aeruginosa* and *Pseudomonas spp.* have been detected. Also, gene detection in those samples revealed *blaOXA-23-like* (13%), *blaOXA-24-like* (11%), *blaOXA-48-like* (5%), *blaNDM-1* (34%), *blaVIM* (32%), and *blaIMP-1*(11%). *blaOXA-58-like* was not detected in any sample.	[439]

				1,*blaVIM*,*blaOXA*-23-like,*blaNDM*-1, *blaVIM*,*blaIMP*-1,*blaOXA*-24-like, *blaNDM*-1, *blaVIM*, *blaIMP*-1, *blaNDM*-1, *blaVIM*, *blaNDM*-1			
290.	October – December 2017	Influent wastewater 15 samples	Japan: Matsumoto City, Nagano Prefecture	*blaSHV*, *blaOKP*-A/*blaOKP*-B, and *blaLEN*,	Culture-based method/PCR/Sequencing	Colistin-resistant *Klebsiella spp.* isolates have been identified of which nine isolates were *K. pneumoniae*, and one was identified as *K. quasipneumoniae*. In the *K. pneumoniae* isolates gene detection revealed *blaSHV*-187, *blaTEM*-1, *oqxA*, *oqxB*, *fosA*, *aadA2*, *aac(3)-IId*, *msrE*, *mphE*, *sul1*, *sul2*, and *dfrA12* in addition to *blaCTX-M-3* and *armA*. They were negative for *tet(A)*, *tet(M)*, and *tet(X)* genes.	[440]
291.	June 2017 – May 2018 (except January and April 2018)	Wastewater grab samples 10 samples	Bangladesh: Dhaka	*blaCTX*-M-15, *blaTEM*, qnrS, qnrB, dfrA1, dfrA17, *blaSHV*, sul2, sul1	Culture-based method/PCR	There have been thirty-six isolates of MDR *E. coli*. The most predominant gene identified was *qnrS* (32.43%), followed by *blaTEM* (29.73%), *blaCTX-M-15* (24.32%), *dfrA17* (21.62%), *sul2* (8.10%), *qnrB* (5.41%), *sul1* (5.41%), *dfrA1* (2.70%). *blaSHV* was not detected.	[441]
292.	26 November to 28 November, 2019.	River water	China: Minjiang River	*sul-1, qacH_351, ere(A), ere(B), cefa_qacelta, aadA21, blaVEB, aadA_99, aadA17, blaOXY-1, qepA_1_2, aadA5, ermF, erm(35), aac(6′)I1, aac(6′)-Is, blaHERA, blaSHV-11, blaVIM, tetPB, tetH, tetB, tet(36), tolC, acrR, adeA, terW, vgaB, mphB, erm (36),*	HT-qPCR	The concentration of the *blaTEM* gene was 2.14 × 10^{-2} copies/16S rRNA gene, being considered as one of the most prevalent genes in environmental habitats (main stem, aquaculture water, behind the dam, downstream of the dam, and urban river waters)	[442]

				qnrB46, *47*, *48*, and *qnrA*)			
293.	2015 and 2016	Freshwater Lakes	India: Kashmir valley	*blaCTX*-M, qnrA, qnrB, qnrC, qnrD, qnrS and aac (6′)-Ib) genes	PCR/Sequencing	The prevalence of *blaCTX-M* in the sampling areas was 63.63% and 27.27% respectivley.	[443]
294.	December 2014–February 2015, March–May 2015, June–August 2015 and September–November 2015	Drinking water reservoir 24 samples	China: Yangtze River Delta, East	*sul1*, *sul2*, *sul3*, *blaOXA-1*, *blaOXA-2*, *blaTEM-1*, *qepA*, *qnrS*, *tetA*, *tetM*, *tetW*, *strA*, *strB*, *ermB*, *intI1*	Standard PCR	*Pseudomonas aeruginosa* was isolated from the collected samples. The highest abundance was found in *blaOXA-2*, *qnrS*, and *tetW* were (91%). The intI1 gene was detected in all collected samples.	[444]
295.	2021	Hospital wastewater samples	Brazil: Ribeirão Preto São	*blaGES-16*, *blaSHV-5*, *blaTEM-1A*, *blaOXA-9*, *blaOKP-B-18*, *blaOKP-B-41*, *blaPRC-1*, *aph(3')-IIa, aph(3')-VIa, aph(3')-XV, aadA1, ant(3'')-Ia, abaQ, norB, oqxA, oqxB, macA, macB, mph(A), tet(B), tet(C)* *fosA, abaF, sul1, dfrA22, cat3*	Illumina NovaSeq6000 platform	Several genes were detected in *Klebsiella quasipneumoniae*: *blaGES-16*, *blaSHV-5*, *blaTEM-1A*, *blaOXA-9*, *blaOKP-B-18*, *blaOKP-B-41*, *blaPRC-1*	[445]
296.	N.R.	Hospital wastewater	Germany	*N.R.*	Culture-based method/	The isolates contained up to six different types of β-lactamase genes, including combinations of	[446]

		samples<br>36 samples			Sequencing	extended-spectrum β-lactamases (ESBLs), *blaAmpC* β-lactamases and one or more carbapenemases. Some of these carbapenemases, such as *blaOXA-48*, were found alongside *blaNDM-1* or *blaVIM-1*, while *blaSHV-12*, *blaTEM-1*, *blaCTX*-M-*15*, *blaOXA-9*, *blaTEM-1*, *blaNDM-1*, *blaCTX-M-15*, *blaOXA-1* and *blaSHV-12* were found in *C. farmeri* isolates. E. roggenkampii carried genes such as *blaTEM*-1, *blaCTX-M-15*, *blaNDM-1*, *blaVIM-1*, *blaACT-2*, and *blaOXA-1*. *K. oxytoca* had *blaSHV-12* present. *Serratia marcescens* contained genes such as *blaTEM-1*, *blaSHV-12*, *blaOXA*-1, *blaOXA-2*, *blaACC-1*, *blaSRT*, *blaCTX-M-9* and *blaCTX-M-1*.	
297.	May – September, 2022	Wastewater from pharmaceutical<br>Industries, hospitals, municipal sewage<br>And rivers<br>35 samples	Nepal: Kathmandu Valley, Bagmati River	*blaTEM, blaCTX-M and blaSHV,*<br>*intI1, intI2 and intI3, sul1, tetA, qnrS, aac(6')-Ib-cr* and *ermB*	Culture-based method/ RT-PCR<br>Sequencing: Illumina NovaSeq PE150	*blaTEM* was frequently identified in *Klebsiella* (46.27%, 31/67) and *E. coli* (23.88%, 16/67), being the most present in pharmaceutical influent (32.84%, 22/67) and hospital influent (19.40%, 13/67). *blaCTX*-M was detected in 50 isolates (*E. coli* and *Klebsiella* species), with a prevalence of 30.00% (15/50) in hospital influent and 26.00% (13/50) in river water. *blaSHV* was present in 39 isolates, having a high prevalence in pharmaceutical (28.21%, 11/39) and hospital influents 25.64%, 10/39). *blaSHV* was frequently detected in *Klebsiella* (71.79%, 28/39) and *Enterobacter* species (10.26%, 4/39).<br>Sequencing revealed that isolates mainly carried *blaTEM*-1 (n = 6), *blaCTX*-M-15 (n = 5), *blaSHV*-1<br>(n = 2), *blaSHV*-28 (n = 2) and *blaSHV*-66 (n = 3), *blaAmpC*, *blaDHA*-1, *blaGES*-23 and<br>*blaOXA*-10	[447]

298.	September, December 2019	WWTP effluent hospital wastewater<br>50 samples	Burkina Faso: Bobo-Dioulasso	*blaCTX-M-1, blaCTX-M-9, blaNDM-1, blaOXA-48*	Culture-based method/ Standard PCR	ESBL-producing isolates harboured *blaCTX-M-1* (99.14%, 116/117). Among these, 87 (90.6%) *blaCTX*-M-producing isolates were from the hospital, and 73 (86.9%) were from the WWTP. Hospital isolates also harboured *blaNDM*-1 (10.6%) and *blaOXA*-48-like (4.7%). 4 isolates (4.7%) from the WWTP harboured *blaNDM*-1.	[448]
299.	May – July 2020	Wastewate r	Iran: Tabriz	*blaTEM,*<br>*blaCTX-M, blaSHV, blaVIM, sul1, qnrS*	Culture-based method/ Standard PCR	*blaTEM* was present in 92% of the isolates tested, including 100% of the *E. coli* isolates. *blaVIM* was detected in only *Pseudomonas spp* strains. *Aeromonas*, *Ochrobactrum*, and<br>*Shigella* species harboured only *blaTEM*. *Escherichia albertii* and *Acinetobacter baumannii* harboured both *blaTEM* and *blaCTX*-M.	[449]
300.	April 2024	WWTP wastewater	Sri Lanka: Colombo, Kandy, Polonnaruwa and Kurunegala districts	*N.R.*	Sequencing: Illumina HiSeq 2500	The *blaOXA* (carbapenemase, ESBL or narrow-spectrum genes), *blaGES*, and *blaVEB* (ESBL genes), *blaTEM* (ESBL or narrow-spectrum genes) were the dominant subtypes. *blaKPC* was also dominant in Kan-WWTP. *Esherichia* was found to carry *blaSHV*, *blaGES*, and *blaOXA*, while *Pseudomonas* carried *blaOXA* and *blaVEB*.	[450]
301.	28, 30 January and 24, 26 July 2020	Hospital wastewater	Croatia: Zagreb	*blaTEM, blaCTX-M-32,*<br>*blaKPC-3, blaNDM, blaOXA-48-like, blaIMP, blaVIM,*<br>*mcr-1, 16S rRNA, (blaTEM,*<br>*blaSHV, blaPER, blaVEB, blaGES and blaSME) and singleplex PCR (blaCTX-M*<br>*groups 1, 2, and 9). In addition, these isolates*	qPCR (wastewater and isolates)	ESBL genes such as *blaTEM* and *blaCTX-M-32* and five carbapenemase genes (*blaKPC-3*, *blaOXA-48-like*, *blaNDM*, *blaIMP* and *blaVIM*) were identified in hospital wastewater samples. *blaTEM* and *blaCTX-M-32* concentrations ranged between 10^{-3} and 10^{-4} gene copies/rrn copies.<br>In isolates, the predominant ESBL gene was *blaCTX-M-15* (30/42), followed by *blaTEM-116* (22/42). *blaCTX-M-3* was detected in four isolates, while *blaSHV-12*, *blaSHV-28*, and *blaGES-7* were found only in *Klebsiella spp.* 19 isolates carried two ESBL genes, most commonly *blaCTX-M-15* + *blaTEM-116*. Carbapenemase genes were detected in 12/42 isolates, mainly *blaKPC-2* (6/42) and *blaOXA-48* (5/42), with	[451]

				underwent multiplex PCR<br><br>*for pAmpC genes (blaMOX, blaCIT, blaDHA, blaACC, blaEBC, and blaFOX, blaIMP, blaKPC, blaNDM, blaOXA-48, and blaVIM, mcr-1, mcr-2, mcr-3, mcr-4*<br><br>*and mcr-5*		*blaNDM-1* identified in one *K. oxytoca* isolate. Plasmid-mediated *blaAmpC* genes included *blaEBC* (6/42), *blaCIT* (6/42), and *blaMOX* (4/42).	
302.	October 2011 and August 2012	Influent wastewater	Finland: Helsinki	*blacTx*-M-1 group, *blaCTX*-M-2 and -5 group, *blaCTX*-M-25 group, *blaCTX*-M-8 group, *blacTx*-M-9 group, *blaACC*, *blaACT*, *blaCFE*, *blaCMY*/LAT, *blaFOX*, *blaLEN*,<br><br>*blaPSE*, *blaSHV*,<br><br>*blaVEB*, *blaTEM*, *blaDHA*, *blaOXY*, *blaMOX*, *blaMIR*, *blacARB*-5, *blaPER*, *blacARB*-6, *blaOKP*, *blaOXA*-1 group, *blaOXA*-10 group, *blaOXA*-2 group	RT-PCR	A total of 130 out of 399 isolates (32.6%) were positive for at least one β-lactamase gene. Of the 49 targeted genes, 22 were detected in at least one sample. The most common group was *blaGES* (25.8% of isolates), followed by *blaMOX* (13.1%), *blaTEM* (10.1%), *blaSHV* (9.6%), *blaMIR* (6.1%), the *blaCTX-M-1* group (5.1%), *blaACT* (5.1%), *blaPER* (5.1%), the *blaOXA-2* group (5.1%), the *blaCTX-M-9* group (4.0%), and the *KPC* group (3.0%), as well as other genes.<br><br>The predominant β-lactamase gene-carrying species were *A. hydrophila/caviae* (28.5%), *Enterobacter spp.* (16.9%), *E. coli* (14.6%), *K. cryocrescens* (10.0%), and *Aeromonas spp.* (6.9%). *E. coli* mainly carried ESBL genes (*blaCTX*-M groups and *blaTEM*), whereas *Enterobacter spp.*, *Aeromonas spp.*, and *K. cryocrescens* predominantly carried *blaAmpC* (*blaACT*, *blaMIR*, *blaMOX*) and/or *blaGES* genes.	[452]
303.	May – June 2022	Wells, boreholes, and rainwater tanks	Uganda: Mbarara	*blaTEM*, *blaSHV*, *blaCTX-M-32*, *blaCMY-2*, *blaOXA-48*, *blaVIM-2*, *blaNDM-1*, and *blaKPC-3*, *intl1*, *mcr-1*	Culture-based method/ Standard PCR	The genes *blaCTX-M-32* and *blaCMY-2* were found in 64% and 36% of the isolates, respectively. *blaOXA-48*, *blaVIM-2*, *blaNDM-1*, and *blaKPC-3* were present in only a small number of isolates. Only 2% of isolates were found to contain *blaTEM* and *blaSHV*.	[453]

304.	Winter and summer in 2019 and 2020	Wastewater influent, secondary effluent, final effluent, and treated biosolids	U.S.A.: Oregon	*N.R.*	Culture-based method/ Illumina MiSeq 3000 sequencing	ESBL-associated genes (*blaCTX-M*, *blaTEM*, *blaCMY*, and *blaSHV*) were detected in 10/11 ESBL-producing *E. coli* isolates, with five isolates carrying two ESBL genes (four with *blaCTX* and *blaTEM*; one with *blaCMY* and *blaTEM*). *blaCTX-M* was the most common (8/11), comprising *blaCTX-M-55* (n = 6) and *blaCTX-M-15* (n = 2). *blaTEM* was detected in five isolates, *blaCMY-42* in one isolate, and *blaSHV-12* in one isolate. Only one isolate carried a multidrug resistance-associated efflux pump adjacent to an ESBL gene. The *blaAmpC* gene was also present in all *E. coli* isolates.	[454]
305.	January, and August 2018	River, sediment<br>187 samples	China: Jinan	*blaNDM*	Culture-based method/ Standard PCR	*blaNDM-5* (n= 20) and *blaNDM-9* (n=2) genes were present in *E. coli* isolates	[455]
306.	June 2022 – July 2023	Hospital and municipal wastewater, treated wastewater	Uganda: Mbarara	*blaTEM, blaCMY-2, blaOXA-48, tetC, mcr-1, sul1*	RT-qPCR	*blaTEM*, *blaCMY-2* genes were detected in all samples, whereas *blaOXA-48* was present only sporadically in wastewater and the treated wastewater.	[456]
307.	December 2021<br>– October 2022	Ponds, lakes,<br>River, soil, aquatic weeds, drains, dumping yards, sewage treatment<br>Plants (stps), Cow	India: Delhi NCR	*blaSHV, blaTEM, blaOXA, blaCTX, int-1*, and *int-3*	Culture-based method/ Standard PCR	10 isolates harboured *blaTEM*, *blaCTX*, *blaOXA*, *blaSHV* genes. The tested genes (*blaTEM*, *blaCTX*, *blaOXA*, and *blaSHV*) were detected, with *blaTEM* being the most prevalent (29%), followed by *blaCTX* (25%), *blaOXA* (18%), and *blaSHV* (3%). Co-occurrence of *blaTEM* and *blaCTX* or *blaTEM* and *blaOXA* was observed in several isolates, including *E. coli*, *Aeromonas veronii*, *Bacillus cereus*, *Enterobacter cancerogenus*, *Pseudomonas mendocina*, and *Klebsiella pneumoniae*.	[457]

		dungs, cow urine and biogas slurry from various<br>Gaushalas (Cow shelters)<br>75 samples					
308.	February – November 2018	Wastewater treatment plant<br>168 samples	China: Jinan	*blaNDM, blaKPC, blaIMP, blaOXA and blaVIM, mcr-1, mcr-2,*<br>*mcr-3, mcr-4, and mcr-5.*	Standard PCR (Multiplex)<br>Sequencing: Illumina NovaSeq 6000	Carbapenem resistance genes were present in all isolates. *blaNDM-5* was the most prevalent, being present in 8 isolates. *blaNDM-4* was detected in one isolate. 5/8 isolates harboured *blaCTX-M* genes such as *blaCTX-M-14*, *blaCTX-M-15*, and *blaCTX-M-65*.	[458]
309.	July 15 – October 14,<br>2015	Wastewater treatment plant, foam from aerobic tanks	China: Binnong, Xianda, Xinnongji	*N.R.*	Sequencing: Illumina HiSeq platform	*blaOXA-46* was harboured by 13 bacterial genera, whereas *Pseudomonas aeruginosa* was found to carry the *blaNPS-1* and *blaOXA-205* beta-lactamase genes.	[459]
310.	*N.R.*	Hospital wastewater,<br>WWTP effluent, raw sewage<br>biofilm and sediments	Spain: Catalonia, Montsant River and Matarranya River	*blaNDM, blaOXA-48, blaKPC*	RT-qPCR	Carbapenemase genes were not detected in every sample. Real-time PCR showed all three target genes at high concentrations only in hospital effluents. Other environmental samples generally contained just *blaKPC* and *blaNDM*, except at Sau Reservoir, where all three fell below detection limits. *blaNDM* was abundant across nearly all samples, peaking in streambed biofilms from impacted rivers—3.52×10^7 copies g^{-1} (Matarranya River) and 2.31×10^6 copies g^{-1} (Montsant River). *blaKPC* was also present in biofilms (3.34×10^5–9.76×10^5 copies g^{-1}), but reached its highest level in the Trueta Hospital effluent (4.40×10^7 copies ml^{-1}). Treated wastewater had the lowest concentrations of both	[460]

						genes (3.91 × 10^2 and 1.87 × 10^4 copies ml^{-1}, respectively).	
311.	August – October 2019	Hospital wastewater	Nigeria: Ogun state	*blaCTX-M, blaKPC, blaNDM-1, blaTEM, blaSHV, blaCMY-1, blaAmpC*	Culture-based methods/RT-qPCR	All ESBL-producing isolates in this study were multidrug resistant, all carried *blaSHV*, and only six of sixteen carried *blaSHV* and *blaTEM* alone. Three isolates additionally carried *blaCTX-M-2, blaCTX-M-8,* and *blaCTX-M-25* variants alongside *blaSHV* and *blaTEM*, while none of the isolates harboured *blaCTX-M-1, blaCTX-M-9, blaKPC, blaNDM-1, blaCMY-1*, or *blaAmpC* genes. Overall, seven isolates (43.75%) carried *blaSHV* alone, six (37.50%) carried *blaSHV* + *blaTEM*, and three (18.75%) carried all three genes (*blaSHV* + *blaCTX-M* + *blaTEM*).	[461]
312.	*N.R.*	Soil, pound water, WWTP effluent	Botswana: Palapye, Mahibitswan	*blaSHV-11, blaSHV-81, blaSHV-172, blaSHV-180, blaOXA-217, blaOXA-50, blaTEM-206, blaLEN-24, blaCMY-98, and blaSED1, blaOKP-B-17, blaAmpC, cfxA6, blaTEM-1B, blaCTX-M, blaSHV-163, blaTEM-122, and blaOXA-663, ph(3)-Ib, aph(6)-Id, aac(2')-Ia, aac(6)-Iid, aph(3)-IIb, aac(6)-Ii, and aac(6')-Iz, sul1, sul2, oqxA, oqxB, drfA14.*	Culture-based methods/Illumina MiSeq	Community sources showed diverse resistance genes across classes, with pond water carrying the highest beta-lactam diversity (10 variants including *blaSHV-11, blaSHV-81, blaSHV-172, blaSHV-180, blaOXA-217, blaOXA-50, blaTEM-206, blaLEN-24, blaCMY-98, blaSED1, blaOKP-B-17*) compared to none in soil. Pond water also had the most aminoglycoside genes (*aph(3)-Ib, aph(6)-Id, aac(2')-Ia, aac(6)-Iid, aph(3)-IIb, aac(6)-Ii, aac(6')-Iz). sul1, sul2, oqxA, oqxB*, and *drfA14* were common across all three sources, while *mefA/mphA* (macrolides) appeared in soil and *catIII/catII/catB7* (phenicol) mainly in pond water. Among environmental sources, PWWTP effluent revealed seven beta-lactam genes (*blaAmpC, cfxA6, blaTEM-1B, blaCTX-M, blaSHV-163, blaTEM-122, blaOXA-663*), with *blaTEM-1B* detected in all sites except the effluent-receiving pond (which showed only *blaACT-9*).	[462]
313.	April 2019 – December 2020	Household environmental samples from hand-	Uganda: Malawi	*blaCTX-M-3, blaCTX-M-14, blaCTX-M-15,*	Culture-based methods/Illumina HiSeq	Of the 150 unique AMR genes identified, 20 were ESBL genes, dominated by *blaCTX-M-15* (68.4%), *blaCTX-M-27* (14.3%), and *blaCTX-M-14* (6.1%). ESBL gene distribution varied by	[463]

	July 2020 – August 2021	contact areas, food, water, river water		*blaCTX-M-27, blaNDM-5, pmrB, pmrB*		sequence type, with some (ST10, ST131) carrying multiple ESBL genes and others (ST3580) carrying only one, often linked to specific phylogenetic clusters. Among *Shigella spp*., *blaCTX-M-15* was similarly dominant (84.0%), followed by *blaCTX-M-3* (6.3%) and *blaCTX-M-27* (4.9%). The carbapenemase gene *blaNDM-5* was found in six genomes, all from a distinct ST167 cluster traced to a single household, while colistin-resistance mutations *pmrB* E123D (14.6%) and *pmrB* Y358N (28.8%) were widely distributed across multiple lineages and all three ecological niches.	
314.	14 – 16 October 2014;<br>1 – 10 September 2015	WWTP treated water,<br>upstream and downstream WWTP discharge	France, Italy, Norway, Portugal,<br>Germany, the Netherlands, Cyprus, Turkey, Austria and the United Kingdom	*blaTEM, blaOXA-48, blaOXA-58, blaCTX-M-15, blaCTX-M-32, blaKPC-3, sul1, tetM, mcr-1*	qPCR	Across 16 European WWTP effluents sampled over two campaigns, the following ARGs were analysed: *blaTEM, blaOXA-48, blaOXA-58, blaCTX-M-15, blaCTX-M-32, blaKPC-3, sul1, tetM*, and *mcr-1*. Gene concentrations were highly consistent between the two sampling campaigns, with 50% of measurements differing by no more than a factor of 2, and inter-campaign variation exceeding one order of magnitude occurring in only 8% of cases. This consistency extended across all WWTPs, allowing genes to be ranked by absolute abundance (copies/100 ml) as: *intI1 > sul1 > tetM > blaOXA-58 > blaTEM > blaOXA-48 > blaCTX-M-32 > mcr-1 > blaCTX-M-15 > blaKPC-3.* Limits of quantification ranged from 10^4/100 ml (16S rRNA and intI1) to 10^3/100 ml for the remaining genes. Notably, three genes—*blaOXA-48, blaKPC-3*, and *mcr-1*—were not detected in any effluent samples.	[464]
315.	November 2020 – July 2021	35 wastewater samples<br>35 drinking-	Pakistan: Sindh Province	*blaCTX-M*	qPCR	*blaCTX-M* group 1 genes were detected in two bulk drinking water samples and one drinking water biofilm sample. In wastewater, *blaCTX-M* group 1 was found in 13 bulk samples and 21 biofilm samples, with 69.2% of biofilms paired to positive bulk samples also testing positive. Overall, 54.3% concordance between bulk and	[465]

		water samples				biofilm samples showed no significant site-level association.	
316.	January 2022 – December 2023	48 water samples from 2 wwtps<br>46 sludge samples from 2 wwtps	Spain	*blaKPC, blaCTX-M, blaSHV, blaTEM*	Culture-based methods/Standard PCR	Among CR-E isolates, *blaKPC-2* was the dominant gene (93%, n=28), while *blaOXA-48-like* was found in two isolates from non-treated and pasteurised sludge that also co-harboured *blaCTX-M-15*. No ESBL genes were found in *blaKPC-2*-carrying strains, though five *blaKPC-2* isolates were detected in the effluent. Among ESBL isolates, *blaCTX-M-15* was most prevalent (46.5%, n=82), followed by *blaCTX*-M-14 (10.8%), *blaCTX-M-27* (8.5%), *blaCTX-M-32* (7.9%), and *blaSHV-12* (6.8%), alongside other non-ESBL β-lactamase genes (*blaTEM-1/35, blaSHV-1/11/27/28*). By source, 45.5% (n=80) of ESBL isolates came from raw water (carrying *blaCTX-M* variants *1/3/14/15/27/32/55/65/97* and *blaSHV-12*), 22.2% (n=39) from effluent (*blaCTX-M* variants *14/15/27/32/55, blaSHV-12, blaTEM-169*, including one *E. coli* co-harbouring *blaCTX-M-15* and *blaTEM-169*), and one *E. coli* carrying *blaCTX-M-15* was recovered downstream in the river. In sludge, 15.9% (n=28) of isolates came from non-treated sludge (*blaCTX-M* variants *1/14/15/27/32/55/97* and *blaSHV-12*), including co-harbouring strains (*blaCTX-M-15/blaOXA-244* in *E. coli*; *blaOXA-48/blaCTX-M-15* in *K. pneumoniae*), while amendment sludge yielded only 1.7% (n=3) of isolates, carrying *blaCTX-M-15/27*.	[466]
317.	*N.R.*	Water samples from 9 rivers and 4 streams	Brazil: Sao Paulo	*blaNDM, blaPER, blaSHV, blaGES, blaTEM, blaOXA-1, blaCTX-M-Gp1, blaCTX-M-Gp9, blaVEB, blaSPM, qnrA, qnrB, qnrS, qepA, aac(6')-lb-cr*	Standard PCR/Sequencing	Of the 13 water samples, *blaSHV* and *blaGES* were present in all (100%), while *blaTEM* and *blaCTX-M-Gp2* were found in 11 samples (84.6%). *blaCTX-M-Gp1*, *blaOXA-1*-like, and *blaKPC* each occurred in four samples (30.8%), whereas *blaPER*, *blaVEB*, and *blaOXA-48*-like were detected in two samples (15.4%). *blaCTX-M-Gp9* and *blaNDM* appeared only once, both in sample RW8, and *blaVIM* and *blaSPM* were absent altogether. Among the PMQR genes, *qnrS*	[467]

						and *qnrB* were each detected in 7 samples (53.8%), *qnrA* in 2 samples (15.4%), *qepA* in none, and *aac(6')-lb-cr* in 1 sample.	
318.	March 2021	Surface water samples from Thiruvandarkoil Lake	India: Tamil Nadu	*aac(6')-Ib-cr, aac(6')-Ib, aadA1, ant(2'')-Ia, armA, aac(3)-IId, blaTEM-1B, blaVEB-6, catA1, dfrA1, blaOKP-B-8, blaSHV-12, sul1, catA2, qnrB-1, aph(3'')-Ib, aph(6)-Id, blaNDM-1, blaCTX-M, blaACT-16, blaSHV-12,*	Standard PCR/Illumina TrueSeq	Resistome analysis showed *P. mirabilis* PS01 *aac(6')-Ib-cr, aac(6')-Ib, aadA1, ant(2'')-Ia, armA, aac(3)-IId, blaTEM-1B, blaVEB-6, catA1*, and *dfrA1* resistance genes. K. quasipneumoniae PS02 harboured *blaTEM-1B, blaOKP-B-8, blaSHV-12, sul1, catA2, qnrB-1, aph(3'')-Ib,* and *aph(6)-Id*, and *E. hormaechei* PS03 possessed multiple ESBL/carbapenemase genes *blaNDM-1, blaCTX-M, blaACT-16, blaSHV-12*, the colistin resistance gene *mcr-9*, and additional determinants for aminoglycosides, quinolones, sulphonamides, and chloramphenicol. Mobile genetic elements were widespread and often co-localised ARGs with virulence or metal-resistance genefor example, Tn3000 in PS03 carried both *blaNDM-1* and *qnrB-1*, sul2 was flanked by ISVsa3/IS5075, Tn6082 in PS02 carried *aph(3'')-Ib* and *aph(6)-Id*, and ISCfr1 in PS01 was linked to *aac(3)-IId*, highlighting genetic co-selection of resistance traits across the three isolates.	[468]
319.	November 2017	Coastal seawater from Bohai Bay	China: Tianjin	*blaOXA-58*	Culture-based methods/Standard PCR/RT-qPCR/Sequencing	Among the 36 carbapenem-resistant isolates screened for carbapenemase genes (*blaVIM, blaKPC, blaNDM, blaIMP, blaGES, blaOXA-23, blaOXA-24, blaOXA-48, blaOXA-51, blaOXA-58*), only *blaOXA-58* was detected, found in 16 isolates (44.4%) and located exclusively on plasmids, suggesting it is a major carbapenemase gene in coastal seawater.	[469]
320.	May – June 2007<br>July 2016<br>November 2015	Sediment samples from the Bering Sea	China	*blaOXA-1, blaTEM-1, blaOXA-2,*<br>*blaDHA-1, blaVIM-1, blaAmpC, blaCMY-2, blaOXA-10, blaSHV-1, blaGES-1, blaNDM-1,*	Culture-based methods//Standard PCR/qPCR	Of 30 ARGs assayed, 26 were detected in Arctic/sub-Arctic marine sediments, while *blaSHV-1, blaGES-1, blaNDM-1*, and *blaKPC* fell below the quantification limit. *sul1, sul2, sul3* were most prevalent (relative abundances of 2.45×10^{-7}–1.71×10^{-6}, 4.05×10^{-8}–6.36×10^{-6}, and 3.86×10^{-8}–1.51×10^{-6}, respectively), though 2–5	[470]

		Polar marine sediments<br>Surface sediment samples from the Qilihai Wetland, the Haihe River and the Tianjin<br>Water Park		*blaKPC, tetA, tetB, tetM, tetC, tetD, aacC2, aacC3, aacC4, ermC, qepA, qnrB, qnrA, qnrS, qnrD*		orders of magnitude lower than in human-impacted sites like the Haihe River (3.4×10^{-4}–1.1×10^{-2}). All six tet genes were detected ($\leq2.93\times10^{-6}$), with *tetM* and *tetC* dominant in human-impacted regions. Among aminoglycoside genes, *aacC2* (2.29×10^{-8}–2.24×10^{-6}) exceeded *aacC3* and *aacC4*. *qepA* and *qnrB* were the most abundant quinolone resistance genes, and *ermC* ranged from 7.01×10^{-8}–1.67×10^{-6}. Among 12 β-lactam genes, *blaOXA-1* and *blaTEM-1* were most prevalent (up to 4.33×10^{-6} and 4.78×10^{-6}, over two orders of magnitude below Haihe River levels), *blaOXA-2* appeared in four of seven samples, *blaAmpC* in three, *blaVIM-1* and *blaOXA-10* in two each, and *blaDHA-1* and *blaCMY-2* in single samples only, while *blaSHV-1, blaGES-1, blaNDM-1*, and *blaKPC* were undetected overall.	
321.	*N.R.*	Raw wastewater samples, secondary treatment water samples and effluent samples from UWWTP	Portugal	*blaL1, blaVIM, blaIMP, blaGES, blaKPC,*<br>*blaNDM, blaOXA-like, blaTEM, blaSHV, blaCTX-M, sul1*	Culture-based methods//Standard PCR/Illumina sequencing	PCR analysis detected *blaTEM* in 4 isolates, *blaGES-5* in all isolates, and *sul1* in 15 isolates, with *blaGES-5* linked to class 1 integrons in two isolates and class 3 integrons. Downstream of *intI1, blaOXA/aacA4* fusion, *blaGES-5*, and *blaBEL-1* were identified alongside conserved *attI/attC* sites and putative PcH1 and P2 promoters. In *C. braakii* RWM.4, *blaGES-5* was found on a 24,715 bp contig, roughly half of which shared 99.7% similarity with the *K. oxytoca* plasmid pKOR-e3cb, followed by a class 1 integron with an aacA4 cassette and a class 3 integron identical to that in RWM.8, including the *blaGES-5* cassette and a partial *blaOXA-10/aacA4* sequence. In *C. braakii* RWM.8, *blaGES-5* was located on the complete IncQ plasmid pRWM8, which carried a class 3 integron with two gene cassettes, *blaGES-5–blaOXA-10/aacA4*, whose *attC* motifs were identified. *blaSHV-1, blaGES-1, blaNDM-1*, and *blaKPC* fell below the quantification limit.	[471]

322.	March 2014 – January 2015	155 dug well water samples<br>115 spring water samples<br>22 estuary water samples around tripoli	Lebanon	*blaCTX-M, blaTEM, blaSHV, blaOXA-48, blaCMY, qnrA, qnrB, qnrC, qnrD, qnrS, oqxAB, acc(6')-Ib-cr, qepA*	Standard PCR/Multiplex PCR	Among 17 ESBL-producing bacteria from estuaries, *blaCTX-M-15* was the most prevalent gene, found in 14 isolates (82.4%: 10 *E. coli*, three *K. pneumoniae*, one *C. freundii*), while *blaCTX-M-55* occurred in one *E. coli*, *blaCTX-M-14* in three isolates, *blaSHV-12* alone in one and combined with *blaCTX-M-14* in another, and *blaCMY-42* in three *blaCTX-M-15*-producing *E. coli*. The four carbapenemase-producing isolates carried either *blaOXA-48* (alone in one *K. pneumoniae*, combined with *blaCTX-M-14* in one *E. coli*, both on IncL plasmids) or *blaOXA-244* (in two *E. coli*, one alone and one with *blaCTX-M-15/blaSHV-12,* both on IncHI2 plasmids). Additionally, *blaCTX-M-15* was found in 10 *E. coli* from wells and spring water, plus two *K. pneumoniae* and *C. braakii* isolates.	[472]

N.R. – Not reported
